# Transcriptomic analysis of crustacean neuropeptide signaling during the moult cycle in the green shore crab, *Carcinus maenas*

**DOI:** 10.1186/s12864-018-5057-3

**Published:** 2018-09-26

**Authors:** Andrew Oliphant, Jodi L. Alexander, Martin T. Swain, Simon G. Webster, David C. Wilcockson

**Affiliations:** 1 Institute of Biological, Environmental and Rural Sciences, Aberystwyth University, Aberystwyth, Ceredigion SY23 3DA UK; 2 School of Biological Sciences, Bangor University, Bangor, Gwynedd LL57 2UW UK

**Keywords:** Ecdysis, G protein-coupled receptor, Central nervous system, Y-organ

## Abstract

**Background:**

Ecdysis is an innate behaviour programme by which all arthropods moult their exoskeletons. The complex suite of interacting neuropeptides that orchestrate ecdysis is well studied in insects, but details of the crustacean ecdysis cassette are fragmented and our understanding of this process is comparatively crude, preventing a meaningful evolutionary comparison. To begin to address this issue we identified transcripts coding for neuropeptides and their putative receptors in the central nervous system (CNS) and Y-organs (YO) within the crab, *Carcinus maenas,* and mapped their expression profiles across accurately defined stages of the moult cycle using RNA-sequencing. We also studied gene expression within the epidermally-derived YO, the only defined role for which is the synthesis of ecdysteroid moulting hormones, to elucidate peptides and G protein-coupled receptors (GPCRs) that might have a function in ecdysis.

**Results:**

Transcriptome mining of the CNS transcriptome yielded neuropeptide transcripts representing 47 neuropeptide families and 66 putative GPCRs. Neuropeptide transcripts that were differentially expressed across the moult cycle included carcikinin, crustacean hyperglycemic hormone-2, and crustacean cardioactive peptide, whilst a single putative neuropeptide receptor, proctolin R1, was differentially expressed. Carcikinin mRNA in particular exhibited dramatic increases in expression pre-moult, suggesting a role in ecdysis regulation. Crustacean hyperglycemic hormone-2 mRNA expression was elevated post- and pre-moult whilst that for crustacean cardioactive peptide, which regulates insect ecdysis and plays a role in stereotyped motor activity during crustacean ecdysis, was elevated in pre-moult.

In the YO, several putative neuropeptide receptor transcripts were differentially expressed across the moult cycle, as was the mRNA for the neuropeptide, neuroparsin-1. Whilst differential gene expression of putative neuropeptide receptors was expected, the discovery and differential expression of neuropeptide transcripts was surprising. Analysis of GPCR transcript expression between YO and epidermis revealed 11 to be upregulated in the YO and thus are now candidates for peptide control of ecdysis.

**Conclusions:**

The data presented represent a comprehensive survey of the deduced *C. maenas* neuropeptidome and putative GPCRs. Importantly, we have described the differential expression profiles of these transcripts across accurately staged moult cycles in tissues key to the ecdysis programme. This study provides important avenues for the future exploration of functionality of receptor-ligand pairs in crustaceans.

**Electronic supplementary material:**

The online version of this article (10.1186/s12864-018-5057-3) contains supplementary material, which is available to authorized users.

## Background

Arthropods are characterised by their rigid exoskeleton that, although fundamental to their evolutionary success, must be shed periodically so that they can develop and grow. The moulting process, termed ecdysis, is achieved via an innate sequence of behaviours and stereotyped motor activity, precisely co-ordinated by the interaction of steroid moulting hormones (ecdysteroids) and a complex suite of interacting neuropeptides. The insect ecdysis programme is arguably the best understood endocrine cascade among all invertebrates and a consensus model is now emerging (reviews: [1, 2]). Briefly, new cuticle development is driven by ecdysteroids, which are synthesised and released from the prothoracic glands following stimulation by the brain-derived prothoracicotropic hormone (PTTH). Subsequently, ecdysteroids initiate ecdysis triggering hormone (ETH) production in so-called ‘Inka’ cells (tracheal endocrine cells) and induce central nervous system (CNS) sensitivity to ETH by promoting ETH receptor (ETHR) expression. Two alternatively spliced ETHRs (ETHR-A & ETHR-B) are expressed in discrete populations of neurons that are thought to be differently involved in pre-ecdysis and ecdysis [3–5]. ‘Inka’ cells, however only gain secretory competence when ecdysteroid titre declines below a threshold level after which they release ETH, which in turn induces eclosion hormone (EH) release from the CNS. Together, ETH and EH enter a positive feedback loop culminating in a massive release of both neuropeptides into circulation [6, 7]. In the tobacco hornworm, *Manduca sexta,* the neuropeptide corazonin (CRZ), a paralog of gonadotropin-releasing hormone, initiates the release of ETH from ‘Inka’ cells [8]. However, this is not the case in *Drosophila* and in some insects corazonin is entirely absent [9]. Elevated hemolymph titres of ETH and EH provides a clear ecdysis signal and commits the animal to the process by evoking release of the cyclic nonapeptide, crustacean cardioactive peptide (CCAP), which starts the ecdysis motor programme and terminates pre-ecdysial behaviours [10, 11]. Post-ecdysis cuticle maturation, including wing inflation and cuticle tanning, is regulated by the peptide bursicon, co-released with CCAP from a sub-set of CCAP neurons in the abdominal ganglia [12–14]. Our knowledge on the species-specific actions of ETH, EH, CCAP, and bursicon is under constant review, but the emerging picture is that whilst the functions of these neuropeptides are mostly conserved throughout insects, they may have subtly different roles in different taxonomic groups and at different developmental stages.

Whilst our understanding of the control of insect ecdysis has advanced in recent years, it is relatively poorly described in crustaceans. Nevertheless, the regulation of insect and crustacean ecdysis has a common origin in the neuroendocrine system of the crustacean-like ancestor from which these clades evolved [15, 16] and so crustacean endocrinology has benefitted from comparative work done on insects. In crustaceans, ecdysteroid synthesis occurs in Y-organs (YO) that are homologous with the prothoracic glands of insects, and via a common biosynthesis pathway [17]. Perhaps the defining difference between insect and crustacean cuticular development and ecdysis is that whilst ecdysteroids ultimately co-ordinate the moult cycle in both insects and crustaceans, ecdysteroid biosynthesis in the YO is under negative regulation by the eyestalk-derived neuropeptides, crustacean hyperglycemic hormone (CHH) and moult-inhibiting hormone (MIH) [18]. In crustaceans, CHH and MIH, which are part of the structurally related (but functionally diverse) CHH-superfamily [19] are synthesised in large perikarya concentrated in the so-called X-organ of the eyestalk medulla terminalis and released (in the case of MIH) in a pulsatile fashion by exocytosis from a coalescence of axons emanating from these cells [20], the sinus gland, into the hemolymph. After circulating in the hemolymph, CHH and MIH bind to specific receptors (putatively, GPCRs) on the YO to effect ecdysteroid inhibition [18] during intermoult stages (stages C-D1). Hence the discovery in 1953 in fiddler crabs (*Uca*) that removal of eyestalks releases this inhibition and can lead to precocious moulting [21]. The temporal release of CHH and MIH implicated in ecdysteroid inhibition in crustaceans is complicated by species specificity and we currently lack a consensus model of the events leading to moulting. This issue is discussed in detail by Webster (2014) [18]. Whatever the precise order of events, YO inhibition must be released in premolt and is followed by elevated ecdysteroid synthesis in stages D1-D3. Downstream of ecdysteroid synthesis our understanding of crustacean moult control is fragmentary at best and there is currently no consensus on the endocrine cascade that culminates in ecdysial behaviour and cuticle tanning. Thus far we know that, during ecdysis (stage E) CHH is dramatically released from paraneurons in the fore and hind-gut whilst CCAP and bursicon are co-released from the pericardial organs, and concomitant with the onset of ecdysis motor programme that precedes post-ecdysial tanning and cuticle hardening [18, 22] (during stages A-C). Thus, the activities of CCAP and bursicon may be generally conserved between crustaceans and insects [18, 22]. Broadly then, our knowledge of the neuropeptides involved in the regulation of crustacean ecdysis, such as CCAP and bursicon, indicate that some elements are conserved across arthropods whilst others are strikingly different (e.g. ecdysteroid regulation). Our inadequate understanding of key elements (and their roles) of the neuroendocrine control of crustacean ecydsis precludes a meaningful evolutionary comparison with the process in insects.

Until recently, the discovery and characterization of neuropeptides relied on the laborious purification of native peptides from animal tissues coupled with manual peptide sequencing or mass spectrometry (e.g. [23, 24]). Today, next generation sequencing (NGS) strategies have become routine and relatively inexpensive and coupled with emerging informatics pipelines have accelerated peptide mRNA transcript discovery and characterization [25]. This new era of whole transcriptome sequencing has generated a surge in neuropeptide discovery in non-model species, including crustaceans, overcoming their previous genetic intractability (e.g. [26–29]). A consequence of this post-genomic era is a widening gap between the description and characterization of conceptual peptide genes and their cognate peptide products and in vivo functions. Through NGS and transcriptome mining within crustaceans, orthologs of insect neuropeptides have been documented [27], but functional roles for these peptides remain lacking. Similarly, whilst the receptors for numerous neuropeptides have been functionally demonstrated for insects, only a few G protein-coupled receptors (GPCRs) have been de-orphanised among crustaceans [30, 31] and so identification of their GPCRs lags considerably behind. Indeed, only recently have efforts been focussed on revealing the structural identity of putative GPCRs in a crustacean, the spiny lobster *Sagmariasus verreauxi* [32]. Here, we sought to shed light on the involvement of neuropeptides in crustacean ecdysis by using RNA-sequencing to identify neuropeptides and putative neuropeptide GPCRs in the CNS and YO of the green shore crab, *Carcinus maenas* and against the insect system as a framework for our exploration. Thus, we describe a comprehensive suite of neuropeptide and receptor transcripts in *C. maenas* and define their expression profiles across the moult cycle at precisely defined moult stages. This study is the first exploration of a multi-tissue crustacean neurotranscriptome across the moult cycle with high temporal precision and therefore provides the opportunity to elucidate putative neuropeptide signaling during the crustacean ecdysial programme and draw comparisons with the current insect model.

## Methods

*Carcinus maenas* were collected from the Menai Straits, UK using baited traps and held in a flow through aquaria at Bangor University until use. Aquarium conditions were: water temperature = 16 °C, salinity =35 (full seawater), light:dark cycle = 12:12. Aquarium conditions did not replicate ambient tidal cycles. Crabs were fed ad libitum twice per week and were kept for no more than 4 weeks. Crabs were collected and dissected throughout the spring and summer months when moulting is prevalent.

### Total RNA extraction, cDNA library preparation, and sequencing

Total RNA was harvested from crabs dissected across the moult cycle at stages A-B, C3–4, D1, D3, D4 (stages according to Phlippen et al. [33]) for *n = 5* animals (biological replicates) per moult-stage. Three sets of tissues were collected for transcriptome sequencing and were subsequently analysed independently due to the considerable volume of data which sequencing yielded (see Additional file 1: Table S5, S6, and S7, for further details on sequenced data sets). The first consisted of central nervous system tissues (CNS: eyestalk ganglia [ESG], cerebral ganglion [CG], and ventral ganglion [VG] comprising the fused sub-oesophageal [SOG], thoracic [TG], abdominal ganglia [AG]); the second, pairs of Y-organs (YO); and the third consisted of inter-moult (C3–4) pairs of YOs and epidermis from the branchiostegite region of the carapace (overlying the pleural suture posterior of the YO). These tissues were dissected from ice-anaesthetised *C. maenas* under ice-cold physiological saline [34], using dedicated instruments for each tissue. Tissues were snap frozen in liquid nitrogen before storage at − 80 °C until use. Tissues were homogenised in TRIzol reagent in a Qiagen TissueLyser LT (Qiagen, UK) for 1 min (CNS and YO tissues) or 3 min (epidermis) at 50 Hz with a 5 mm stainless steel bead and RNA extracted according to the manufacturer’s protocol except additional ethanol washes were performed prior to resuspension of the RNA pellet in DEPC-treated water. RNA integrity was assessed by gel electrophoresis (Additional file 1: Figure S1) whilst yield and purity were measured spectrophotometrically with a NanoDrop ND2000**™** (Thermo Scientific, UK). cDNA library preparation was done using Illumina TruSeq RNA sample preparation reagents according to the manufacturer’s protocol. For CNS tissue cDNA library preparation, equal quantities (1 μg) of total RNA taken from separate neural tissues (ESG, CG, VG) were pooled. Library quality was assessed by gel electrophoresis and quantified using a Qubit 2.0 Fluorometer (Invitrogen**™**, Thermo Fisher, UK) prior to sequencing. Paired-end 126-bp reads were sequenced across multiple lanes on an Illumina HiSeq 2500 platform. Library preparation, quality checks and sequencing were done in-house at the Translational Genomics Facility, IBERS, Aberystwyth University.

### Transcriptome assembly, characterization, and differential gene expression analysis

Comparisons of various de novo assembly software packages demonstrate that no single assembler is universally superior [35, 36]. To thoroughly evaluate the potential of the sequenced raw reads, multiple assemblers were used for de novo transcriptome assembly of CNS reads and the resulting transcriptomes compared: Trinity v2.0.6 [37], Oases v0.2.8 [38] (which used Velvet v1.2.10 [39]), IDBA-tran v1.1.1 [40], and BinPacker v1.1 [41]. Prior to assembly, a 13-bp head crop of raw reads was done [42] followed by trimming and in silico read normalisation using the Trinity options --trimmomatic [43] and --normalize_reads. Both options were run with default settings (trimmomatic: phred33, SLIDINGWINDOW:4:5 LEADING:5 TRAILING:5 MINLEN:25, based on [44]). Trimmed and  normalised reads were assembled with default settings in each of the four assemblers (k-mer length: Trinity k = 25; Oases k = 23, 27, 31; IDBA-tran k = 20, 30, 40, 50, 60; BinPacker k = 25). Comparisons of these four transcriptome assemblies revealed the Trinity assembly to be superior in terms of the number and completeness of *C. maenas* neuropeptide transcripts assembled as well as number and completeness of UniProt/Swiss-Prot BLAST hits (for details see Additional file 1: Table S1, Figures S2 and S3). Consequently, Trinity was used for the assembly of transcriptomes in the current study. Transcriptomes for CNS and YO tissues sampled across the moult cycle, and for inter-moult (C3–4) YO and epidermis tissues, were assembled separately. For each transcriptome, Trinity transcripts were clustered into ‘genes’ using Corset v1.06 [45], which clusters transcripts based on sequence similarity and read counts (generated by Bowtie v1.0.1 [46] with multi-mapping enabled). These Corset-clustered Trinity transcriptomes (herein referred to as the *Carma*_CNS-transcriptome, *Carma_*YO-transcriptome, and *Carma_*YO_vs_Epi-transcriptome) were annotated using Trinotate v2.0.1 and associated software (TransDecoder v2.0.1, BLAST v2.2.31+, HMMER v3.1, SignalP v4.1, TMHMM v2.0, RNAMMER v1.2, TrinotateR). The process of Corset clustering, which used Bowtie generated mapping data, outputs a counts file for use in differential expression analysis [45]. For *Carma_*CNS*-* and *Carma_*YO-transcriptomes, differential cluster expression was performed using the edgeR package [47] (with the glm method to enable multiple comparisons) and by comparing inter-moult (C3–4) stage count data with that from each of the other four moult stages (A-B, D1, D3, and D4). For the *Carma_*YO_vs_Epi-transcriptome, differential cluster expression was performed by comparing count data between YO and epidermis tissues, again using the edgeR package [47]. Bowtie mapping, Corset clustering and counting, and edgeR differential expression analysis followed the example Corset pipeline at: github.com/Oshlack/Corset/wiki. The three transcriptomes assembled as part of this Transcriptome Shotgun Assembly project have been deposited at DDBJ/EMBL/GenBank under the accessions: GFXF00000000 (*Carma_*CNS-transcriptome), GFYV00000000 (*Carma_*YO-transcriptome), and GFYW00000000 (*Carma_*YO_vs_Epi-transcriptome). The transcriptomes have also been made available as publically accessible BLAST databases at: sequenceserver.ibers.aber.ac.uk. Raw read sequence files used to generate these assemblies are archived in the NCBI SRA archive under BioProject PRJNA400568. Trinotate annotation reports for *Carma_*CNS-, *Carma_*YO-, and *Carma_*YO_vs_Epi-transcriptome are provided in Additional file 2 as well as transcripts for neuropeptides and putative neuropeptide receptors mined from *Carma_*CNS- and *Carma_*YO-transcriptomes.

### Neuropeptide and receptor discovery

The *Carma_*CNS- and *Carma_*YO-transcriptomes were mined for contigs coding for putative neuropeptides using local tBLASTn searches with sequences from *C. maenas* and related species as search terms (performed in BioEdit software, [48]). Short protein motifs in open reading frames (ORFs) identified by TransDecoder were also searched in BioEdit. Peptide sequences used as search terms were taken from the NCBI [49] database and from recent literature [26, 27, 50, 51]. BLAST e-value and sequence identity thresholds considered significant between query peptide sequences and BLAST hits were assessed case-by-case. Contigs mined as putative neuropeptides were translated using the online tool ExPASy Translate (web.expasy.org/translate, [52]) and signal peptides identified with SignalP v.4.1 Server (set to ‘Sensitive’ to reproduce SignalP v.3.0’s sensitivity, [53]). Convertase cleavage sites were predicted following [26, 51, 54] and basic amino acid residues removed by carboxypeptidase were identified. Neuropeptide prepro-hormone processing and deduced mature peptides and precursor-related peptides (PRPs) have been made available in Additional file 3. Cysteine-cysteine disulfide bridges were predicted with the online tool: disulfind.dsi.unifi.it. Contigs coding for putative neuropeptide GPCRs were also mined using local tBLASTn searches with sequences taken from the NCBI database for *Drosophila* and *Tribolium casteneum*. Whilst there are studies which have sought to identify crustacean neuropeptide GPCRs based on sequence similarity to known neuropeptide GPCRs (e.g. [29, 32, 51]), until recently no crustacean neuropeptide GPCR had been de-orphanised [30, 31]. For this reason predicted receptors were identified as a result of searching using search terms for *Tribolium casteneum * and *Drosophila* receptors, for which functional de-orphanisation has been achieved. GPCR transmembrane helix domains were predicted using TMHMM server v.2.0 (cbs.dtu.dk/services/TMHMM, [55, 56]). Amino acid alignments were done in BioEdit via the ClustalW method [57]. Phylogenetic trees were constructed using the Neighbor-Joining method (1000 bootstrap replicates) [58] in MEGA6 [59]. *Drosophila melanogaster* metabotropic Glutamate Receptor (CG11144 mGLuR) was used as an outgroup to root phylogenetic trees for class A (rhodopsin-like), class B (secretin-like), and leucine-rich repeat-containing GPCRs (LGR) trees. *Drosophila melanogaster* trissin (NP_650471.1) was used as an outgroup to root the tree for the ETH, carcikinin, and carcikinin-like peptides phylogenetic tree.

### Quantitative RT-PCR

Quantitative RT-PCR assays were developed and performed according to previously published methods [60] using Taqman**™** MGB probes and in close adherence to MIQE guidelines [61]. In brief, standard curves were made by generating complementary RNA in vitro with T7 RNA polymerase (MegaShort Script**™**, Ambion**®**, UK) using PCR derived DNA templates amplified with T7 phage promotor sequence flanked gene specific primers. All oligonucleotide sequences for cRNA preparations and Taqman assays are detailed in Additional file 1: Table S2. Complementary RNA was purified on 10% 6 M urea PAGE gels and eluted in Elution Buffer (Ambion**®**, Thermo fisher, UK) overnight at RT before ethanol precipitation. Resulting cRNA was quantified spectrophotometrically, converted to copy number using Avogadro’s constant and diluted in DEPC-treated water and stored at − 80 °C until use. Standard curves were run in the range 10^9^ to 10^3^ copies per reaction. Sample and standard RNA was reverse transcribed using Tetro**™** reverse transcriptase (Bioline, UK) according to the manufacturer’s instructions and diluted in water 1:10 for PCR. Assays were run in triplicate on an Applied Biosystems**®** Quant Studio 12 Flex platform (Thermo-Fisher Scientific, UK) in 10 μL volumes using Sensimix Fast II probe**™** qPCR mix with ROX internal reference dye (Bioline, UK). Data were expressed as copies of target mRNA normalized to the geometric mean of the reference genes *elongation factor 1-alpha* (EF1a) and *ubiquitin-conjugating enzyme* E2 L3 (UBE2L3) shown to be constitutively expressed across all moult stages with RNAseq analysis (Additional file 1: Figure S4).

### End-point PCR

End-point PCRs were performed for cDNAs encoding eclosion hormones 1 and 2, crustacean hyperglycemic hormone 2, and the reference gene *elongation factor 1-alpha*, to establish the tissue distribution of these transcripts. Tissues were dissected, and total RNA extracted, and RNA reverse transcribed for 5 inter-moult (C4) crabs as outlined above. cDNA of *n* = 5 individuals was pooled for end-point PCR, which was performed using Bioline myTAQ red mix on a Bio-Rad T100 thermal cycler. PCR conditions were: denature at 95 °C for 3 mins, then 35 cycles of 30 s at 95 °C, 45 s at 60 °C, and 45 s at 72 °C, followed by extension at 72 °C for 5 mins. PCR products were resolved on 1% agarose gels.

## Results and discussion

Transcriptome assembly and annotation summary data are detailed in Additional file 1.

### Neuropeptide discovery and expression

#### *Carma_*CNS-transcriptome

Of the 52 neuropeptide families searched for, representatives of 47 were discovered: 104 transcripts (from 87 clusters) were identified, predicted to encode for 59 full-length peptides or proteins (comprising a ‘start’ methionine, a signal peptide, and a stop codon), 24 C-terminal partial proteins (6 of which contained partial signal peptides), 13 N-terminal partial proteins and 8 internal (lacking both termini) fragments (see Additional files 2 and 3). Analysis of post-translational processing of these conceptual proteins predicts 160 unique mature peptides and 185 precursor-related peptides (PRPs) (see Additional file 3). This is likely an under-estimate of the total number of mature peptides encoded in this transcriptome as prepro-hormones of some neuropeptides contain multiple copies of identical mature peptides (e.g. a HIGSLYamide C-terminal partial protein contained 6 identical mature peptides; see Additional file 3). For *Daphnia pulex*, 43 genes encoding 73 neuropeptides were predicted in silico; mass spectrometry confirmed 40 of these by mass matching and 30 by fragmentation sequencing [62]. Within the *Drosophila* genome, some 42 genes encode neuropeptide precursors predicted to produce approximately 75 mature peptides although the translation of many of these transcripts has not been confirmed in vivo [63]. Comparatively, the number of neuropeptide precursors identified, and mature peptides predicted here for *C. maenas* appears large. However, recent efforts mining decapod transcriptomes have yielded similarly large numbers of transcripts coding for neuropeptide precursors and mature peptides. For example, Christie (2016) identified transcripts from 29 neuropeptide families predicted to produce approximately 112 mature peptides (in addition to 151 PRPs) for *C. maenas* [26]. Furthermore, Veenstra (2015) identified 58 transcripts encoding neuropeptides from some 41 families for the crayfish *Procambarus clarkii* [51] and in a substantial transcriptome mining effort, Veenstra (2016) identified 66 ‘genes’ from 47 neuropeptide families for *C. maenas* as well as comparable values for *Scylla paramamosain* (59 ‘genes’, 45 neuropeptide families), *Eriocheir sinensis* (68, 49), *Litopenaeus vannamei* (70, 49), *Macrobrachium rosenbergii* (67, 48), *Homarus americanus* (59, 45), and *Procambarus clarkii* (63, 49) [27]. Mining efforts for chelicerate transcriptomes and genomes have yielded yet greater numbers of neuropeptide precursors, a result of paralog genes from genome duplications: e.g. 87 and 79 neuropeptide precursors for the spider *Stegodyphus mimosarum* and the scorpion *Mesobuthus martensii*, respectively [64].

For *C. maenas*, the production of 122 mature peptides within the CNS and neuro-endocrine organs has been confirmed by mass spectrometry [65]. Numerous mature peptides predicted in silico here and elsewhere for *C. maenas*, or confirmed biochemically, are identical or very similar isoforms [26, 65]. Whether such isoforms are physiologically redundant or serve distinct functions is undetermined [63]. For example, the 25 mature allatostatin-As detected in the *C. maenas* CNS by mass spectrometry comprise the characteristic YXFGLamide C-termini but, differ in N-termini sequence [65]. Here, a single putative allatostatin-A GPCR was discovered (see below, Table 2), suggesting that the 25 or so isoforms may indeed be physiologically redundant, although this has yet to be functionally determined. In this context, it is interesting to note that there is evidence for neuropeptide redundancy in *Drosophila* [66]. Of course, we should not ignore an alternative scenario that further allatostatin receptors are present but were not predicted in the current analysis. As for all predicted GPCR and their putative ligands, functional de-orphanisation is the only way to clarify the actual situation.

Representatives from allatotropin, androgenic gland specific insulin-like peptide (IAG), DENamide, DXXRLamide, and FXGGXamide families were absent from the *Carma_*CNS-transcriptome. The discovery of DENamides is so far limited to the cladoceran water flea, *D. pulex* [62] whilst DXXRLamides and FXGGXamides have been identified only in copepods [67]. Like DENamide, allatotropin is present in *D. pulex* and the copepods *Tigriopus californicus* and *Lepeophtheirus salmonis* and putative allatotropins are present in the transcriptomes of the amphipods, *Talitrus saltator* (NCBI: PRJNA297565) and *Hyalella azteca* (NCBI: XM_018171664.1; see Additional file 1: Figure S5). Given that allatotropin is present in molluscs, annelids, and chelicerates, it is presumably an ancient neuropeptide which may have been lost in decapod crustaceans [27]. Androgenic gland specific insulin-like hormone has been identified in a number of decapods, but the same study failed to find it in *C. maenas* [27]. In studies reported to date [68] IAG was found only in the androgenic gland and it is not therefore, surprising that we did not sequence its transcript in *C. maenas* and its existence in this species should not be ruled out. We were able to identify a transcript for CHH-MIH-like neuropeptide (the function of which is unknown) previously described in other decapods, but until now absent from *C. maenas* [27]. The diversity of neuropeptides discovered here from the *Carma_*CNS-transcriptome is consistent with other studies on *C. maenas* and comparable with other decapods [26, 27, 65].

Of the 87 neuropeptide contig clusters identified, 9 were excluded from differential gene expression analysis because of very low read numbers (< 1 read per million in > 3 samples) [45]. Our analysis revealed that only 5 neuropeptides were differentially expressed across the moult cycle: carcikinin/ecdysis triggering hormone (ETH), crustacean hyperglycemic hormone-2 (CHH-2), crustacean female sex hormone (CFSH), crustacean cardioactive peptide (CCAP), and neuroparsin-3 (*P* < 0.05, FDR < 0.05; Table 1).

**Table 1:** Neuropeptide transcript differential expression statistics

Neuropeptide	Cluster	LogFC				F	*P*	FDR
		A-B	D1	D3	D4			
Carcikinin/ETH	Cluster-5052.1	1.18	2.31	4.4	5.9	47.91	5.42E-11	2.63E-06
CHH-2	Cluster-569.33175	2.76	0.04	1.6	3.21	7.14	0.001	0.023
Neuroparsin-3	Cluster-141,623.0	−1.27	1.68	−0.32	0.14	7.03	0.001	0.024
CFSH	Cluster-126,814.0	3.74	5.66	4.19	0.72	6.4	0.001	0.034
CCAP	Cluster-569.9440	−0.62	−0.93	− 0.32	0.22	6.66	0.002	0.041
Bursicon-α	Cluster-129,891.0	−0.57	−1.17	− 0.16	0.3	5.36	0.003	0.062
Bursicon-β	Cluster-121,462.0	−0.54	−1.1	− 0.07	0.21	5.02	0.005	0.076
Allatostatin-CC	Cluster-154,370.0	−0.35	− 0.9	0.02	0.22	5.52	0.008	0.112

Differential gene expression analysis statistics for five *C. maenas* neuropeptides which were differentially expressed across the moult cycle (*P* < 0.05 and FDR < 0.05, bold font above line) and three non-differentially expressed neuropeptides (*P* < 0.05 but FDR > 0.05, below line) within the CNS. LogFC are relative to moult stage C3–4. The *P*-value should be read in conjunction with the false discovery rate (FDR)

### Carcikinin/ecdysis triggering hormone (ETH)

**Fig. 1:**
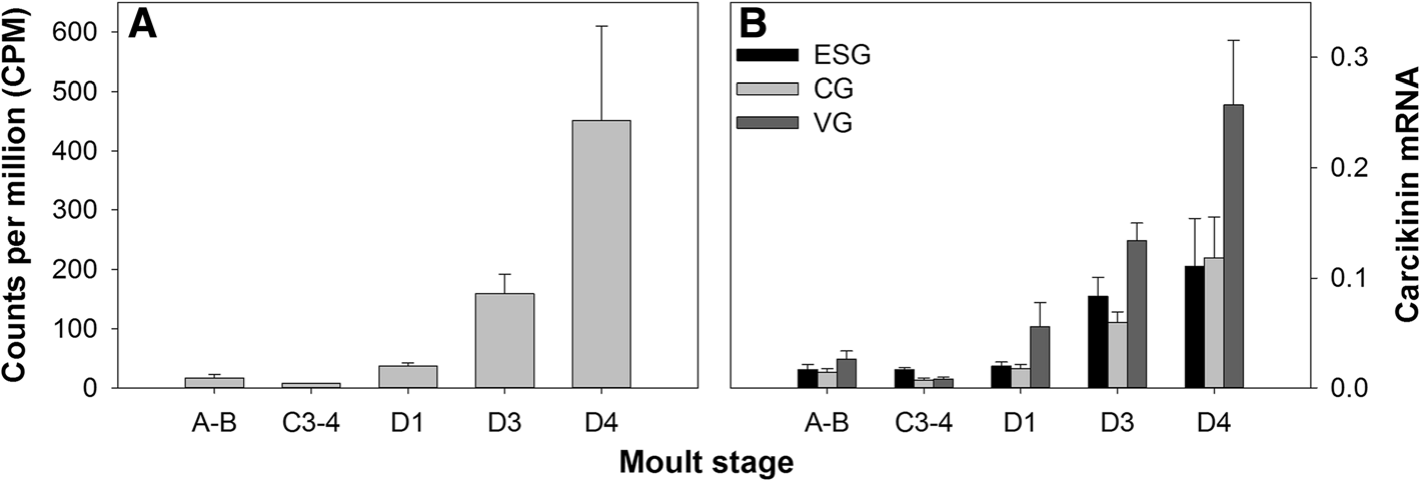
Carcikinin/ETH gene expression across the moult cycle within the central nervous system of *C. maenas*, determined by **A** RNAseq and **B** qPCR. For qPCR data, carcikinin/ETH mRNA copy numbers are normalized to the geometric mean of the reference genes *elongation factor 1-alpha* and *ubiquitin-conjugating enzyme* E2 L3 (data are expressed as mean + SEM). In silico data are presented as mean counts per million (CPM, mean + SEM). ESG = eyestalk ganglia, CG = cerebral ganglia, VG = ventral ganglia

The transcripts for a neuropeptide, here assigned the name ‘carcikinin’, exhibited the highest magnitude of differential expression of all clusters across the moult cycle in the *Carma_*CNS-transcriptome (Fig. 1). Three transcripts encoding carcikinin prepro-hormones were identified: two were full prepro-hormone transcripts and one was an N-terminal partial sequence with an N-terminal extension upstream of the signal peptide (Additional file 1: Figure S6; see also Additional files 2 and 3). Such N-terminal extensions were also observed for other prepro-hormones identified here (and that have been reported in other in silico mining studies) though whether these are assembly artefacts or have some biological function is yet undetermined [26]. Of the two deduced full-length precursor peptides, one was 136-aa and the other was truncated at 61-aa (Additional file 1: Figure S6). Both precursors have identical 19-aa signal peptides and 16-aa mature peptides (DAGHFFAETPKHLPRIamide) but differ in their precursor-related peptides (PRPs) (Additional file 1: Figure S6; see also Additional file 3).

In silico expression data revealed that carcikinin transcript abundance was low in post-, inter-, and early pre-moult (moult stages A-B, C3–4, and D1, respectively), but increased dramatically during pre-moult stages, D3 and D4 (Fig. 1). Given the importance of ETH to insect ecdysis, coupled with our intriguing in silico data which showed not only that carcikinin was differentially expressed across the moult cycle but that transcript abundance increased strikingly in late pre-moult, we adopted a more targeted quantitative PCR approach to validate in silico data and measure expression of selected genes of interest within defined regions of the CNS. We observed a considerable increase in carcikinin expression during D3 and D4, consistent with in silico data: two-way ANOVA indicated that there was a significant interaction between tissue type and moult stage on carcikinin expression (F_(8, 63_) = 2.218, *P* = 0.0376) with dramatically increased expression in the VG at stages D3 and D4 (Tukey’s post-hoc analysis, *P* < 0.05; Fig. 1B). This expression pattern across the moult cycle strongly suggests a role for VG derived carcikinin in the regulation of ecdysis within *C. maenas* and perhaps decapod crustaceans generally (Fig. 1).

**Fig. 2:**
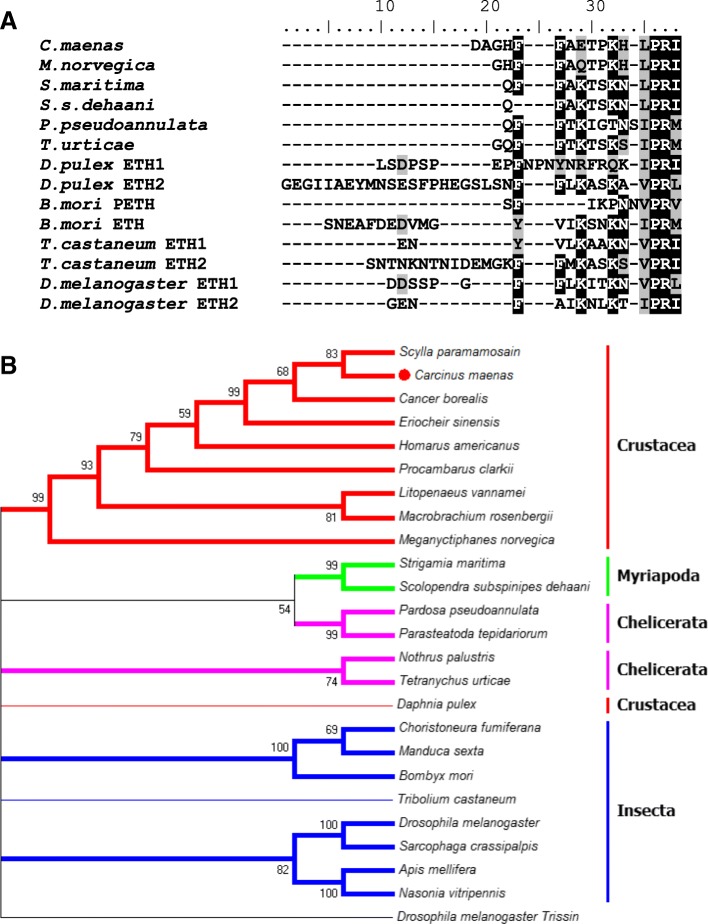
**A** Amino acid alignment for insect pre-ecdysis triggering hormone (PETH), ecdysis triggering hormones (ETH) 1 and 2, and carcikinin, and carcikinin-like peptides from crustaceans, myriapods and chelicerates. Identical residues are highlighted black and conserved substitutions are highlighted grey. **B** Phylogenetic tree of insect ETHs, carcikinin, and carcikinin-like peptides constructed using the Neighbor-joining method. Nodes supported by bootstrap values of < 50% have been collapsed. Protein sequences for *Scylla paramamosain*, *Eriocheir sinensis*, *Homarus americanus*, *Procambarus clarkii*, *Litopenaeus vannamei*, and *Macrobranchium rosenbergii* taken from [27]. Accession codes for sequences taken from the NCBI database: *Cancer borealis*, GEFB01012879.1; *Meganyctiphanes norvegica*, GETT01085899.1; *Scolopendra subspinipes dehaani*, GBIM01000685.1; *Pardosa pseudoannulata*, GCKE01050112.1; *Parasteatoda tepidariorum*, XM_016075645.1; *Nothrus palustris*, GEYJ01054236.1; *Tetranychus urticae*, XM_015935221.1; *Daphnia pulex*, EFX73380.1; *Choristoneura fumiferana*, JX878448.1; *Manduca sexta*, AF165427.1; *Bombyx mori*, NM_001172272.1; *Tribolium castaneum*, XM_008196841.2; *Drosophila melanogaster*, NM_079960.4; *Sarcophaga crassipalpis*, LC018445.1; *Apis mellifera*, NM_001142607.1; *Nasonia vitripennis*, NM_001142635.1; *Drosophila melanogaster* Trissin, NP_650471.1

Carcikinin has previously been identified as a crustacean orthologue of insect ecdysis triggering hormone (ETH), e.g. [27], and a carcikinin-like neuropeptide identified in the genome of the myriapod, *Strigamia maritima*, was assigned the name ETH in that study [50]. In a phylogenetic analysis of insect and *D. pulex* ETHs, crustacean carcikinins, and carcikinin-like peptides mined from the transcriptomes of other arthropods, crustacean carcikinins were clustered in a high confidence branch. A myriapod/chelicerate branch and a separate chelicerate only branch also clustered with high confidence. Insect ETHs were clustered into two high confidence branches, one lepidopteran and the second consisting of dipterans and hymenopterans, which excluded *T. casteneum* (Fig. 2). The branchiopod crustacean, *D. pulex*, was excluded from carcikinin/carcikinin-like peptides and ETH branches. Whether differences in the peptide sequences of insect ETHs and carcikinin/carcikinin-like peptides reflect phylogenetic differences between insects and other arthropods, or indicate that ETH and carcikinins/carcikinin-like peptides are distinct peptides is unclear. *D. pulex* neuropeptides have been found to be more closely related to insect, rather than decapod, homologs and this has been interpreted to confirm that branchiopods are an ancestral group to insects [62]. As such, carcikinin/carcikinin-like peptides may represent a more primitive ETH, common across arthropods. Certainly, amino acid residues within the mature peptide are conserved across arthropods (Fig. 2).

Insect ETH is synthesised and released from peripheral endocrine ‘Inka’ cells located in the trachea and PETH immunochemical localization has revealed peripheral cells in pharate nymphs of the ticks *Ixodes ricinus* and *Rhipicephalus appendiculatus* [69]. Given the peripheral localization and expression of these peptides in insects it is possible that a true crustacean ETH homolog has been missed in the current and previous studies that have harvested material from neural tissues; though it is interesting to note that the only ‘ETH’ identified in the myriapod genome is carcikinin-like (Fig. 2). A fully annotated decapod genome is clearly requisite and would be timely.

### Eclosion hormones (EHs)

Two eclosion hormone (EH)-like ‘genes’ were identified in our *Carma_*CNS-transcriptome (EH-1 and EH-2; see Additional files 2 and 3), consistent with other in silico mining studies of this species and other decapods [26, 27]. The presence of two crustacean EHs is in contrast to insects that invariably have only one. In insects, EH is expressed in ventro-median (Vm) cells of the cerebral ganglion, which project axons through the ventral ganglia and their connectives to proctodeal nerves on the hindgut [7]. As alluded to earlier, in the insect ecdysis cascade EH forms a positive feedback loop with ETH, resulting in the near total release of EH and ETH and signaling the start of ecdysis. ETH in turn initiates pre-ecdysis behaviours whilst EH evokes CCAP and bursicon release, thus committing the animal to ecdysis and terminating pre-ecdysial events [11]. However, recent evidence from *Drosophila* suggests that this view is rather simplistic and that EH may be critical to the initiation of pre-ecdysis behaviours, at least in flies [70].

**Fig. 3:**
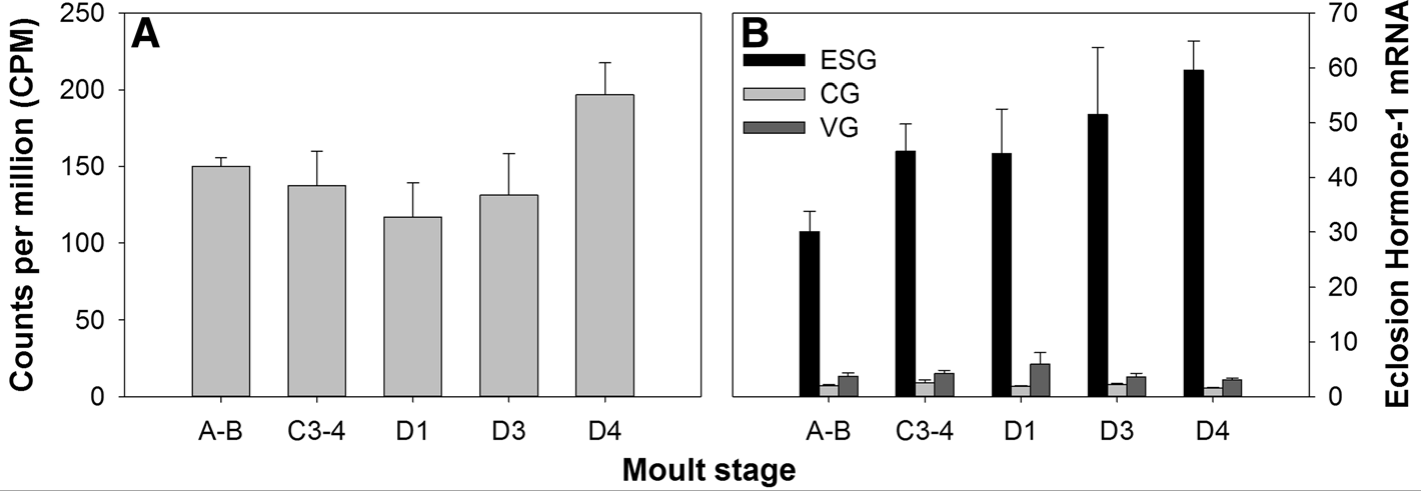
Eclosion hormone-1 gene expression dynamics measured across the moult cycle and within the central nervous system of *C. maenas*, determined by **A** RNAseq and **B** qPCR. For qPCR data, eclosion hormone-1 mRNA copy numbers are normalized to the geometric mean of the reference genes, *elongation factor 1-alpha* and *ubiquitin-conjugating enzyme E2 L3* (data expressed as mean + SEM). In silico data are presented as means counts per million (CPM + SEM). ESG = eyestalk ganlia, CG = cerebral ganglia, VG = ventral ganglia

In the present study, *C. maenas* EH-2 was represented by a single contig encoding a full length protein but was excluded from differential expression analysis because so few reads were counted for the transcript, indicating extremely low expression levels in the CNS. Endpoint PCR of inter-moult animals revealed that EH-2 mRNA is expressed across multiple tissues at low levels (but see below); EH-1 is also expressed across multiple tissues, but at much higher levels (Additional file 1: Figure S7). EH-1 was not differentially expressed across the moult cycle (F = 1.71, *P* = 0.18, FDR = 0.70; Fig. 3), but given the importance of EH in insect ecdysis, we thought it worthy of further investigation. In silico gene expression was validated by qPCR which also revealed the primary location of EH-1 expression within the CNS to be the eyestalk ganglia (ESG; Fig. 3B). Two-way ANOVA to test for the effects of moult stage and tissue type on EH-1 expression showed no interaction effect (F_(8,63)_ = 2.04, *P* = 0.059) but main effects analysis indicated that the mRNA expression in the eyestalk was significantly greater than that in the cerebral and ventral ganglia (F_(2,71)_ = 139.26, *P* < 0.001; Bonferroni post hoc *P* < 0.05) (Fig. 3B). The presence of two EHs, coupled with the expression of EH-1 mRNA predominantly in the eyestalks of *C. maenas* suggests likely differences in the neural architecture of EH producing cells and perhaps EH functioning between insects and crustaceans. Given the fundamental role insect EH plays in insect ecdysis, the localization and functional characterization of crustacean EHs (especially EH-1) and their neural architecture are a priority.

### Crustacean cardioactive peptide (CCAP)

**Fig. 4:**
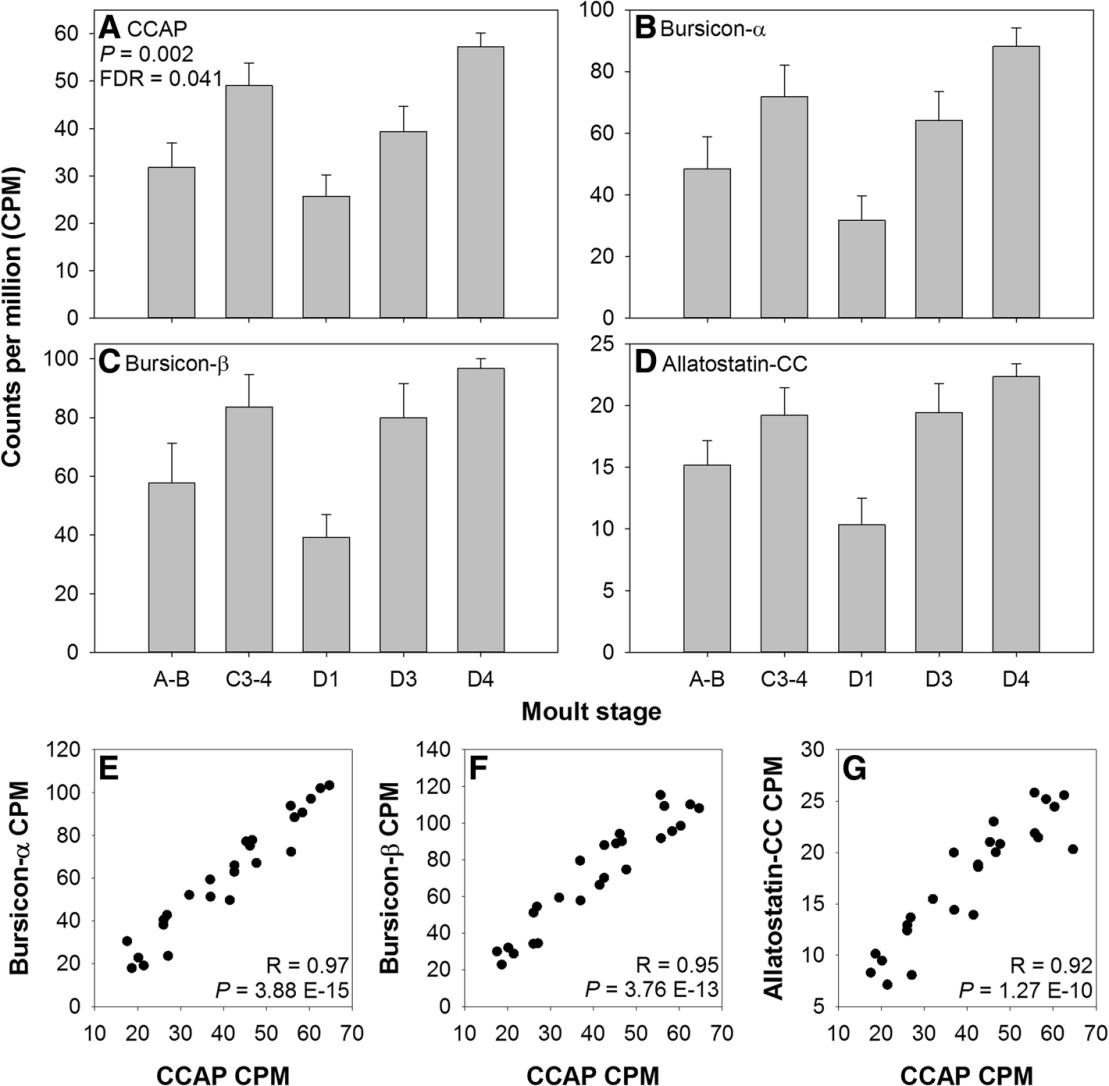
Gene expression profiles across the moult cycle within *C. maenas* central nervous system for: **A** crustacean cardioactive peptide (CCAP), **B** bursicon-α, **C** bursicon-β, and **D** allatostatin-CC, determined by RNAseq. Data are expressed as CPM, mean + SEM. Correlations of gene expression (determined by RNAseq) between CCAP and **E** bursicon-α, **F** bursicon-β, and **G** allatostatin-CC. Pearson’s correlation statistics are shown inset

Three CCAP transcript variants were discovered and assigned to a single cluster, the translated sequences of which were identical in their first 137-aas (and thus produced identical mature nonapeptides: PFCNAFTGCamide) but differed in their C-termini and therefore the CCAP PRPs that they encode (Additional file 1: Figure S8; see also Additional file 3). CCAP PRPs and other neuropeptide PRPs may serve important physiological functions, but to date little evidence for their roles exists. CCAP mRNA was found to be differentially expressed across the moult cycle (*P* < 0.05 and FDR < 0.05; Table 1, Fig. 4) and had a strikingly similar expression profile to those of bursicon-α, bursicon-β, and allatostain-CC_(part a)_, which were constitutively expressed across the moult cycle (*P*-values < 0.05, but FDR > 0.05; Table 1, Fig. 4). Analysis revealed strong positive correlations between CCAP and bursicon-α, −β, and Ast-CC_(part a)_ gene expression (Fig. 4E, F, G). The gene expression profiles observed here for CCAP and bursicon agree with those observed (via qPCR) in other studies of *C. maenas* [71, 72]. Levels of CCAP and buriscon peptides in the pericardial organs of *C. maenas* are closely coupled across the moult cycle whilst levels in the fused ventral ganglion are coupled during inter- and pre-moult, but become uncoupled during ecdysis and post-moult [22].

In insects, CCAP release is stimulated by EH and initiates the ecdysis motor programme whilst halting pre-ecdysis behaviours. Bursicon is co-released with CCAP to initiate the ecdysis motor programme and post-ecdysis cuticle tanning and wing inflation [10, 11]. CCAP and bursicon have been reported to perform similar functions in *C. maenas* [18, 22]. Within *C. maenas*, CCAP and bursicon are co-localised in paired, segmentally repeating perikarya of the ventral ganglion [22, 72]. Given that CCAP and bursicon expression also appears coupled with that of Ast-CC, it would be interesting to explore whether Ast-CC is co-localised in the CCAP-bursicon neurons of the ventral ganglion. In *Drosophila*, CAMB neurons (so called because they produce **C**CAP, **A**llatostatin-CC, **M**yoinhibitory peptide, and **B**ursicon) comprise the ‘master switch’ of ecdysis behaviour following exposure to ETH [73]. Co-localisation of Ast-CC as well as CCAP and bursicon in the paired, segmentally iterated perikarya of the ventral ganglion would indicate that these neurones might be the crustacean equivalent of the fruit fly CAMB neurons and thus, clearly warrant further study.

### Crustacean hyperglycemic hormone (CHH) neuropeptide family

The CHH-superfamily comprises structurally related neuropeptides including type-I peptides: crustacean hyperglycemic hormone (CHH) and insect ion transport peptides (ITP); and type-II peptides: moult inhibiting hormone (MIH), vitellogenesis-inhibiting hormone (VIH), and mandibular organ-inhibiting hormone (MOIH) [19, 73–75]. CHH neuropeptides are involved in numerous physiological events with reported biological activities including regulation of carbohydrate metabolism, osmo and iono-regulation, moulting, gonad maturation, and methyl farnesoate synthesis by the mandibular organ [19]. Here, two CHHs, an MIH, and (for the first time) a CHH-MIH-like neuropeptide transcript were identified (see Additional files 2 and 3). For each of the CHHs (CHH-1 and CHH-2), two transcript variants were assembled, differing from one-another in their C-terminal regions in the translated sequence (Additional file 1: Figure S9). In a study mining decapod transcriptomes, Veenstra (2016) identified two *C. maenas* CHHs, naming them CHH-1 and CHH-2. CHH-1 was already known, having been discovered by traditional methods [76], the second (CHH-2) was newly discovered [27]. Here, we have adhered to the naming of CHHs CHH-1 and CHH-2 according to that of Veenstra (2016) [27]. CHH-1 has been the focus of numerous studies investigating the function of CHH within crustaceans (see below). The conceptual CHH-1s identified here correspond to those sequenced from *C. maenas* pericardial organs (CHH-1a) and the X-organ-sinus gland (CHH-1b) [77] (Additional file 1: Figure S9). The signal peptides and precursor related peptides (CPRP) produced by the two CHH-1 variants correspond to published sequences [27, 77]. To our knowledge, no functional characterisation has been done on CHH-2. Both CHH-2 s have 27-aa signal peptides and 20-aa CHH-precursor-related proteins which have C-terminal amidation signals; however, CHH-2a and b differ in their mature peptides (Additional file 1: Figure S9; see also Additional file 3). Consistent with CHH superfamily peptides, the 83-aa CHH-2a mature peptide has 6 cysteine residues but the 73-aa CHH-2b mature peptide has only 4. The CHH-MIH-like neuropeptide is 128-aa with a 26-aa signal peptide and a 102-aa mature peptide containing 6 cysteine residues (see Additional files 2 and 3).

Three MIH transcript variants were identified from two isoforms, both of which have been reported previously [26]. MIH-a and MIH-b differed from one-another in 9-aa of their signal peptides and 4-aa of their mature peptides (Additional file 1: Figure S10). The second isoform was represented by a C-terminal partial protein (MIH-c; Additional file 1: Figure S10). The cysteines, which form disulfide bridges, are conserved between MIH-a and MIH-b, and the C-terminal fragment of MIH-c (Additional file 1: Figure S10).

**Fig. 5:**
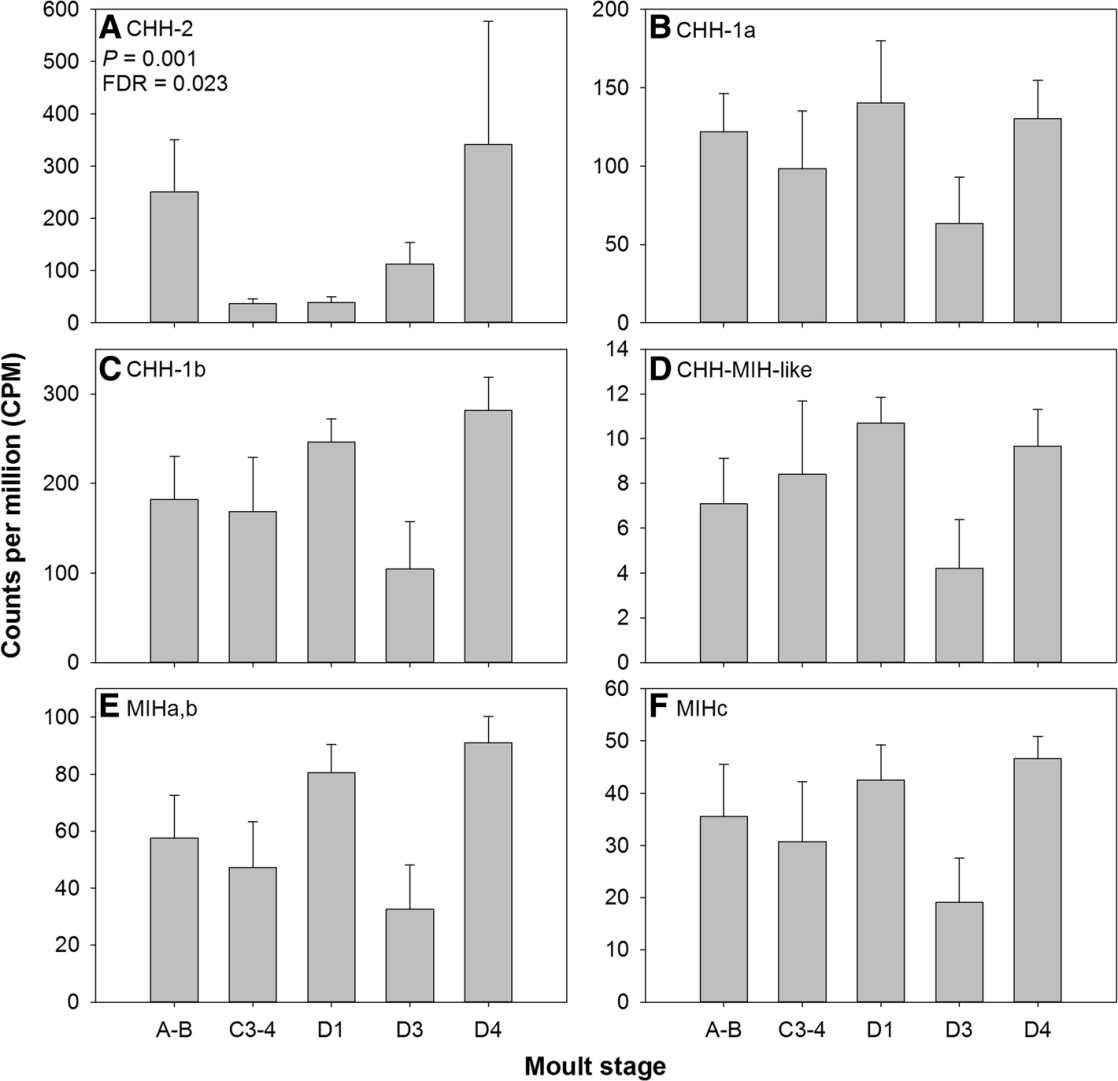
Gene expression profiles of putative CHH-superfamily neuropeptides within *C. maenas* central nervous system across the moult cycle and determine by RNAseq (**A**-CHH-2; **B**-CHH-1a; **C**-CHH-1b; **D**-CHH-MIH-like; **E**-MIHa,b; **F**-MIHc). Only CHH-2 was differentially expressed across the moult cycle within *C. maenas* CNS (statistics values given inset; see also Table 1). Data are presented as mean counts per million (CPM + SEM)

Transcript cluster expression analysis revealed CHH-2 to be differentially expressed across the moult cycle with elevated levels of transcript abundance in moult stages A-B and D4 stages (Fig. 5, Table 1). This expression pattern was strikingly different from other CHH super-family neuropeptides which were constitutively expressed across the moult cycle (Fig. 5). Within *C. maenas*, studies on the function of CHH have concerned CHH-1 only, the two variants of which arise by alternative splicing [19, 20, 77–79]. The defining role of CHH is the mobilization of glycogen resulting in hyperglycemia and increased titres of CHH are observed when crustaceans are exposed to physiologically stressful conditions [19]. An elevation in hemolymph CHH titre is also associated with ecdysis: paraneurons in the fore- and hind-gut discharge CHH, which is known to regulate water and ion uptake during ecdysis [79]. CHH also has an inhibitory activity on ecdysteroid synthesis of the YO, though in *C. maenas* CHH is ~ 10–20 times less potent than MIH [19, 23]. Numerous CHHs have been identified across many decapods species; for example, six were isolated from sinus gland extracts of the kuruma prawn, *Marsupenaeus japonicas*, five of which showed hyperglycemic activity [80]. Similarly, via a recent transcriptome mining effort, two CHHs were reported for *C. maenas* (also identified here: CHH-1 and CHH-2) and the brachyuran crabs *Scylla paramamosain* and *Eriocheir sinensis*, whilst 3 were found in *Macrobrachium rosenbergii* and *Homarus americanus*, and 7 in *Litopenaeus vannamei* [27]. The physiological functions for numerous CHHs have been studied in a variety of decapods and each appear to perform similar roles, although the potency may vary with isoform (for detailed review, see [19]).

In contrast to CHH-2, gene expression patterns of CHH-1a, CHH-1b, CHH-MIH-like, MIH-a,b, MIH-c were similar to each other (Fig. 5). This could reflect different roles for CHH-2 relative to CHH-1 and coupled with differential expression across the moult cycle, with elevated expression during A-B and D4, may suggest a role for CHH-2 in ecdysis regulation. Endpoint PCR revealed that within the CNS, CHH-2 is expressed in the CG and VG, but not the ESG (Additional file 1: Figure S7). Given that the primary site of CHH-1 expression is the X-organ of the ESG, this crude endpoint tissue distribution has identified likely differences in the neural architecture of CHH-1 and CHH-2 synthesising perikarya. Given the differing expression dynamics identified here between CHH-2 and other CHH-superfamily peptides (CHH-1/CHH-MIH-like/MIH), coupled with likely differences in peptide synthesis location, studies of physiological functions of newly identified CHHs (CHH-like neuropeptides) may find diverse roles that differ from those established for CHH.

### Crustacean female sex hormone (CFSH) and Neuroparsin-3

The recently discovered CFSH is implicated in the development of female reproductive characteristics [81]. We identified transcripts coding for CFSH and two CFSH-like neuropeptides in the *Carma_*CNS-transcriptome (Additional file 1: Figure S11; see also Additional files 2 and 3), the first time the deduced sequences of the CFSH-like neuropeptides have been reported for this species [27]. The deduced *C. maenas* CFSH prepro-hormone is a 225-aa peptide with a 24-aa signal peptide. It is cleaved at a KR dibasic cleavage site to produce a 32-aa CFSH-PRP and a 166-aa mature CFSH, which has 8 cysteine residues predicted to form 4 disulfide bridges (C_46_-C_150_, C_80_-C_112_, C_105_-C_119_, C_107_-C_148_) consistent with that reported for *Callinectes sapidus* CFSH [81]. CFSH-like 1 prepro-hormone is a 297-aa protein with a 22-aa signal peptide, a 102-aa CFSH-like 1 PRP and a 170-aa mature CFSH-like 1 peptide which has 10 cysteine redisues predicted to form 5 disulfide bridges (C_4_-C_53_, C_12_-C_84_, C_107_-C_109_, C_114_-C_123_, C_152_-C_154_). This deduced prepro-hormone also has a 12-aa N-terminal extension immediately upstream the start of its signal peptide (see Additional files 2 and 3). The CFSH-like 2 prepro-hormone codes for a 219-aa protein with a 26-aa signal peptide and a 193-aa mature peptide with 8 cysteine residues predicted to form 4 disulfide bridges (C_73_-C_130_, C_105_-C_128_, C_135_-C_176_, C_143_-C_174_). CFSH-like 2 prepro-hormone does not contain a PRP. CFSH was differentially expressed across the moult cycle (F = 6.40, *P* = 0.001, FDR = 0.034, Table 1); however, expression levels were significantly different between males and females, being expressed at only low levels in males -consistent with previous findings [81]. Two-way ANOVA showed that the effects of moult stage (F_(4,19)_ = 2.958, *P* = 0.047) and sex (F_(1,19)_ = 10.055, *P* = 0.005) on gene expression were significant, the interaction between these factors could not be investigated due to the unbalanced nature of the data. Hence, we must treat our observation that CFSH expression varies across the moult cycle with due caution.

Neuroparsins were first identified as anti-gonadotropic factors which delay vitellogenesis in insects [82–84]. RNA interference experiments confirmed an inhibitory effect of neuroparsin on vitellogenesis and oocyte maturation in female desert locusts, *Schistocerca gregaria* [85]. In contrast, neuroparsin-like ovary ecdysteroidogenic hormones (OEHs) are gonadotropic and ecdysteroidogenic in the mosquito *Aedes aegypti* and ovary maturing parsins (OMPs) of *Locusta migratoria * and *Schistocerca gregaria* similarly stimulate oocyte growth, probably by ovarian ecdysone synthesis stimulation [86–88]. In the shrimp *Metapenaeus ensis*, RNAi pointed to a role for neuroparsin in the maturation of oocytes by stimulating vitellogenin production in the hepatopancreas, similar to the roles of OEH and OMP [89]. Neuroparsins have also been used as markers of phase change in gregarious locusts, being differentially expressed between phases. In *C. maenas* neuroparsin-3 was differentially expressed across the moult cycle, elevated expression was evident in D1 (F = 7.03, *P* = 0.001, FDR = 0.024, Table 1), though this finding is subject to the same caveat as above regarding the unbalanced numbers of males and females per moult stage. Two-way ANOVA was unable to assess interaction because of the unbalanced nature of the data, but indicated that gene expression was affected by moult stage (F_(4,19)_ = 3.931, *P* = 0.017), but not sex (F_(1,19)_ = 0.379, *P* = 0.545).

### *Carma*_YO-transcriptome

**Fig. 6:**
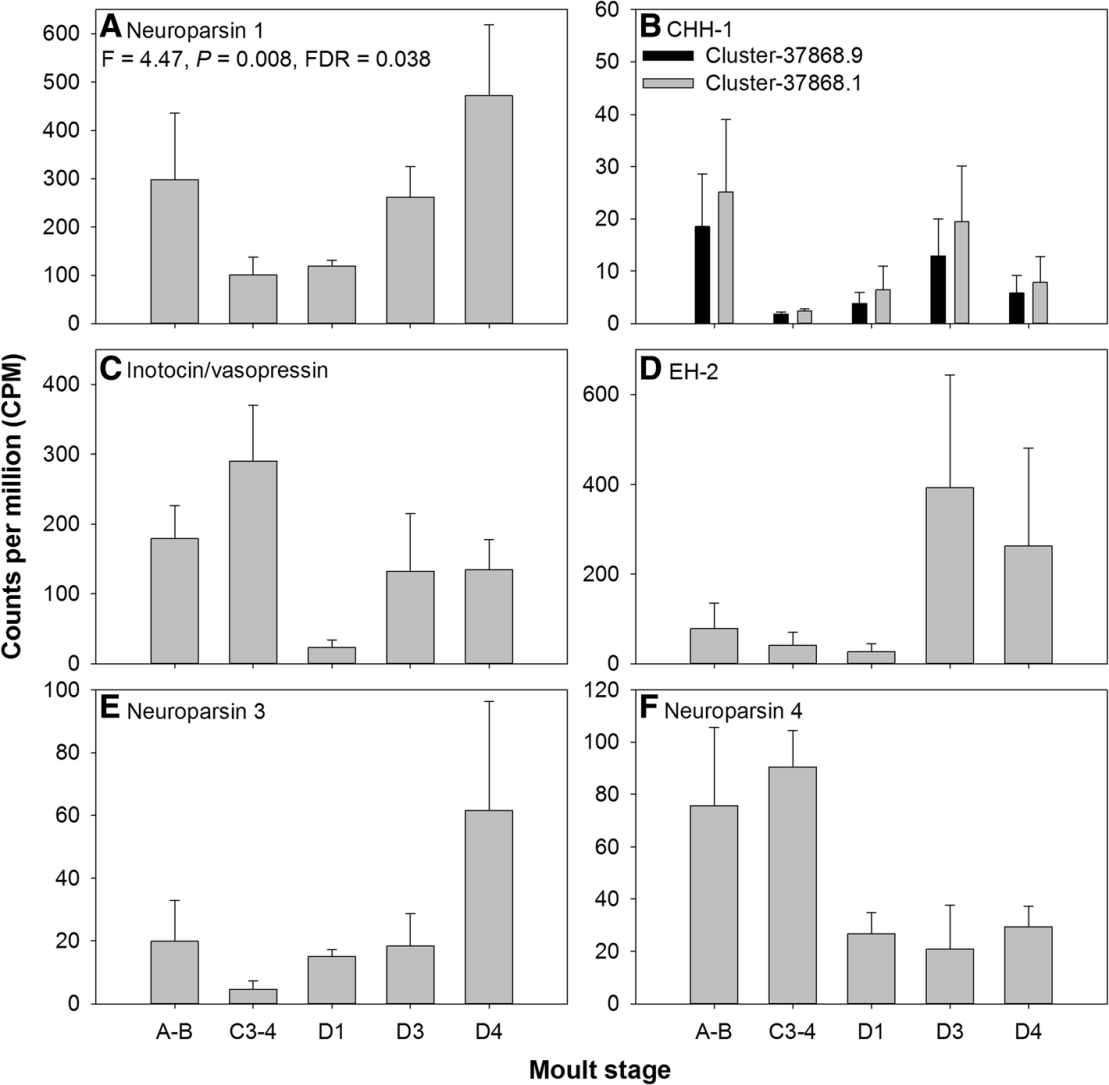
Gene expression dynamics across the moult cycle of six representative neuropeptides within the YO determined by RNAseq (**A**-Neuroparsin; **B**-CHH-1; **C**-Inotocin/vasopressin; **D**-EH2; **E**-Neuoparsin 3; **F**-Neuroparsin 4). Bars show mean counts per million: CPM, mean + SEM. Neuroparsin 1 (A) was found to be differentially expressed across the moult cycle (statistics values given inset). CHH-1 was fragmented and clustered into two clusters, which are included in a single graph (**B**)

The YO is an endocrine tissue, derived from the epidermis and as such expression of neuropeptide transcripts was not expected. To our surprise however, transcripts representative of peptides from across 24 families were identified: 37 transcripts (from 34 clusters) were found to encode 19 full-length peptides or proteins (comprising a ‘start’ methionine, a signal peptide, and a stop codon), 10 C-terminal partial proteins, 5 N-terminal partial proteins and 2 internal (lacking both termini) fragments (see Additional file 2). Six of these neuropeptides were expressed at levels > 10 CPM; including neuroparsin-1, CHH-1, inotocin/vasopressin, EH-2, neuroparsin-3, and neuroparsin-4. Notably, neuroparsin-1 mRNA was differentially expressed within the YO across the moult cycle (Fig. 6). Thus, the YO appears to be a source of synthesis (and perhaps release site) for a number of neuroparsins which, as mentioned previously, are known to play roles in reproductive physiology. Neuroparsin-1 expression was low during inter-moult and early pre-moult (C3–4, D1) but increased through late pre-moult, peaking in D4 and remaining elevated during post-moult (A-B, Fig. 6), therefore marking this YO derived transcript as having a putative role in the ecdysis cassette.

Within the *Carma*_CNS-transcriptome, EH-2 was rarely expressed and end-point PCR using RNA from inter-moult (C4) crabs confirmed low expression across tissues (Additional file 1: Figure S7). However, RNAseq within the YO revealed high expression of EH-2 in pre-moult (D3, D4), though this was not found to be differentially expressed across the moult cycle, presumably due to high variability between biological replicates (Fig. 6). These findings suggest that the two EHs within crustaceans are likely expressed in discrete tissues, perhaps with discrete physiological functions.

### Putative neuropeptide receptor discovery and expression

#### *Carma*_CNS-transcriptome

**Fig. 7:**
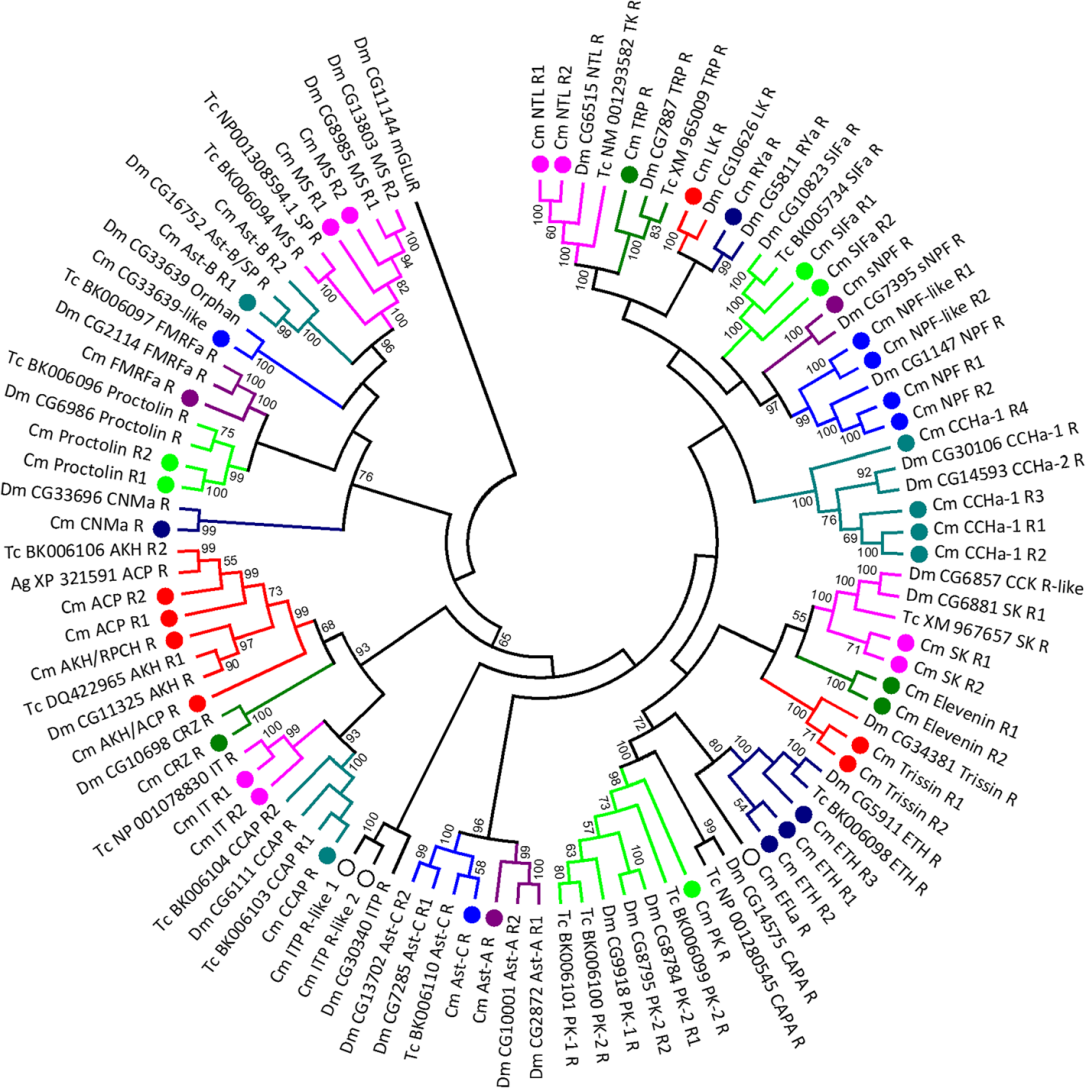
Rhodopsin-like (class A) putative neuropeptide G protein-coupled phylogenetic tree constructed for *C. maenas* (*Cm*), *Drosophila melanogaster* (*Dm*), *Tribolium castaneum* (*Tc*), and *Anopheles gambiae* (*Ag*). NCBI accession codes and *Drosophila* gene codes are detailed in the figure

**Fig. 8:**
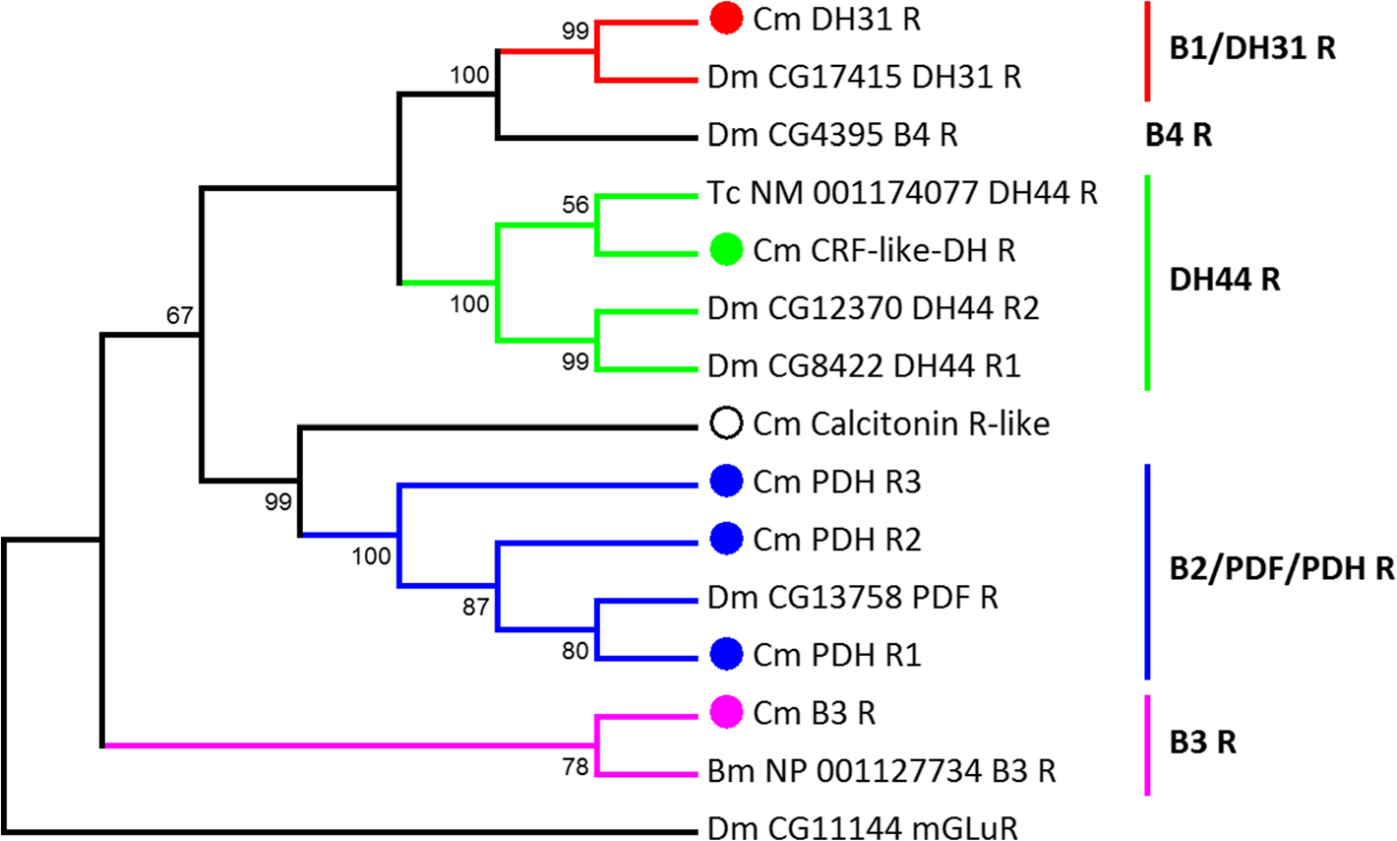
Secretin family (class B) putative neuropeptide G protein-coupled receptor phylogenetic tree constructed for *C. maenas* (Cm), *Drosophila melanogaster* (Dm), *Tribolium castaneum* (Tc), *Bombyx mori* (Bm). NCBI accession codes and *Drosophila* gene codes are detailed in the figure

**Fig. 9:**
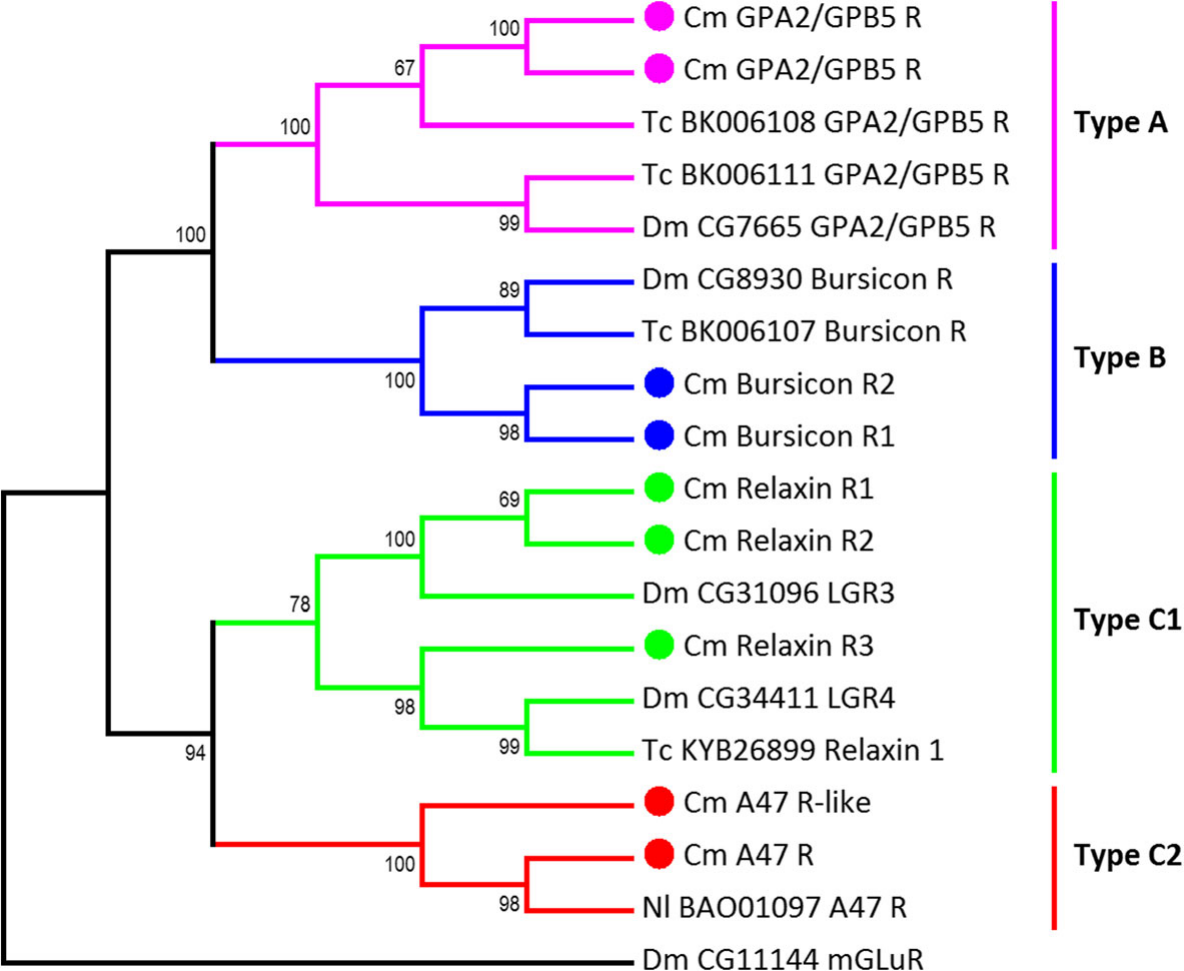
Leucine-rich repeat-containing (LGR) putative neuropeptide G protein-coupled receptor phylogenetic tree constructed for *C. maenas* (*Cm*), *Drosophila melanogaster* (*Dm*), *Tribolium castaneum* (*Tc*), and *Nilaparvata lugens* (*Nl*). NCBI accession codes and *Drosophila* gene codes are detailed in the figure

A total of 66 putative neuropeptide GPCRs were discovered within the *C. maenas* CNS: 100 transcripts (from 72 clusters) were identified, encoding 77 full-length proteins, 13 C-terminal partial proteins, nine N-terminal partial proteins, and one likely internal fragment (see Additional file 2). Of the 77 full-length protein coding sequences discovered, 63 were predicted to contain 7 transmembrane helixes (predicted by tmhmm). In addition to these putative GPCRs, one insulin receptor tyrosine kinase (de-orphanised as a receptor for IAG [90] and, since it is found in both sexes, likely also a receptor for other insulin-like ligands [91]) three insulin receptor-like tyrosine kinases, and two venus kinase receptors (recently deorphanised in the mosquito *Aedes aegypti* as neuroparsin receptors [92]) were identified (see Additional file 2). Within the *Drosophila* genome, 49 neuropeptide GPCRs are present, 35 of which have been functionally characterised [93]. The highly conserved nature of arthropod (and particularly insect and crustacean) neuropeptidomes enabled us to predict putative *C. maenas* neuropeptide receptors based on sequence similarity to those de-orphanised in insects [27, 64]. On this basis, the 66 putative *C. maenas* neuropeptide GPCRs have been assigned putative ligands from 33 neuropeptide families; six *C. maenas* GPCRs are homologous to orphan insect GPCRs and thus, at present, cannot be assigned a ligand: neuropeptide B3 R, CG33639-like, MOODY, MOODY-like, neuropeptide A47 R, and neuropeptide A47 R-like. Of the 66 putative neuropeptide receptors, 50 were class A (rhodopsin-like), seven were class B (secretin-like), and nine were leucine-rich repeat-containing GPCRs (LGR) (Figs. 7, 8, 9) (see also Additional file 2). Of the neuropeptides present with characterized GPCRs, only agatoxin-like peptide and periviscerokinin lacked putative GPCRs. Owing to the lack of characterized receptors for CCRFamide, CFSH, HIGSLYamide, Hyrg, and Orcokinin, no putative receptors for these were identified in the present study. Red pigment concentrating hormone is related to adipokinetic hormone and is the putative ligand of an AKH receptor; one of which was discovered in the *Carma_*CNS-transcriptome as well as an AKH/ACP-like receptor, which has similarities to both AKH and ACP receptors. The AKH receptor identified here (AKH/RPCH R Fig. 7) was subsequently de-orphanised  and confirmed as the receptor of the native RPCH neuropeptide [89].

G protein-coupled receptors for ion transport peptide (ITP), which are part of the CHH superfamily (type 1 CHHs, see [19]), and ITP-like peptides have been de-orphanised in the silkworm *Bombyx mori* [94] In the present study two ITPR-like GPCRs were discovered (Fig. 7, Additional file 2). Since these ligand-receptor pairs are likely to be highly conserved, and given that ITPR-like GPCRs have been identified in the decapods [32, 51] and may be broadly represented across crustaceans, this is a potentially very important avenue that must be pursued further in an effort to establish a functional CHH receptor in *C. maenas.*

The number of putative neuropeptide GPCRs identified for *C. maenas* exceeds that determined from insect genomes for *Drosophila* (49), *Tribolium castaneum* (48), and *Bombyx mori* (39) and from insect transcriptomes for *Nilaparvata lugens* (57) and *Chilio suppressalis* (51) [93, 95–98], but is fewer than that determined from chelicerate genomes for *Stegodyphus mimosarum* (120) and *Mesobuthus martensii* (93) [64]. The relatively large number of neuropeptide GPCRs in chelicerates, reflected also in the number of peptide ligands, results from gene paralogs that have been maintained in most extant chelicerates after ancient genome duplication events [64]. Phylogenetic analysis of putative neuropeptide GPCRs supports the notion that there are paralogous receptor genes for a number of neuropeptide receptors within *C. maenas* (Figs. 7, 8, 9, Table 2). Four putative CCHa receptors were discovered which cluster with the *Drosophila* CCHa-1 and -2 receptors (CG30106 and CG14593, respectively: Fig. 7). Two neuropeptide F receptors (NPFR) and two NFPR-like receptors cluster with the *Drosophila* NPFR, CG1147. Finally, three putative ecdysis triggering hormone receptors (ETHR) were discovered and that cluster with *Drosophila* ETHR (CG5911) and *T. castaneum* ETHR (Fig. 7). These are examples of multiple neuropeptide gene paralogs identified in *C. maenas*, but there are numerous others where two paralogous receptor sequences have been found in the *Carma*_CNS-transcriptome but which occur as a single receptor gene in *Drosophila*; for instance, the natalisin receptor, SIFa receptor, sulfakinin receptor, trissin receptor, and proctolin receptor (Fig. 7). A summary of the deduced number of neuropeptides and putative neuropeptide receptor genes is detailed in Table 2. The high confidence clustering of putative neuropeptide receptors identified here for *C. maenas* with those  de-orphanised in *Drosophila* (and *T. castaneum* etc.) is strongly suggestive of the functional identity of the putative ligands (as we have done). However, the sequence similarities between GPCRs means that such an approach has potential to be erroneous and so functional de-orphanisation strategies are essential in the identification of GPCR transcripts with their ligands [93].

**Table 2:** Neuropeptide ligand (L) and putative G protein-coupled receptor (R) genes identified within the *Carma*_CNS-transcriptome compared with data for selected arthropods

	*C. maenas*		*H. americanus *[29]		*P. clarkii* [51]		*S. maritima *[50]		*Drosophila *[93]	
	L	R	L	R	L	R	L	R	L	R
ACP	1	2^a^	1	1	1	1	1	1		
AKH\RPCH	1	1^a^	nf	2	1	1	1	1	1	1
Agatoxin-like peptide (ALP)	1	nf								
Allatostatin-A	1	1	1	1	1	1	1	1	1	2
Allatostatin-B\MIP	1	2			1	1	1	1	1	1
Allatostatin-C\CC\CCC	1\2\1	1	2	3	2\1\nf	3	1\1\nf	nf	1	2
Allatotropin							1	1		
Bursicon-a\b	1\1	2	1\1	2	1\1	2	1\1	1	1\1	1
Calcitonin	1	1			1	nf				
CAPA									1	1
CCHamide	2	4	2	2	2	2	2	1	2	2
CCRFamide	1	?			1	n?				
CNMamide	1	1			1	1				
Corazonin	1	1	1	1	1	1	1	2	1	1
CRF-like DH (DH44)	1	1	1	2	1	1	1	1	1	2
CCAP	1	1	1	1	1	nf	1	1	1	1
CFSH	1	?			1	?				
CFSH-like	2	?			2	?				
CHH	2	?	4	?	2	?				
CHH-MIH-like	1	?			2	?				
DH31	1	1	1	3	1	1	1	1	1	1
EH	2	2	2	nf	2	2	2	2		
Carcikinin\ETH	1	3	nf	3	nf	2	1	2	1	1
EFLamide	1	1			1	nf	1	nf		
Elevenin-like peptide	1	2			1	nf	1	nf		
FMRFamide	1	1	1	1	1	1	2	1	1	1
GPA2\GPB5	1\1	2			1\1	1			1\1	1
HIGSLYRamide	5	?								
Hyrg	1	?								
ILP	1	1	1	2	1	1	1	nf		
Intocin	1	2	1	nf	1	2	1	2		
ITP	nf	2					1	nf		
Leucokinin	1	1	2	1	1	1			1	1
MIH	1	?			1	?				
Myosuppressin	1	2	1	1	1	2	1	2	1	2
Natalisin	1	2			1	1			1	1
Neuroparsin	4	2	1	nf	3	2				
NPF	2	2^a^	1	4	2	3	1	1	1	1
Orcokinin	2	?	1	?	1	?				
Periviscerokinin	1	nf			1	1				
PDH\ePDH	3\1	3	1	2	3	4			1	1
Proctolin	1	2	1	2	1	2	1	1	1	1
Pyrokinin	1	1	2	1	1	1	1	2	2	3
Relaxin-like	2	3			1	nf				
RYamide	1	1			1	1	1	1	1	1
short Neuropeptide F	1	1	nf	1	1	1			1	1
SIFamide	1	2	1	1	1	2	1	2	1	1
Sulfakinin	2	2	1	1	1	2	1	3	1	1
TRP	1	1	1	3	1	2	1	1	1	2
Trissin	1	2			1	nf			1	2

Data from *H. americanus* [29], *P. clarkii* [51], *S. maratima* [50], and *Drosophila* [93]. For *C. maenas,* ^a^ = additional receptors with sequence similarity identified: 1× ACP/AKH R, 2× NPF-like R. nf = not found,? = receptor not known. See also Table 1 of Veenstra 2016 for a similar comparison of neuropeptide ligand and receptor genes for numerous arthropod species including arachnids [64]

**Fig. 10:**
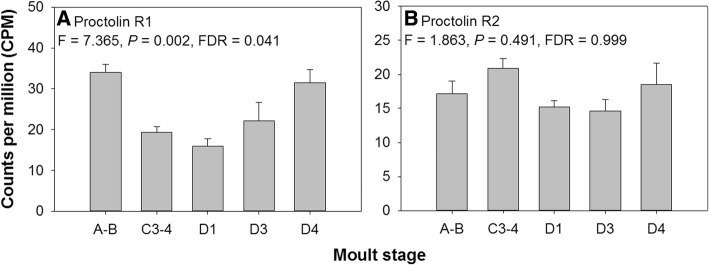
Putative proctolin receptor 1 (panel **A**) and 2 (panel **B**) gene expression across the moult cycle within *C. maenas* central nervous system, determined by RNAseq (data expressed as counts per million, CPM + SEM)

Only one of the putative neuropeptide receptors discovered was differentially expressed at the transcript level across the moult cycle: proctolin R1 (Fig. 10). Two potential proctolin receptors were discovered (as is the case for other decapods crustaceans, see Table 2 [29, 51]); gene expression analysis showed that R2 was constitutively expressed across the moult cycle whilst R1 was more highly expressed during pre- and post-moult, in stages D4 and A-B, relative to early inter-moult and early pre-moult, D1 (Fig. 10). A single neuropeptide proctolin is present in the *Carma_*CNS-transcriptome whilst there are two putative receptors (Table 2). The presence of two paralogs, coupled with the differential expression of one across the moult cycle, suggests that the receptors may have specialized roles and that proctolin R1 may be important during the moult cycle. Proctolin is a powerful stimulant of muscle contractions. For instance in *Drosophila*, proctolin induced sustained muscle contractions in third instar larvae with the CNS removed whilst diminished proctolin receptor expression leads to reduced crawling velocity at high temperatures [99]. In crustaceans, proctolin has been isolated from the pericardial organs and can affect muscle contractions of heart and skeletal muscles and modulate pyloric rhythms [100–102]. These reports align with the necessity for stereotyped muscle contractions evident during ecdysis as the animal extricates the old cuticle, and in light of the putative proctolin receptor expression dynamics it is tempting to speculate that this peptide and receptor pair plays a role in this regard. It will be intriguing to investigate tissue specific expression of the two putative proctolin receptors.

Annotation of the *Carma_*CNS-transcriptome resulted in 221 clusters with the GO:0004930 ‘G protein-coupled receptor activity’. Of these 221 clusters, eight were found to be differentially expressed across the moult cycle (one of which was proctolin R1: annotated as ‘FMRFamide receptor-like’, Cluster-569.17428; Additional file 1: Table S6 and Figure S12). All except for Cluster-569.40579 (annotated as Fizzled-2-like) were elevated during post- and pre-moult relative to inter-moult (Additional file 1: Figure S12). With the exception of proctolin R1 (Cluster-569-17,428), no other GPCRs showed high sequence similarity to known neuropeptide receptors. Given that the moult cycle and the process of ecdysis pervade all levels of crustacean physiology, it is perhaps unsurprising that non-neuropeptide GPCRs are differentially expressed across the moult cycle.

#### *Carma_*YO-transcriptome

**Fig. 11:**
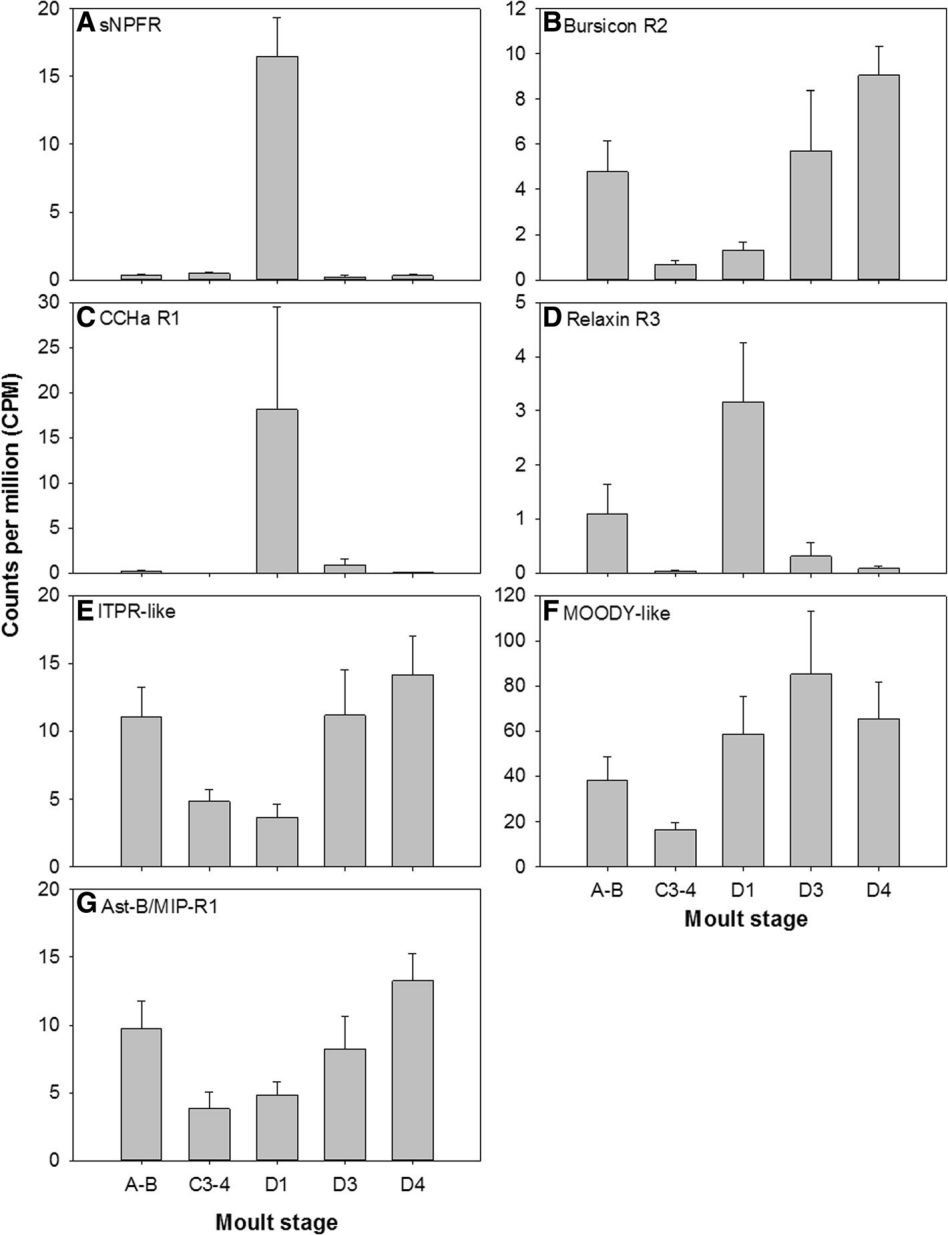
Gene expression patterns across the moult cycle within the YO for differentially expressed putative neuropeptide receptors (see Table 3 for statistics), determined by RNAseq (**A**-sNPFR; **B**-Bursicon R2; **C**-CCHa R1; **D**-Relaxin R3; **E**-ITPR-like; **F**-MOODY-like; **G**-Ast-B/MIP-R1). Data are expressed as mean counts per million, CPM + SEM)

Thirty putative neuropeptide GPCRs were discovered within the *Carma_*YO-transcriptome: 43 transcripts (from 35 clusters) were identified encoding 24 full-length proteins, seven C-terminal partial proteins, five N-terminal partial proteins, and seven internal fragments (see Additional file 2). Of these, 25 were predicted to contain seven transmembrane helices (predicted by tmhmm). In addition to GPCRs, two venus kinase receptors were identified. Of the 30 putative neuropeptide GPCRs, 20 were rhodopsin-like, four were secretin-like, and six were LGR type and were assigned putative ligands from 24 neuropeptide families based on sequence similarity to de-orphanised neuropeptide GPCRs (see Additional file 2). Transcripts for the putative neuropeptide receptors allatostatin-A R, CRZR, CCAPR, short neuropeptide F receptor (sNPFR), and bursicon R2, encoded full-length proteins in the YO, whilst those identified from the *Carma*_CNS-transcriptome were incomplete. In addition, whilst a single calcitonin-like-DH-31 R was identified from the *Carma_*CNS-transcriptome, an additional three transcript variants were identified in the *Carma_*YO-transcriptome (see Additional file 2). Of the putative neuropeptide receptors identified in the YO, seven were differentially expressed across the moult cycle (Table 3, Fig. 11). sNPFR was the most differentially expressed cluster over the moult cycle within the *Carma*_YO-transcriptome, with low expression levels throughout most of the moult cycle, but was upregulated during early pre-moult, D1 (Table 3, Fig. 11A).

**Table 3:** Putative neuropeptide  G protein-coupled receptor differential expression statistics

Putative neuropeptide GPCR	Cluster	LogFC				F	*P*	FDR
		A-B	D1	D3	D4			
Short neuropeptide F R	Cluster-4928.1	−0.48	5.09	−0.90	−0.56	50.25	2.45E-11	1.24E-06
Bursicon R2	Cluster-136,339.5130	2.78	0.90	3.04	3.70	9.81	7.49E-05	0.002
CCHa R1	Cluster-136,339.27034	4.93	11.30	7.01	3.68	10.80	7.82E-05	0.002
Relaxin R3	Cluster-136,999.0	4.38	5.89	2.70	0.85	9.70	8.10E-05	0.002
ITPR-like	Cluster-133,078.1	1.18	−0.40	1.20	1.55	5.84	0.002	0.016
MOODY-like	Cluster-136,339.8771	1.22	1.84	2.37	2.00	5.07	0.004	0.025
Ast-B/MIP-R1	Cluster-136,339.8223	1.34	0.34	1.10	1.79	4.59	0.011	0.049

Differential gene expression analysis statistics (F, *P*, FDR) for seven *C. maenas* putative neuropeptide G protein-coupled receptors which were differentially expressed across the moult cycle within the Y organ. LogFC are relative to C3–4. The *P*-value should be read in conjunction with the false discovery rate (FDR)

Annotation of the *Carma_*YO-transcriptome annotated 160 clusters with ‘GO:0004930 G protein-coupled receptor activity’. Of these, 18 were found to be differentially expressed across the moult cycle (Additional file 1: Table S9 and Figure S13), one of which was sNPFR and another was allatostatin-B/myoinhibitory peptide R1. Of the remaining 16 GPCRs, which did not have high sequence similarity to known neuropeptide receptors, 12 contained 7 transmembrane helixes (predicted by tmhmm). Differential expression of GPCRs within the YO, the location of ecdysteroid synthesis, is suggestive of a role for these GPCRs in ecydsis control.

#### *Carma_*YO_vs_Epi-transcriptome

**Fig. 12:**
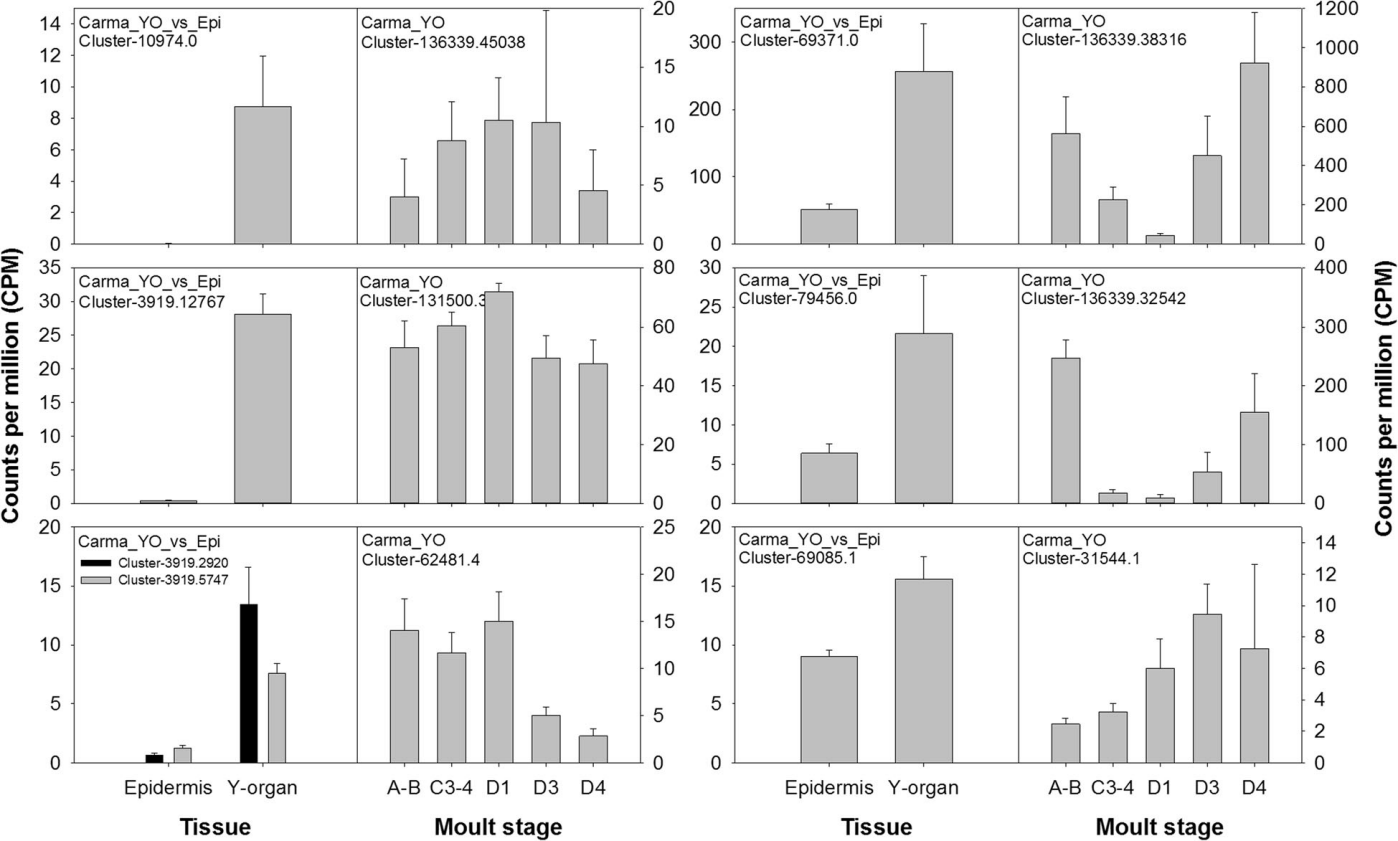
Gene expression profiles of representative clusters annotated ‘GO:0004930  GPCR activity’ and upregulated in YO relative to epidermis of *C. maenas* during inter-moult (C3–4), and across the moult cycle within the YO (*Carma*_YO-transcriptome). See Table 4 for clusters differentially expressed in YO across the moult cycle. Data are expressed as counts per million (CPM + SEM)

**Fig. 13:**
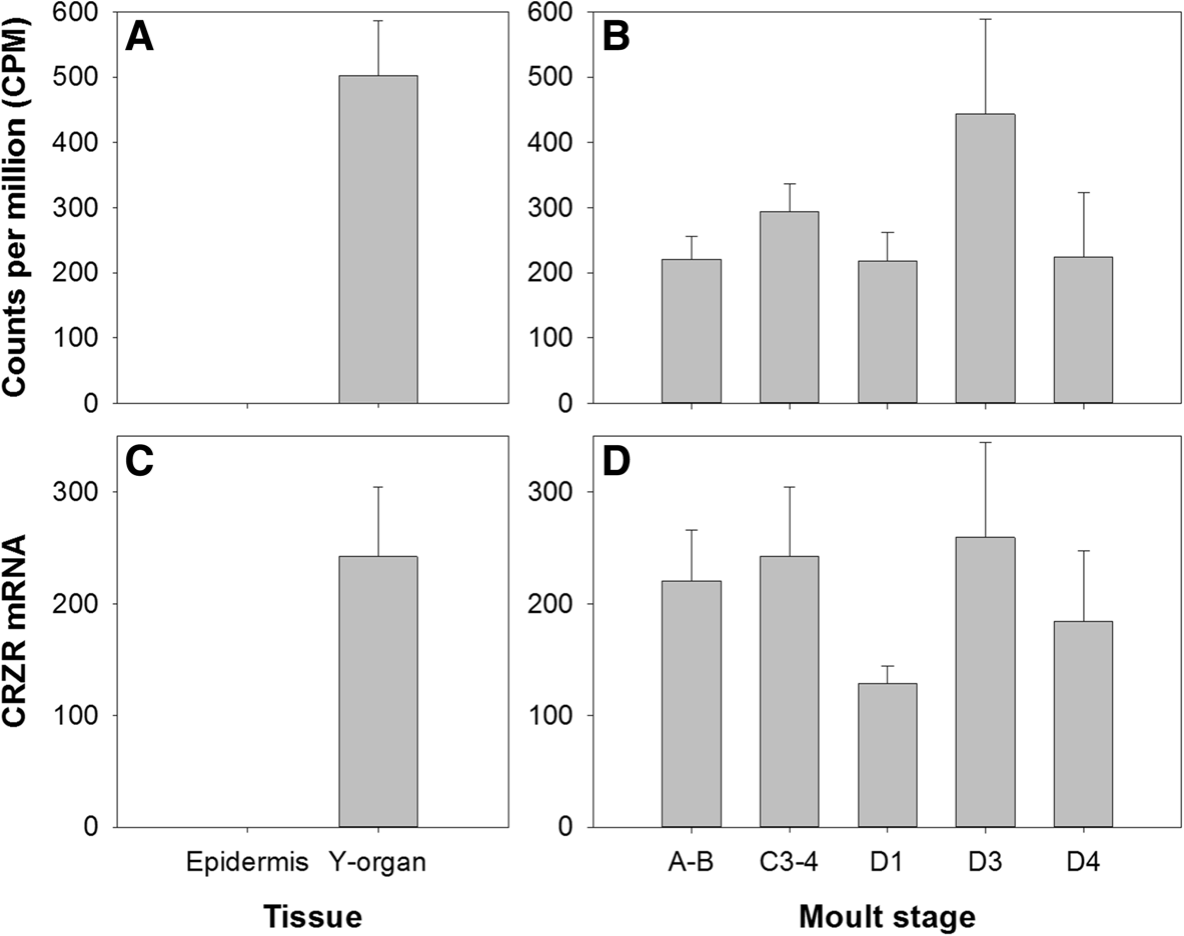
Corazonin receptor (CRZR) gene expression within epidermis and Y-organs (**A**, **C**) and across the moult cycle within the Y-organ (**B**, **D**) of *C. maenas*, determined by RNAseq **A**, **B** and qPCR **C**, **D**. For qPCR data, CRZR mRNA copy numbers are normalized to the geometric mean of the reference genes *elongation factor 1-alpha* and *ubiquitin-conjugating enzyme* E2 L3 (bars show mean values +SEM). In silico data are presented as counts per million (CPM + SEM)

Given that the YO is pivotal in moult control via its central role in ecdysteroid synthesis, we anticipated that elucidation of GPCRs and their expression dynamics within this tissue might yield insight to receptor-ligand pairs critical to moult regulation. Our strategy was to explore differential expression between the YO and epidermis, from which it is derived, to separate transcripts exclusive to the YO. Differential expression of clusters between YO and epidermis tissues during inter-moult (C3–4) identified 162 clusters annotated as GPCRs (‘GO: GO:0004930 G protein-coupled receptor activity’), 11 of which were upregulated in the YO relative to the epidermis (Table 4, Fig. 12) and of these, eight were predicted to contain 7 transmembrane helices (predicted by TMHMM). Three of the GPCRs upregulated in the YO relative to the epidermis were essentially absent from the epidermis, and two were also differentially expressed in the YO across the moult cycle; rhodopsin G0-coupled and probable G protein-coupled receptor Methuselah (Mth)-like 1 (Table 4, Fig. 12). The GPCR cluster that showed most marked differential expression between YO and epidermis tissues was annotated as a gonadotropin-releasing hormone receptor and, according to our phylogenetic analysis of rhodopsin-like GPCRs (Fig. 7), was a candidate for the corazonin receptor (CRZR) (Table 4, Fig. 13). Corazonin initiates the release of ETH from ‘Inka’ cells in *M. sexta*, so the identification of a putative CRZR on the YO was exciting. Analysis of the *Carma*_YO-transcriptome revealed that this receptor was not differentially expressed over the moult cycle (Table 4, Fig. 13B). Differential expression of CRZR between YO and epidermis was validated by qPCR: two-way ANOVA revealed no interaction effect (tissue x moult stage), but main effects revealed a significant difference in CRZR mRNA expression between tissues (being greater in the YO; F_(1,46)_ = 63.3, *P* < 0.001) but not across moult stage, F_(4, 46)_ = 1.01, *P* = 0.412 (Fig. 13C, D).

**Table 4:** G protein-coupled receptors upregulated in the Y organ

Annotation	Carma_YO_vs_Epi				Carma_YO							
	Cluster	LogFC	*P*	FDR	Cluster	LogFC				F	*P*	FDR
						A-B	D1	D3	D4			
Gonadotropin-releasing hormone receptor	Cluster-3919.3796	11.07	2.14E-78	1.08E-74	Cluster-136,339.18555	−0.41	−0.43	0.59	−0.39	1.42	0.259	0.437
Tachykinin-like peptides receptor 86C	Cluster-10,974.0	8.32	1.49E-15	4.06E-14	Cluster-136,339.45038	−1.12	0.26	0.24	−0.94	0.38	0.824	0.913
Relaxin receptor 1	Cluster-3919.12767	6.20	5.35E-41	1.27E-38	Cluster-131,500.3	−0.19	0.25	−0.29	− 0.35	1.49	0.805	0.902
Rhodopsin, G0-coupled	**Cluster-3919.2920**	**4.30**	**8.58E-17**	**2.71E-15**	**Cluster-62,481.4**	**0.27**	**0.37**	**−1.21**	**−2.04**	**8.31**	**< 0.001**	**0.004**
Rhodopsin, G0-coupled	**Cluster-3919.5747**	**2.60**	**2.83E-10**	**3.40E-09**	**.**	**.**	**.**	**.**	**.**	**.**	**.**	**.**
Probable G protein-coupled receptor Mth-like 1	**Cluster-69,371.0**	**2.31**	**4.77E-07**	**3.13E-06**	**Cluster-136,339.38316**	**1.31**	**−2.34**	**0.99**	**2.02**	**5.94**	**0.002**	**0.015**
Dopamine D2-like receptor	Cluster-3919.17236	2.18	0.002	6.76E-03	Cluster-96,405.0	0.37	−1.88	−0.75	−0.46	3.35	0.026	0.088
Probable G protein-coupled receptor Mth-like 1	Cluster-79,456.0	1.77	< 0.001	1.58E-03	Cluster-136,339.32542	3.81	−0.95	1.61	3.14	3.73	0.017	0.066
Opsin, ultraviolet-sensitive	Cluster-51,158.0	1.52	< 0.001	1.53E-03	Cluster-38,493.0	0.16	1.04	0.56	0.38	2.07	0.399	0.585
5-hydroxytryptamine receptor 4	Cluster-27,180.0	1.26	0.014	3.23E-02	Cluster-133,987.0	−0.59	−2.27	−2.08	−1.96	3.49	0.022	0.080
G protein-coupled receptor 161	Cluster-69,085.1	0.78	0.011	2.76E-02	Cluster-31,544.1	−0.40	0.89	1.54	1.16	2.59	0.062	0.164

Differential gene expression analysis statistics for clusters annotated with ‘GO:0004930 G protein-coupled activity’ and upregulated in YO relative to epidermis of *C. maenas* during inter-moult (C3–4), and across the moult cycle within the YO (*Carma*_YO-transcriptome) for those same GPCRs. Highlighted in bold are clusters differentially expressed over the moult cycle within the Y organ. ‘Rhodopsin, G0-coupled’ was clustered into two clusters within the *Carma_*YO_vs_Epi-transcriptome, but within a single cluster in the *Carma*_YO-transcriptome. For *Carma_*YO, logFC are relative to C3–4. The *P*-value should be read in conjunction with the false discovery rate (FDR)

**Fig. 14:**
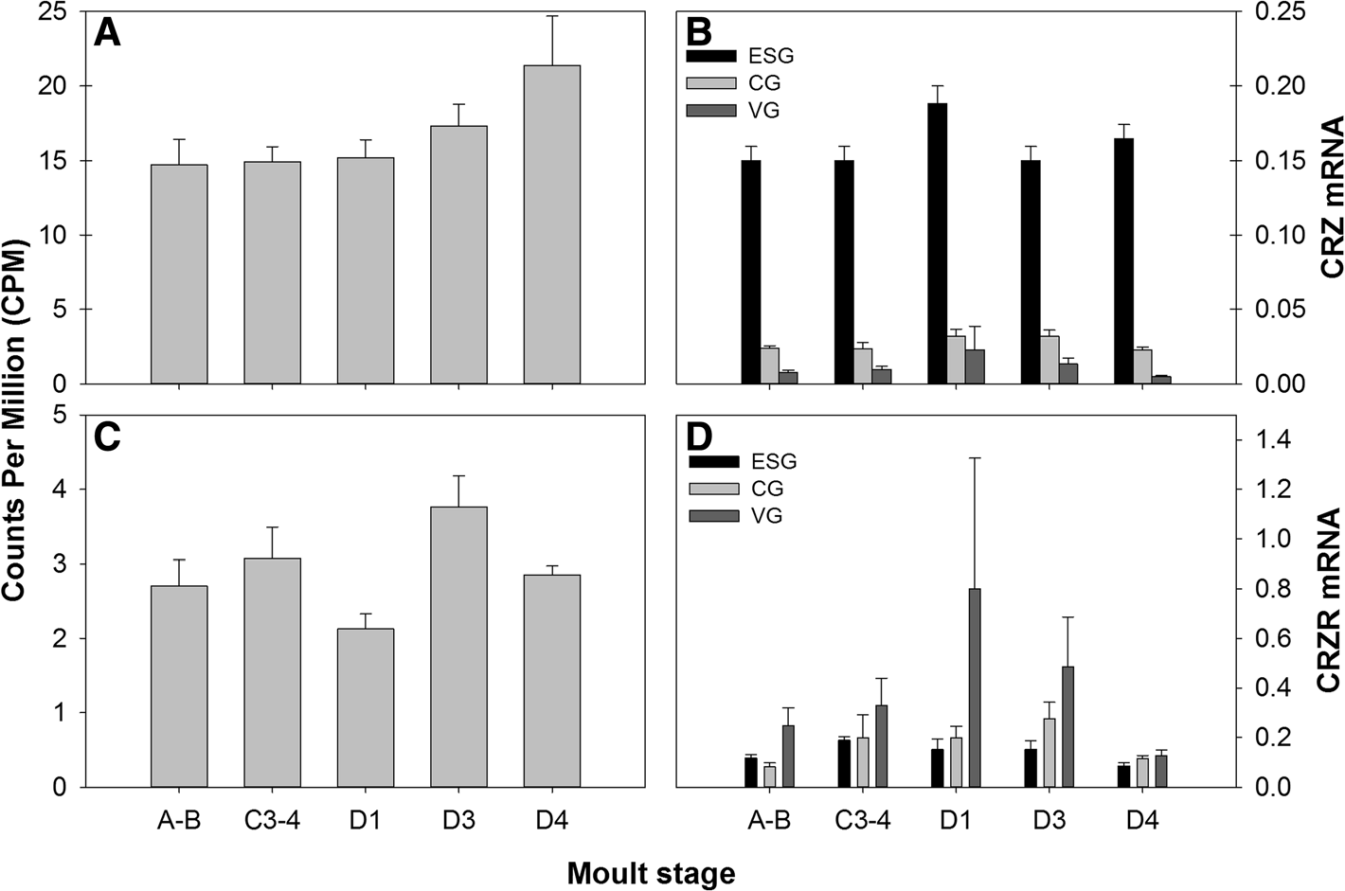
Corazonin (CRZ) (**A**, **B**) and corazonin receptor (CRZR) (**C**, **D**) gene expression across the moult cycle within the central nervous system of *C. maenas*, determined by RNAseq (**A**, **C**) and qPCR (**B**, **D**). For qPCR data, CRZ and CRZR copy numbers are normalized to the geometric mean of the reference genes *elongation factor 1-alpha* and *ubiquitin-conjugating enzyme* E2 L3 (data expressed as mean + SEM). In silico data are presented as mean counts per million (CPM + SEM)

We also performed qPCR on CNS tissues to verify expression patterns of both the peptide CRZ and the putative CRZR (Fig. 14). In silico data for CRZ revealed no differential expression across the moult cycle. Two way ANOVA of qPCR data however, showed that whilst there was no interaction between moult cycle and different CNS tissues, main effects were significant for tissue (F_(2,71)_ = 482.77, *P* < 0.001) and moult stage (F_(4,71)_ = 4.27, *P* = 0.004). Pairwise comparisons with Bonferroni correction post hoc analysis indicated that moult stage D1 differed from all other stages (*P* < 0.05), mRNA abundances being higher during this stage. For tissues, CRZ mRNA abundance was significantly greater within the ESG relative to CG and VG (*P* < 0.05). For the putative CRZR within the CNS, in silico data showed no differential gene expression across the moult cycle. Two-way ANOVA of CRZR qPCR data revealed no interaction between moult stage and tissue, but main effects were significant for tissue (F_(2,71)_ = 4.75, *P* = 0.0116); Bonferroni post-hoc analysis showed that VG CRZR mRNA expression was greater than ESG and CG (*P* < 0.05; Fig. 14D). Expression values for CRZR determined via qPCR show that expression levels within all regions of the CNS were low relative to expression within the YO (Figs. 13D, 14D). This putative CRZR has subsequently been de-orphanised as the receptor to the native CRZ neuropeptide [31].

CRZ qPCR identified the primary location of peptide synthesis within the CNS to be the eyestalk ganglion (ESG, Fig. 14), whilst CRZR was expressed at far higher values in the YO relative to all parts of the CNS. Intriguingly, given the crucial role of the YO to ecdysteroidogenesis and therefore ecdysis, high values for CRZR expression in the YO clearly indicate a hitherto unknown and unexpected role for this ligand-receptor pair in ecdysis regulation.

## Conclusions

The *Carma_*CNS-, *Carma_*YO-, and *Carma*_YO_vs_Epi- transcriptomes presented here provide a high quality resource for transcript discovery in relation to crustacean ecdysis regulation. The *Carma_*CNS-transcriptome represents the most complete crustacean transcriptome published to date in terms of neuropeptide and neuropeptide receptor sequence diversity and completeness. Moreover, the temporal element of this study has enabled the first comprehensive exploration of putative crustacean neuropeptide signaling across the moult cycle and across multiple tissues. Within the CNS of *C. maenas*, the neuropeptides carcikinin/ETH, CHH-2, and CCAP were differentially expressed across the moult cycle and are, therefore, anticipated to play important roles in the regulation of ecdysis. ETH and CCAP are fundamental to the temporal regulation of ecdysis in insects and release of CCAP during crustacean ecdysis is concomitant with the onset of ecdysis motor programme. The probable roles for both carcikinin/ETH and CCAP in crustacean ecdysis hint at conserved commonalities between insect and crustacean ecdysis cassettes. CHH-2, previously not studied, is now a neuropeptide of interest with respect to ecdysis physiology. The differing gene expression dynamics of CHH-2 relative to CHH-1, MIHs, and CHH-MIH-like peptides found here suggests that these CHHs may fulfil different physiological functions. However, since the translation and processing of functional peptides may be temporally uncoupled from mRNA expression, conclusions peptides availability and bioactivity of these cannot be inferred from mRNA abundance without due caution. A putative proctolin R1 was differentially expressed across the moult cycle, which marks a novel association of proctolin with ecdysis, but no other putative neuropeptide GPCR was found to be differentially expressed across the moult cycle in the CNS of *C. maenas*.

To our surprise, neuropeptide transcripts were discovered within the YO and amongst these neuroparsin-1 was found to be differentially expressed across the moult cycle, highlighting the YO as a source of neuropeptide synthesis. A number of putative neuropeptide receptors were identified as being differentially expressed across the moult cycle within the YO and, given that the only known function of the YO is synthesis of ecdysteroids, are putative candidates in ecdysis regulation.

Our analysis revealed relatively few GPCRs specific to the YO (compared to the epidermis). The most differentially expressed of these were essentially absent from epidermis and included a receptor annotated as gonadotropin-releasing hormone receptor which, considering phylogenetic grouping was revealed as a candidate corazonin receptor. Tantalisingly, this putative CRZR was strongly expressed in the YO relative to the epidermis, and was lowly expressed in the CNS, suggesting an unexpected and potentially very important role for CRZ-CRZR pair in ecdysis regulation.

The comprehensive list of putative GPCRs discovered here provides a basis from which receptor de-orphanisation may advance, which is absolutely necessary to enable neuropeptide and receptor pair function to be defined.

## Additional files

Additional file 1: **Figures S1**-**S13** and **Tables S1**-**S9**. (DOCX 3567 kb)

Additional file 2: Trinotate annotation reports and mined sequences for neuropeptides and G protein-coupled receptors. (XLSX 82011 kb)

Additional file 3: Neuropeptide prepro-hormone processing and deduced mature neuropeptide and precursor-related peptides. (DOCX 88 kb)

## Abbreviations

aa: Amino acid
ACP: Adipokinetic hormone/corazonin-related peptide
AG: Abdominal ganglia
AKH: Adipokinetic hormone
CCAP: Crustacean cardioactive peptide
cDNA: Complementary deoxyribonucleic acid
CFSH: Crustacean female sex hormone
CG: Cerebral ganglia
CHH: Crustacean hyperglycemic hormone
CNS: Central nervous system
cRNA: Complementary ribonucleic acid
CRZ: Corazonin
CRZR: Corazonin receptor
DEPC: Diethyl pyrocarbonate
DH: Diuretic hormone
EF1a: Elongation factor 1-alpha
EH: Eclosion hormone
ESG: Eyestalk ganglia
ETH: Ecdysis triggering hormone
ETHR: Ecdysis triggering hormone receptor
GPCR: G protein-coupled receptor
IAG: Androgenic insulin-like peptide
LGR: Leucine-rich repeat-containing
MIH: Moult-inhibiting hormone
MOIH: Mandibular organ inhibitory hormone
mRNA: Messenger ribonucleic acid
Mth: Methuselah
NGS: Next generation sequencing
NPFR: Neuropeptide F receptor
OEH: Ovary ecdysteroidogenic hormone
OMP: Ovary maturing parsin
ORF: Open reading frame
PAGE: Polyacrylamide gel electrophoresis
PRP: Precursor related peptide
PTTH: Prothoracicotropic hormone
RNA: Ribonucleic acid
RPCH: Red pigment concentrating hormone
RT: Room temperature
RT-PCR: Reverse transcription polymerase chain reaction
sNPFR: Short neuropeptide F receptor
SOG: Sub-oesophageal ganglia
TG: Thoracic ganglia
UBE2L3: Ubiquitin-conjugating enzyme E2 L3
VG: Ventral ganglia
VIH: Vitellogenesis-inhibiting hormone
Vm: Ventro-medial
YO: Y organ
