# Supplementary material for: Transcriptomic analysis of crustacean neuropeptide signaling during the moult cycle in the green shore crab, Carcinus maenas
Source: BMC Genomics. 2018 Sep 26;19:711. doi: 10.1186/s12864-018-5057-3 (PMC6158917; doi:10.1186/s12864-018-5057-3)
Supplement: Supplementary file 1 — Figures S1-S13 and Tables S1-S9. (DOCX 3567 kb) [file 12864_2018_5057_MOESM1_ESM.docx]

**Transcriptomic analysis of crustacean neuropeptide signaling during the molt cycle in the green shore crab, *Carcinus maenas*.**

Andrew Oliphant^1^, Jodi L. Alexander^2^, Martin T. Swain^1^, Simon G. Webster^2^, David C. Wilcockson^1,^*

^1^Institute of Biological, Environmental and Rural Sciences, Aberystwyth University, Aberystwyth, Ceredigion SY23 3DA, UK

^2^School of Biological Sciences, Bangor University, Bangor, Gwynedd LL57 2UW, UK


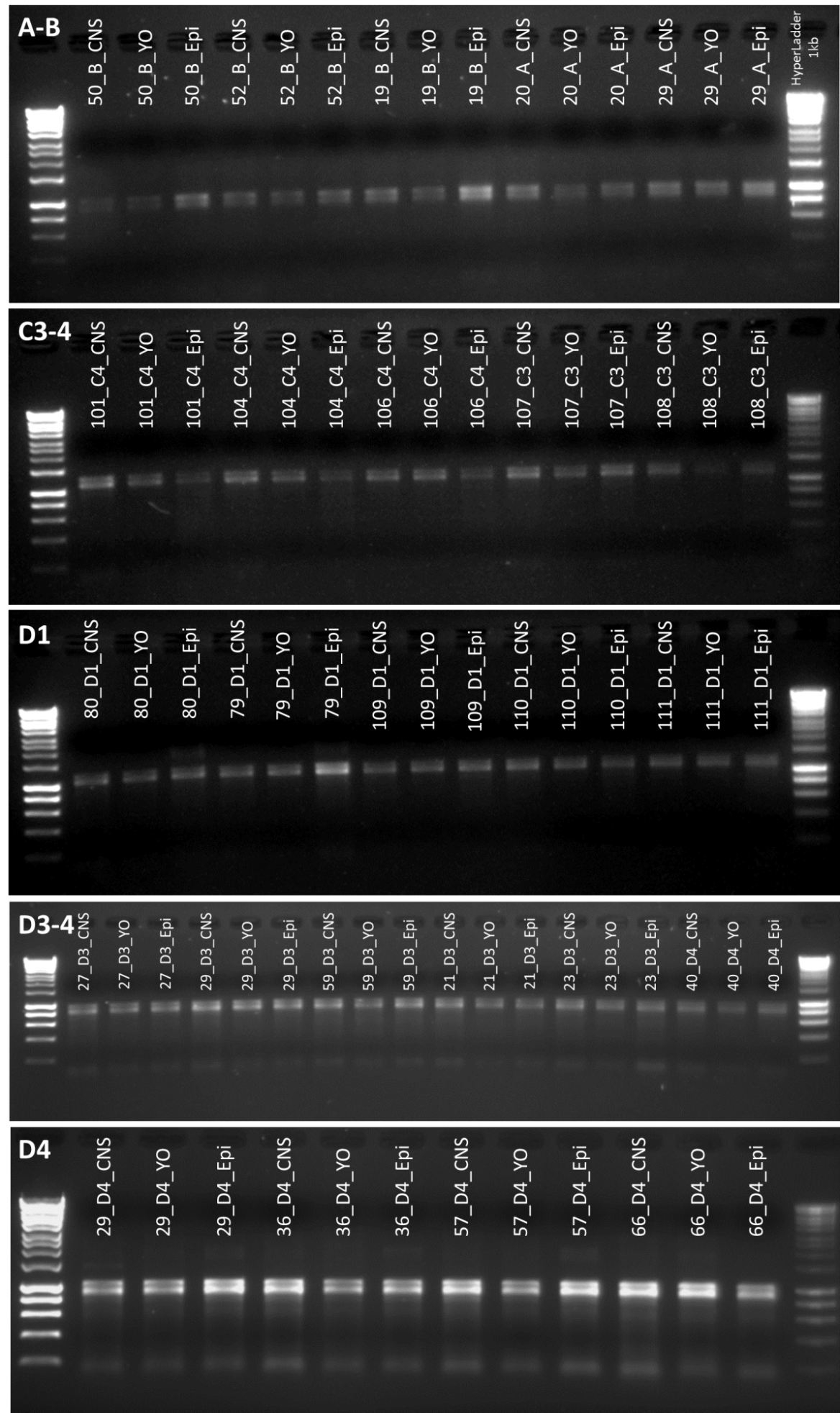


**Additional file 1: Figure S1**

Composite image of gel electrophoresis assessment of total RNA integrity used for RNA sequencing. CNS = central nervous system, YO = Y organ, Epi = epidermis.

**Assembler comparison**

A number of descriptive statistics (generated using TransRate [1]) suggested that the BinPacker and Oasis assemblies may be better than the Trinity assembly; for example, BinPacker mean length = 1114.54bp (larger that Trinity, mean length = 663.65bp) and Oasis N50 = 2760bp (larger than Trinity, N50 = 908bp). However, whilst the sequence length of contigs generated by Trinity was shorter than the other assemblers, the percentage of contigs with an ORF was highest for the Trinity assembly (mean ORF percent = 47.63 %; Supplementary Table 1). When assessed further, Trinity had the highest number of *Carcinus maenas* neuropeptide BLASTx hits and the highest protein coverage lengths, and only the Oasis assembly was comparable for SwissProt BLASTx hits and coverage length (Supplementary Fig. 2). Oasis generated long contigs and had a high number of SwissProt BLASTx hits (similar to Trinity), but also produced very many short and fragmented contigs (e.g. 359,903 under 200bp!). Analysis of SwissProt BLASTx hits found that each assembler generated unique BLAST hits (Supplementary Fig. 3), with Oasis having the greatest number of SwissProt BLASTx hits not found in the other transcriptomes. However, only Trinity had neuropeptide BLASTx hits that were not found in other assemblers (Supplementary Fig. 3). Finally, analysis of the transcriptomes using BUSCO v3.0.2 (run with default settings and using the metazoan_odb9 dataset) identified a similar number of metazoan genes for the Trinity assembly (915) and the Oases assembly (955) and slightly lower numbers for the BinPacker (914) and IDBA-tran (918) assemblies, though all assemblies contained >93% of metazoan genes as complete genes (Supplementary Table 1). Trinity and Oases assemblies contained low numbers of fragmented and missing metazoan genes (Supplementary Table 1). Mapping statistics, coupled with BLAST hits and BUSCO analysis led us to conclude that Trinity assembled the superior transcriptome for our analysis and thus, this assembly was used for our study. Oasis also assembled a good assembly, but contained high numbers of very similar contigs and high numbers of very short contigs; for example, of the complete metazoan genes identified by BUSCO 866 were complete and duplicated BUSCOs whilst for Trinity only 625 were complete and duplicated BUSCOs.

**Additional file 1: Table S1**

Descriptive statistics for transcriptomes assembled using four different assemblers.

|  | **Trinity** | **BinPacker** | **IDBA-tran** | **Oasis** |
| --- | --- | --- | --- | --- |
| **Number of contigs** | 725786 | **379680** | 482229 | 1481885 |
| **Smallest contig** | 224 | 200 | **300** | 100 |
| **Largest contig** | 31903 | 41572 | 55922 | **85340** |
| **Bases** | 4.82E+08 | 4.23E+08 | 4.45E+08 | 1.38E+09 |
| **Mean length** | 663.65 | **1114.54** | 923.54 | 897.11 |
| **Number under 200 bp** | **0** | **0** | **0** | 359903 |
| **Number over 1k bp** | 97795 | 107661 | 109558 | 329828 |
| **Number over 10k bp** | 673 | 2266 | 1099 | 5906 |
| **Number of ORFs** | 85220 | 64736 | 76320 | 233334 |
| **Mean ORF percent** | **47.63** | 34.64 | 40.01 | 34.6 |
| **N90** | 283 | 441 | 397 | 436 |
| **N70** | 466 | 960 | 688 | 1230 |
| **N50** | 908 | 1971 | 1238 | **2760** |
| **N30** | 2089 | 3944 | 2499 | 5021 |
| **N10** | 5076 | 8599 | 6007 | 9712 |
| **GC proportion** | 0.43665 | 0.4339 | 0.43198 | 0.40607 |
|  |  |  |  |  |
| **BUSCO (978 genes)** |  |  |  |  |
| **complete** | 97.2% (951) | 93.5% (914) | 93.9% (918) | 97.6% (955) |
| **fragmented** | 2.1% (21) | 4.4% (43) | 5.3%  (52) | 1.8% (18) |
| **missing** | 0.7% (6) | 2.1% (21) | 0.8% (8) | 0.6% (5) |

Assemblers used: Trinity v2.0.6 [2], BinPacker v1.1 [3], IDBA-tran v1.1.1 [4], and Oasis v0.2.8 [5] (which used Velvet v1.2.10 [6]). Statistics were calculated using TransRate v1.0.3 [1] and BUSCO v3.0.2.


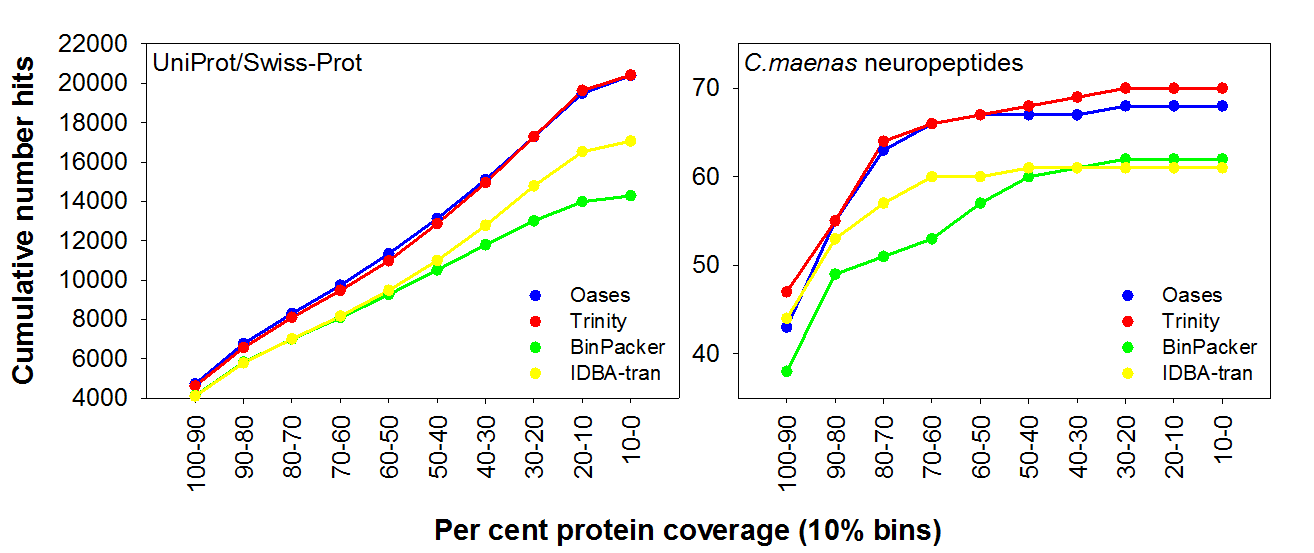


**Additional file 1: Figure S2**

Cumulative BLASTx hits and percent protein coverage for the UniProt SwissProt database and a database of *Carcinus maenas* neuropeptides (generated from previously published sequences [7]).


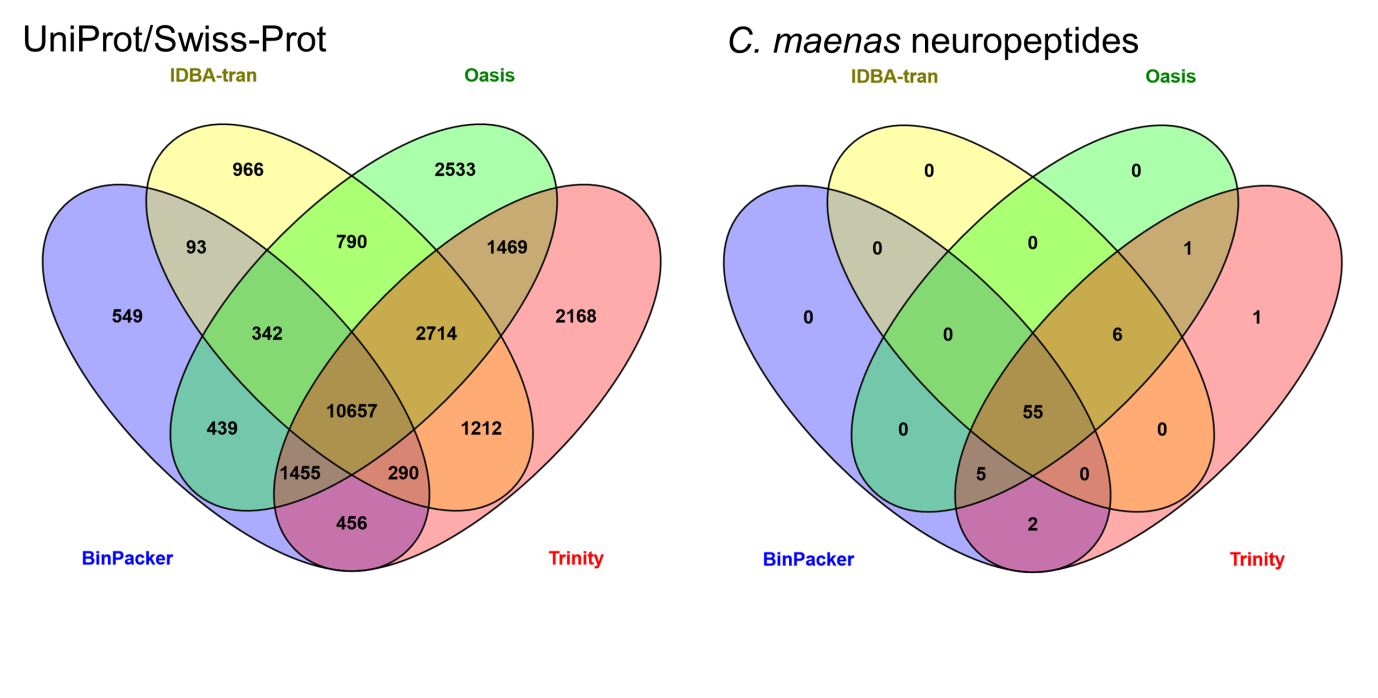


**Additional file 1: Figure S3**

Venn diagrams of gene numbers identified by BLASTx using the UniProt SwissProt database and a database of *Carcinus maenas* neuropeptides (generated from previously published sequences [7]) for transcriptomes assembled *de novo* using different assemblers. Venn diagrams were made using the online tool Venny [8].

**Additional file 1: Table S2**

Primer and probe sequences for quantitative Taqman PCR and end-point PCR.

| **Method** | **Oligonucleotide name** | **Sequence (5’-3’)** |
| --- | --- | --- |
| **Carcikinin Taqman qPCR assay** | Carcikinin_F_TAQMAN | GGCGGGCCTGGACAGT |
|  | **Probe:** Carcikinin-VIC | VIC-CGGAGACGGTTGTATTG-MGB |
|  | Carcikinin_R_TAQMAN | GGGATACGTAGCAGCTCTCCAA |
| **CRZ Taqman qPCR assay** | Cam_cor_F_TAQMAN | CGAATGGGCGGAAAAGG |
|  | **Probe:** CamCOR_NED | NED-CCAATGACCTGGGCAC-MGB |
|  | Cam_cor_R_TAQMAN | CCACTGCCAAGACCCACAAC |
| **CRZR Taqman qPCR assay** | CamCRZR Taqman F’ | AGGCTGCTGAGGAAGGCTAAG |
|  | CamCRZR Taqman probe | NED-TGCGAGCCCTCCG-MGB |
|  | CamCRZR Taqman R’ | CCAACACGATCACCACAGAGA |
| **EH-1 Taqman qPCR assay** | EH1_TAQman_F | CAGTAACTGCGGCCAATGC |
|  | **Probe:** Cam_EH-1_FAM | FAM-AGGAGATGTACGGCGACT-MGB |
|  | EH1_TAQman_R | CGCCTGGCCGTGGAA |
| **UBE2L3 qPCR Taqman assay** | Ca.M_UBC TaqMan F | TCACCTGGCAGGGACTCATT |
|  | **Probe:** camUBC-FAM | FAM-ACCCGAGAACCCACC-MGB |
|  | Ca.M_UBC TaqMan R | CCTGAACGCTCCCTTGTTGT |
| **EF1a Taqmand qPCR assay** | Ca.M_EF1A TaqMan F | GAGCGGCAGCTATGAGTTCAT |
|  | **Probe:** camEF1A-VIC | VIC-CTCTCTTTGACGCTCTGG-FAM |
|  | Ca.M_EF1A TaqMan R | TGGATGGAGGCTCAATGTTG |
|  |  |  |
| **Carcikinin standard synthesis** | Carcikinin_F_T7 | T7-TCAAGCTGCTCAACGATCTG |
|  | Carcikinin_R | AGGTGTCTTCGGTAGCGTGT |
| **CRZ standard synthesis** | CamCor_F_T7 | T7-CTCCGTGCCAGGTTGTCTAT |
|  | CamCor_R | GTGTGGGAAGCGTTGTTTCT |
| **CRZR standard synthesis** | CamCRZR STD F’ | CTCGACGTGGAGGAAAACAT |
|  | CamCRZR STD T7 R’ | T7-AGGAAGTCCTTCGCTTGTGA |
| **EH-1 standard sythesis** | CaM_EH_LKRFI_F-T7 | T7-CCACAGCTCTTCTCCTGTCC |
|  | CaM_EH_LKRFI_R | AAATTACGACGCGACCTGAC |
| **UBE2L3 standard synthesis** | Ca.M_UBC F T7 | T7-ACATTCGAAGGTCTGGCATC |
|  | Ca.M_UBC R | CCAAAGACCGCAAGAAGTTC |
| **EF1a standard synthesis** | Ca.M_EF1A_F_T7 | T7-CCAAGATCGAGCGTAAGAGC |
|  | Ca.M_EF1A_R | TTTCACAGCTCAGGTGATCG |
|  |  |  |
| **CHH-2 end-point PCR** | CHH-2_F | CCACCAACATGTTCCAAAGA |
|  | CHH-2_R | CACAATCCTCGCACACGTAG |
| **EH-1 end-point PCR** | CaM_EH_LKRFI_F | CCACAGCTCTTCTCCTGTCC |
|  | CaM_EH_LKRFI_R | AAATTACGACGCGACCTGAC |
| **EH-2 end-point PCR** | CaM_EH_F1 | TAGGATACCCGTTCGCTGAC |
|  | Cam_EH_R1 | TGTGGACGTGTGTGTGTGTC |
| **EF1a end-point PCR** | Ca.M_EF1A_F | CCAAGATCGAGCGTAAGAGC |
|  | Ca.M_EF1A_R | TTTCACAGCTCAGGTGATCG |

Each Taqman assay includes a forward and reverse primer (F’ and R’) and a hydrolysis probe labeled at the 5’ end with a fluorescent dye and a minor groove binding 3’ end. ‘Standard synthesis’ refers to primer pairs used in the generation of PCR templates for in vitro transcription of qPCR standard curves. The T7 phage promoter site (T7) used had the sequence 5’-TAATACGACTCACTATAGGG-3’.


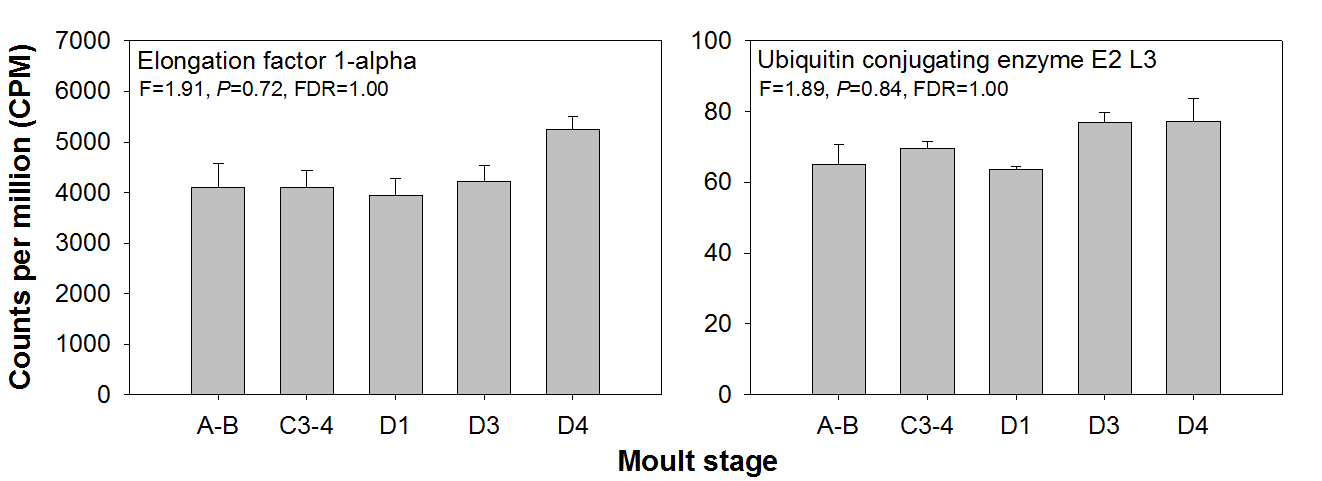


**Additional file 1: Figure S4**

Reference genes for qPCR expression across moult cycle. RNA-seq found both to be constituently expressed and thus used as reference genes in normalisation.

**Transcriptome assembly and annotation- summary data**

For CNS RNA, Illumina HiSeq2500 sequencing of 25 indexed cDNA libraries yielded a total of 785.2M 126-bp paired-end reads, 785.0M of which survived quality control (trimming) and were used for *de novo* assembly (Supplementary Table 5). Of these quality reads, 765.6M (97.5 % of raw reads) were paired and were used for mapping with Bowtie for Corset clustering and gene expression analysis. Trinity assembled a transcriptome comprising 725,786 transcripts assigned to 583,758 ‘genes’ with an N50 of 908 bp and a mean transcript length of 663.7 bp. Clustering with Corset reduced the number of transcripts to 396,085 assigned to 260,340 clusters, with a mean transcript length of 946.5 bp and an N50 of 1,430 bp (Supplementary Table 3). Transcriptome annotation with Trinotate assigned UniProt/Swiss-Prot annotations to 98,380 transcripts (Supplementary Table 4). TransDecoder identified ORFs in 129,772 transcripts. Of these ORFs, UniProt/Swiss-Prot annotations were assigned to 71,515 (55.1 % of ORFs) and conserved Pfam domains were assigned to 58,861 (45.4% of ORFS, Supplementary Table 4). Transmembrane helices were predicted in 22,736 ORFs and signal peptides were predicted in 9,449 ORFs (Supplementary Table 4).

**Additional file 1: Table S3**

Descriptive statistics for *Carma* transcriptomes

| **Statistics** | ***Carma_*CNS** | ***Carma_*YO** | ***Carma*_YO_vs_Epi** |
| --- | --- | --- | --- |
| **Number of Trinity 'genes'** | 583,758 | 373,804 | 309,854 |
| **Number of transcripts** | 725,786 | 509,161 | 400,391 |
| **Number of Corset clusters** | **260,340** | **251,194** | **227,967** |
| **Number of transcripts** | **396,085** | **348,186** | **202,321** |
| **Minimum transcript length (bp)** | **224** | **224** | **224** |
| **Maximum transcript length (bp)** | **31,903** | **105,735** | **30,550** |
| **Mean transcript length (bp)** | **946.5** | **946.4** | **1,155.6** |
| **N50 (bp)** | **1,430** | **1,584** | **1,977** |
| **Total assembled bases (bp)** | **374,911,960** | **329,539,443** | **233,799,603** |

Values in bold lettering highlight statistics for ‘Corset clustered’ transcriptomes

For YO RNA, sequencing of 25 libraries yielded 791.7M 126-bp paired-end reads, 791.5M of which were quality reads and were used for transcriptome assembly and 783.6M (98.9 % of raw reads) were quality paired reads and were used in mapping (Supplementary Table 6). Trinity assembled a transcriptome comprising 509,161 transcripts assigned to 373,804 ‘genes’ with an N50 of 1222 bp and an average contig length of 743.8 bp. Corset clustering reduced the number of transcripts to 348,186 assigned to 251,194 clusters with an average contig length of 946.4 bp and an N50 of 1,584 bp (Supplementary Table 3). Trinotate annotation assigned BLASTx UniProt/Swiss-Prot hits to 89,506 transcripts and TransDecoder identified ORFs in 115,605 transcripts (Supplementary Table 4). Of these ORFs, 63,928 were assigned BLASTP UniProt/Swiss-Prot hits and 59,565 were assigned Pfam hits (Supplementary Table 4).

For YO and epidermis inter-molt (C3-4) RNA, sequencing of 10 libraries yielded 270.0M 126-bp paired-end reads, 269.9M of which were quality reads and 263.1M (97.5 % of raw reads) were paired quality reads (Supplementary Table 7). Trinity assembled 400,391 contigs assigned to 309,854 ‘genes’ with an average contig length of 743.63 bp and an N50 of 1291 bp. Corset clustering reduced the number of transcripts to 227,967 assigned to 202,321 clusters with an average contig length of 1,155.6 bp and an N50 of 1,977 bp (Supplementary Table 3). Annotation assigned UniProt/Swiss-Prot annotations to 74,186 transcripts. A total of 93,235 ORFs transcripts were identified using Transdecoder and UniProt/Swiss-Prot annotations were assigned to 54,629 of these, whilst Pfam annotations were assigned to 52,266 ORFs (Supplementary Table 4).

**Additional file 1: Table S4**

Annotation statistics for *Carma* transcriptomes

| **Annotation** | ***Carma_*CNS** | ***Carma*_YO** | ***Carma*_YO_vs_Epi** |
| --- | --- | --- | --- |
| **BLASTx (UniProt/Swiss-Prot)** | 98,380 (24.8 % of transcripts) | 89,506 (25.7 % of transcripts) | 74,186 (32.5 % of transcripts) |
| **RNAMMER** | 40,339 (10.2 % of transcripts) | 11,001 (3.2 % of transcripts) | 46,373 (20.3 % of transcripts) |
| **TransDecoder ORF finder** | 129,772 (32.8 % of transcripts) | 115,605 (33.2 % of transcripts) | 93,235 (40.9 % of transcripts) |
| **BLASTP (UniProt/Swiss-Prot)** | 71,515 (55.1 % of ORFs) | 63,928 (55.3 % of ORFs) | 54,629 (58.6 % of ORFs) |
| **Pfam** | 58,861 (45.4 % of ORFs) | 59,565 (51.5 % of ORFs) | 52,266 (56.1 % of ORFs) |
| **TMHMM** | 22,736 (17.5 % of ORFs) | 18,950 (16.4 % of ORFs) | 16,028 (17.2 % of ORFs) |
| **SignalP** | 9,449 (7.3 % of ORFs) | 7,469 (6.5 % of ORFs) | 6,411 (6.9 % of ORFs) |
| **eggNOG** | 67,735 | 60,512 | 48,951 |
| **KEGG** | 71,657 | 64,631 | 52,741 |
| **BLAST gene ontologies** | 81,414 | 72,445 | 59,733 |
| **Pfam gene ontologies** | 38,771 | 38,745 | 33,327 |

Based on the results of an initial trial using CNS reads (see above), Trinity was selected to assemble transcriptomes for this study and has been shown to perform well in other studies. However, in common with all assemblers, Trinity reconstructs more contigs than is biologically realistic. Often many contigs are derived from the same gene, and some of these sequences are incorrect reconstructions of the genuine mRNA transcripts. Moreover, neuropeptide transcripts often contain repeating sequences, which make the reconstruction of long DNA sequences from short reads problematic [7]. Similarly, alternative splicing of transcripts and allelic variation can be an issue for assemblers [7]. The *Carma_*CNS- and *Carma_*YO-transcriptomes assembled here each used RNA from 25 individual crabs taken from a natural population whilst the *Carma_*YO_vs_Epi-transcriptome used RNA from 10 such animals, thus there was likely considerable polymorphism relative to datasets derived using lines of in-bred organisms. Repetitive regions, sites of alternative splicing, and allelic variation are probable causes of the incomplete reconstruction of transcripts or the fragmentation of a single transcript into multiple transcripts within this study, especially of lowly expressed genes. Despite the potential intractable limitations, neuropeptide diversity and sequence completeness within our *Carma_*CNS-transcriptome is comparable to, or exceeds, other recently published *C. maenas* neurotranscriptomes.

**Additional file 1: Table S5**

Raw reads, quality, and quality paired reads used to assemble *Carma­_*CNS-transcriptome

| **Sample and moult stage** | | **Number of raw reads** | **Number of quality reads** | **Number of paired quality reads** |
| --- | --- | --- | --- | --- |
| **20_A** | A-B | 23,747,176 | 23,739,446 | 22,997,763 |
| **29_A** | A-B | 27,484,462 | 27,477,185 | 26,677,582 |
| **19_B** | A-B | 34,099,308 | 34,089,793 | 33,101,006 |
| **50_B** | A-B | 61,160,279 | 61,151,097 | 59,803,614 |
| **52_B** | A-B | 34,725,717 | 34,714,346 | 33,672,892 |
| **107_C3** | C3-4 | 30,272,130 | 30,267,866 | 29,466,659 |
| **108_C3** | C3-4 | 27,772,860 | 27,770,136 | 27,240,273 |
| **101_C4** | C3-4 | 27,336,944 | 27,329,979 | 26,601,965 |
| **104_C4** | C3-4 | 26,932,256 | 26,924,866 | 26,144,958 |
| **106_C4** | C3-4 | 29,260,728 | 29,254,245 | 28,363,223 |
| **79_D1** | D1 | 22,836,820 | 22,831,535 | 22,238,338 |
| **80_D1** | D1 | 24,015,453 | 24,013,068 | 23,508,431 |
| **109_D1** | D1 | 28,440,115 | 28,434,555 | 27,750,617 |
| **110_D1** | D1 | 27,115,559 | 27,109,999 | 26,512,532 |
| **111_D1** | D1 | 27,351,742 | 27,346,080 | 26,683,746 |
| **21_D3** | D3 | 30,384,451 | 30,377,995 | 29,529,071 |
| **23_D3** | D3 | 26,353,360 | 26,347,367 | 25,638,178 |
| **27_D3** | D3 | 27,268,667 | 27,263,659 | 26,633,428 |
| **29_D3** | D3 | 25,600,038 | 25,594,341 | 24,842,108 |
| **59_D3** | D3 | 26,249,151 | 26,243,245 | 25,519,331 |
| **29_D4** | D4 | 30,770,995 | 30,764,390 | 30,358,203 |
| **36_D4** | D4 | 33,856,747 | 33,850,835 | 33,184,735 |
| **57_D4** | D4 | 35,856,162 | 35,850,869 | 35,229,321 |
| **66_D4** | D4 | 62,998,487 | 62,986,766 | 61,672,124 |
| **40_D4** | D4 | 33,277,048 | 33,269,372 | 32,239,395 |
| **Total** | | **785,166,655** | **785,003,035** | **765,609,493** |

**Additional file 1: Table S6**

Raw reads, quality, and quality paired reads used to assemble *Carma*_YO-transcriptome

| **Sample and moult stage** | | **Number of raw reads** | **Number of quality reads** | **Number of paired quality reads** |
| --- | --- | --- | --- | --- |
| **20_A** | A-B | 29,509,564 | 29,500,864 | 29,147,567 |
| **29_A** | A-B | 27,516,519 | 27,508,229 | 27,203,383 |
| **19_B** | A-B | 49,000,795 | 48,965,757 | 48,330,972 |
| **50_B** | A-B | 33,940,066 | 33,928,090 | 33,459,844 |
| **52_B** | A-B | 42,785,942 | 42,772,219 | 42,263,469 |
| **101_C4** | C3-4 | 35,544,663 | 35,535,402 | 35,176,901 |
| **104_C4** | C3-4 | 24,062,361 | 24,056,034 | 23,802,907 |
| **106_C4** | C3-4 | 28,643,792 | 28,637,561 | 28,364,637 |
| **107_C3** | C3-4 | 28,647,199 | 28,641,744 | 28,365,153 |
| **108_C3** | C3-4 | 25,256,794 | 25,254,446 | 25,066,147 |
| **109_D1** | D1 | 29,050,048 | 29,045,084 | 28,830,738 |
| **110_D1** | D1 | 26,459,219 | 26,453,452 | 26,205,987 |
| **111_D1** | D1 | 32,420,460 | 32,413,655 | 32,077,052 |
| **79_D1** | D1 | 32,483,066 | 32,477,387 | 32,214,415 |
| **80_D1** | D1 | 27,206,710 | 27,203,664 | 27,003,591 |
| **21_D3** | D3 | 25,780,389 | 25,773,075 | 25,477,470 |
| **23_D3** | D3 | 22,786,230 | 22,780,205 | 22,536,309 |
| **27_D3** | D3 | 23,591,110 | 23,586,473 | 23,383,602 |
| **29_D3** | D3 | 43,961,238 | 43,950,375 | 43,501,305 |
| **59_D3** | D3 | 28,039,414 | 28,032,871 | 27,735,700 |
| **29_D4** | D4 | 38,700,932 | 38,695,491 | 38,472,106 |
| **36_D4** | D4 | 39,783,472 | 39,778,179 | 39,512,227 |
| **40_D4** | D4 | 21,685,864 | 21,680,853 | 21,442,492 |
| **57_D4** | D4 | 30,928,238 | 30,923,956 | 30,718,166 |
| **66_D4** | D4 | 43,867,052 | 43,856,489 | 43,351,964 |
| **Total** | | **791,651,137** | **791,451,555** | **783,644,104** |

**Additional file 1: Table S7**

Raw reads, quality, and quality paired reads used to assemble *Carma*_YO_vs_Epi-transcriptome

| **Sample and tissue** | | **Number of raw reads** | **Number of quality reads** | **Number of paired quality reads** |
| --- | --- | --- | --- | --- |
| **101_C4** | Y-organ | 35,544,663 | 35,534,686 | 34,545,871 |
| **104_C4** | Y-organ | 24,062,361 | 24,055,517 | 23,347,281 |
| **106_C4** | Y-organ | 28,643,792 | 28,636,981 | 27,829,949 |
| **107_C3** | Y-organ | 28,647,199 | 28,641,206 | 27,907,762 |
| **108_C3** | Y-organ | 25,256,794 | 25,254,092 | 24,741,696 |
| **101_C4** | Epidermis | 21,206,376 | 21,201,265 | 20,597,858 |
| **104_C4** | Epidermis | 29,668,004 | 29,660,367 | 28,865,860 |
| **106_C4** | Epidermis | 24,882,698 | 24,877,013 | 24,205,292 |
| **107_C3** | Epidermis | 27,373,430 | 27,371,373 | 26,896,779 |
| **108_C3** | Epidermis | 24,702,731 | 24,700,309 | 24,201,161 |
| **Total** | | **269,988,048** | **269,932,809** | **263,139,509** |

**Allatotropin**

Using *Daphnia pulex* allatotropin as a search term, a tBLASTn search on the NCBI website of the ‘Nucleotide collection (nr/nt)’ database and ‘crustaceans (taxid:6657)’ organism identified a transcript from the amphipod, *Hyalella azteca* (NCBI accession: XM_018171664.1). Similarly, a local tBLASTn search using *D.pulex* allatotropin (in BioEdit software [9]) identified a transcript encoding allatotropin within a brain-transcriptome generated for the amphipod, *Talitrus saltator* (NCBI BioProject: PRJNA297565, [10]). Below are *D.pulex* allatotropin and the translated peptides of the transcripts identified for *H.azteca* and *T.saltator*. Signal peptides in grey italics, active peptides in bold, dibasic cleavage sites underlined and highlighted yellow, and amidation sites in bold and highlighted yellow.

***Daphnia* *pulex* (EFX71302.1)**

*MKGKGAFLMVLAGWGLIGLMILTTAVEA*APHPADYTSSSVNNQRDFRSRR^**GFKTVGLATARGF**GKR^APSLSNFNSFQDAAEQMMQQQEENPNSDPDVFPVDWLVNYLQNKPDVIRYMVEHLLDHNGDGQVTSQEMMTSLQQQRED-

***Hyalella azteca*** **(XM_018171664.1)**

*MWWRVWLTLLLVLTVAVLS*GHSLELGLRRAALMARARQEAADRLRR^**GFQNSALATARGF**GKR^SHSDLHYDSHVYVLDISLSVLKWLDTQSFESPVEGADYGPVLSAADDGHWKLLEGTGRDRSLADGKRGGEAAALDKNSVPGERYPVSMVGELISSSPSLATSLVSRFLDADSDGYVSPKELVNVMQ-

***Talitrus saltator* (NCBI: PRJNA297565)**

*MTSRVCLLVVLVLGTVVLSSEA*LELGLRRAALMARARQEAADRLRR^**GFQNSALATARGF**GKR^GGDSPIRRPVDSLEIAELFADGNGGLDFGTGLGFLNGGHWKNFETAGRQKLRMEETKRSSEAMASNNVQRERYPVSMVGELISSSPSLATSLVSRFLDADSDGYVSPKELVNVMQ-

10

....|....|...

**Dp** **GFKTVGLATARGF**

**Ha** **GFQNSALATARGF**

**Ts** **GFQNSALATARGF**

**Additional file 1: Figure S5**

Protein alignment for allatotropin active peptide sequences from *Daphnia pulex* (*Dp*), *Hyalella Azteca* (*Ha*), and *Talitrus saltorus* (*Ts*). Identical residues highlighted black.


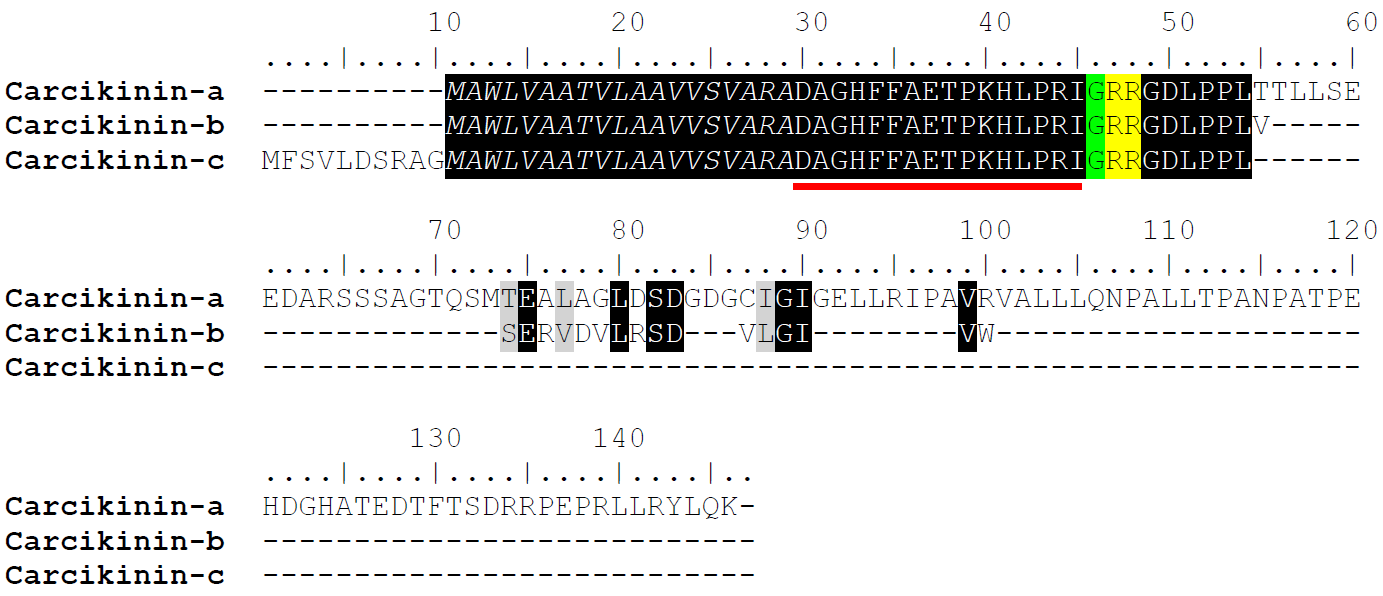


**Additional file 1: Figure S6**

Amino acid alignment for carcikinin/ETH protein sequences translated from transcripts discovered in the *Carma*_CNS-transcriptome. Whilst carcikinin-a and -b are full length, carcikinin-c is an N-terminal partial sequence. Predicted signal peptides are in italics. Identical residues are highlighted black, conserved substitutions are highlighted grey, dibasic cleavage sites are highlighted yellow and amidation sites are highlighted green. The mature peptide is underlined red.


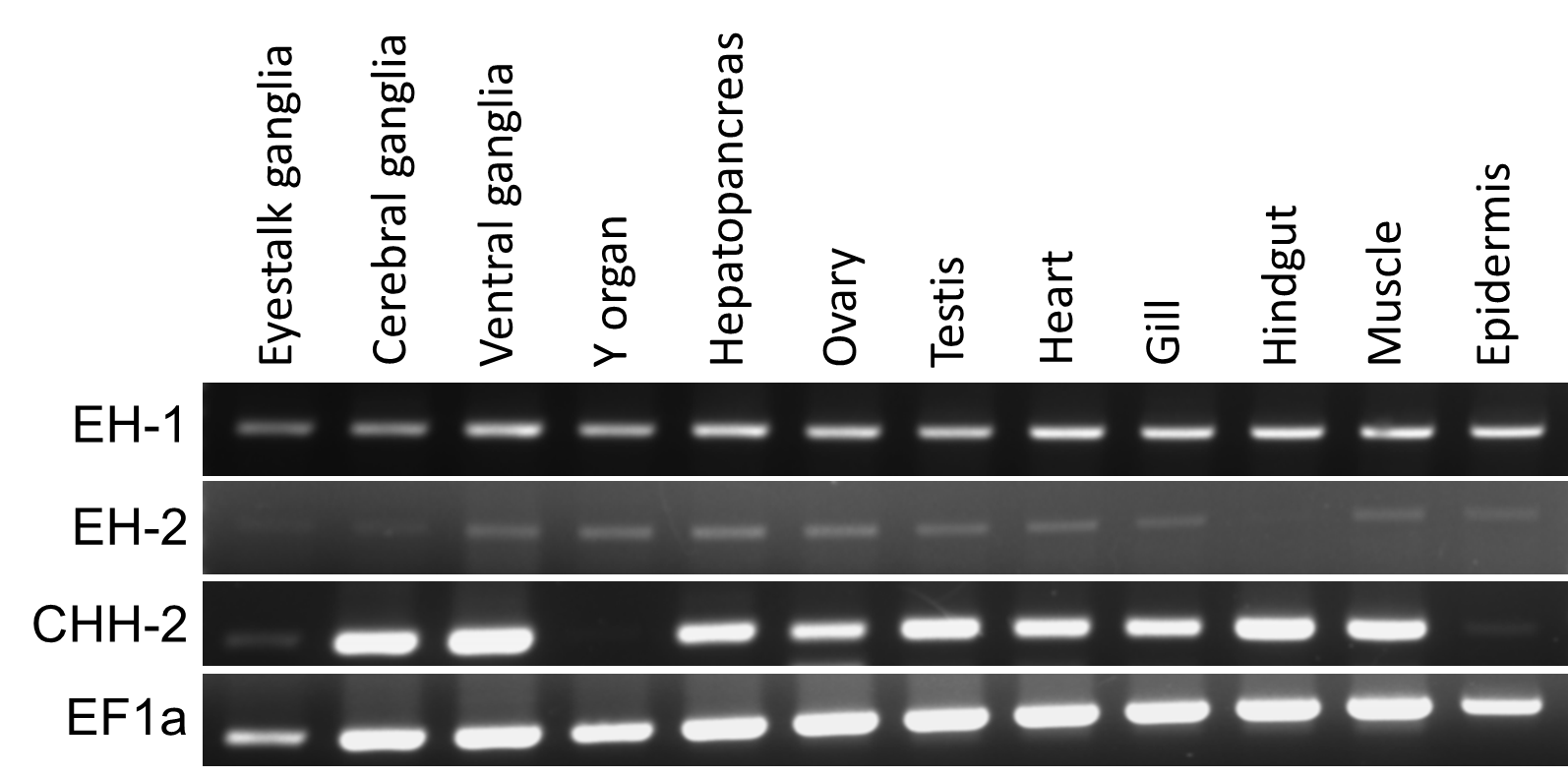


**Additional file 1: Figure S7**

Composite image of end-point PCR agarose gels performed to establish tissue distribution of eclosion hormone 1 and 2 (EH-1, EH-2) and crustacean hyperglycaemic hormone 2 (CHH-2) transcripts, and the reference gene elongation factor 1-alpha (EF1a). PCRs done using pooled cDNA from *n*=5 inter-moult (C4) crabs.


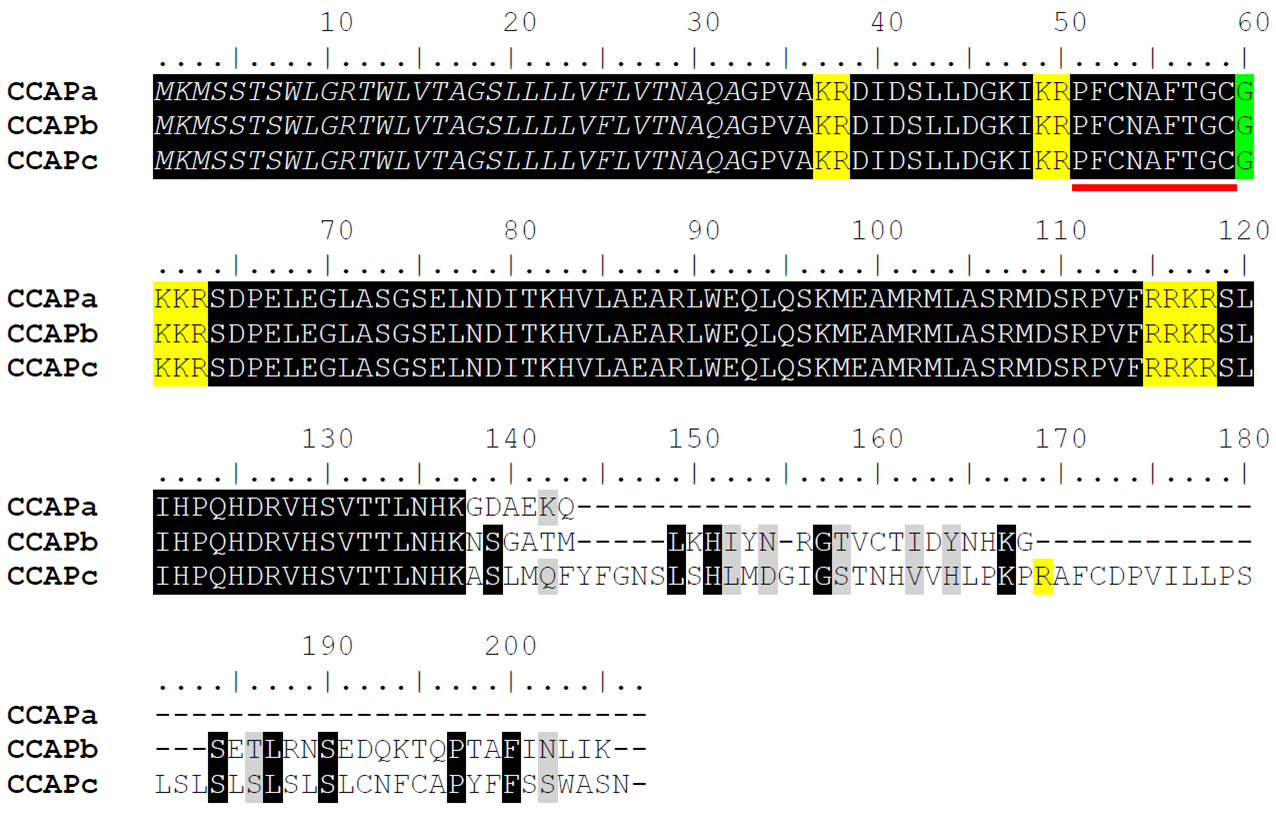


**Additional file 1: Figure S8**

Amino acid sequence alignment for crustacean cardioactive peptide (CCAP) transcript variants assembled in *Carma*_CNS-transcriptome. Predicted signal peptide sequences are shown in italics. Identical residues are highlighted black, conserved substitutions are highlighted grey, dibasic cleavage sites are highlighted yellow and amidation sites are highlighted green. The mature peptide is underlined red.


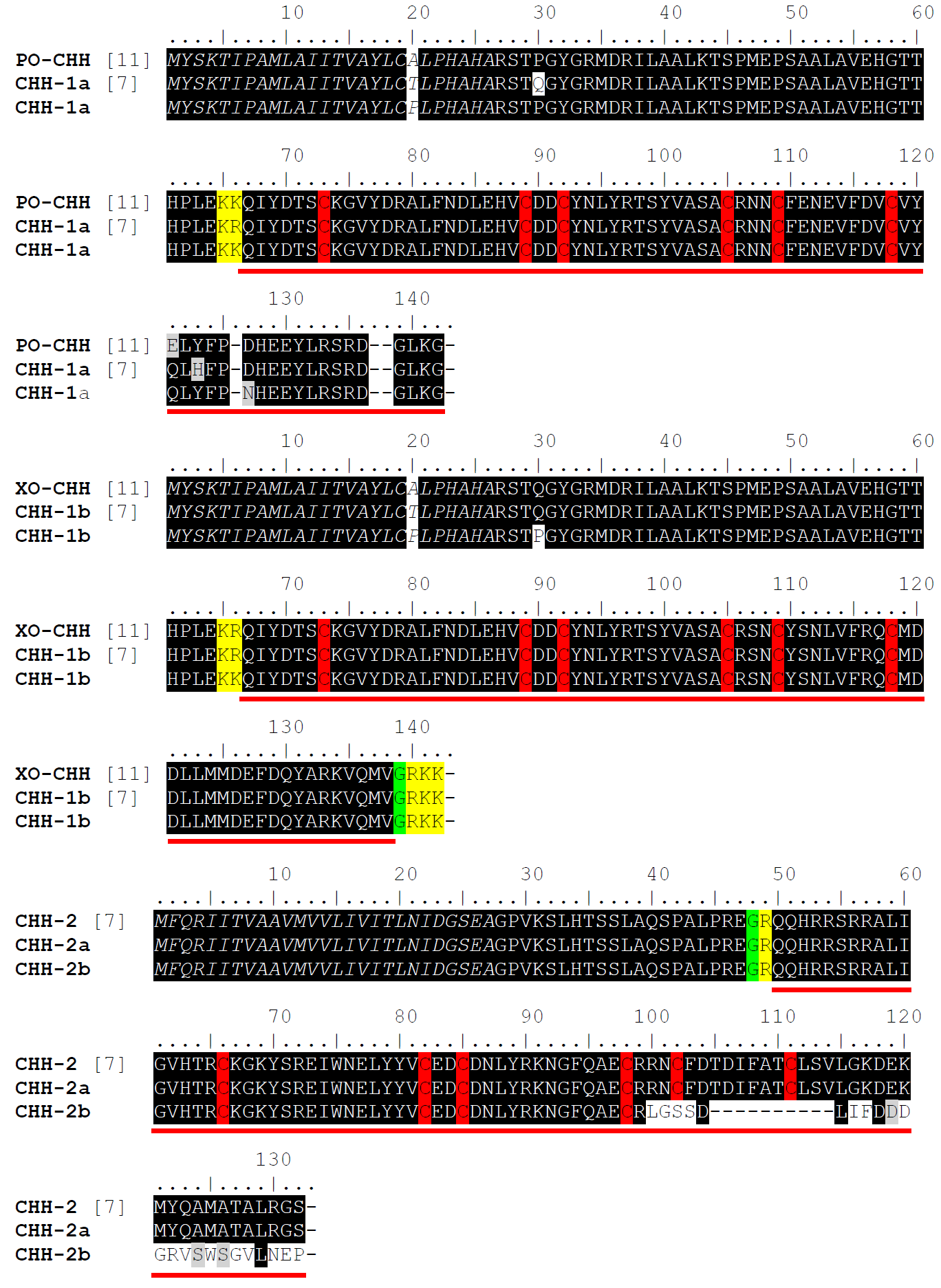


**Additional file 1: Figure S9**

Amino acid alignments for **A** CHH-1a and **B** CHH-1b splice variants for peptides sequenced from the X-organ and pericardial organs [11] and translated from nucleotide sequences generated by transcriptome assembly in this study and in Veenstra (2016) [7]. **C** Amino acid alignments for CHH-2a and CHH-2b splice variants for peptides translated from nucleotide sequences generated by transcriptome assembly in this study and in Veenstra (2016) [7]. Predicted signal peptide sequences are shown in italics. Cysteine residues are highlighted red, identical residues are highlighted black, conserved substitutions are highlighted grey, dibasic cleavage sites are highlighted yellow and amidation sites are highlighted green. The mature peptide is underlined red.


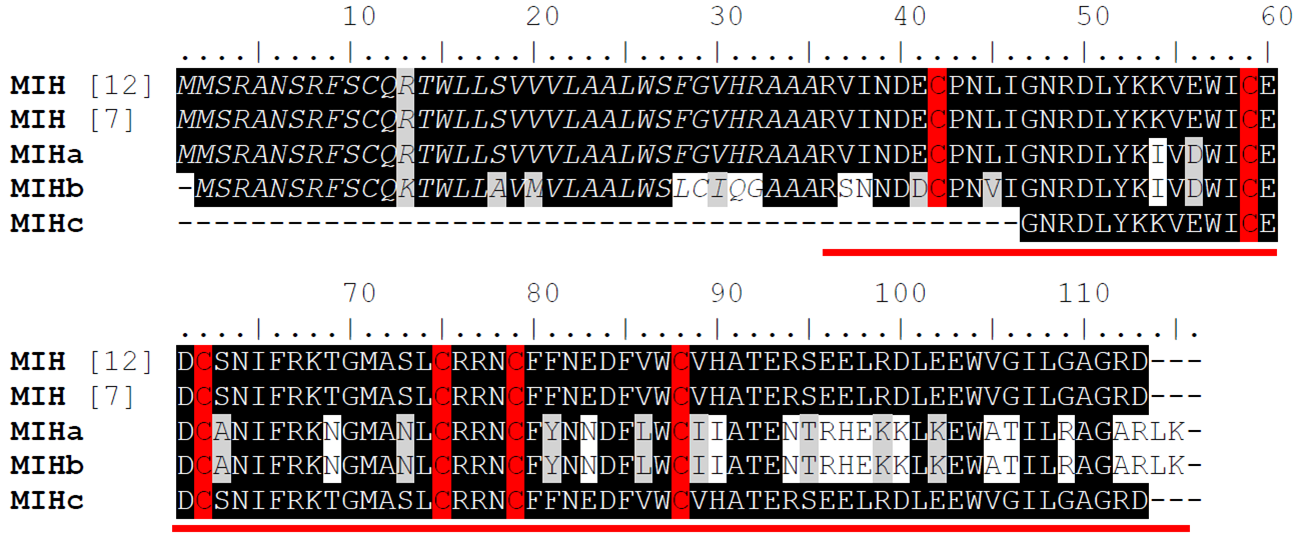


**Additional file 1: Figure S10**

Amino acid alignment for moult inhibiting hormone (MIH) peptide sequences identified by Klein [12] and translated from nucleotide sequences generated by transcriptome assembly in this study (MIH-a,b,c) and by Veenstra (2016) [7]. Predicted signal peptide sequences of MIH-a and MIH-b are shown in italics (MIH-c is a C-terminal partial sequence). Cysteine residues are highlighted red, identical residues are highlighted black, and conserved substitutions are highlighted grey. The mature peptide is underlined red.


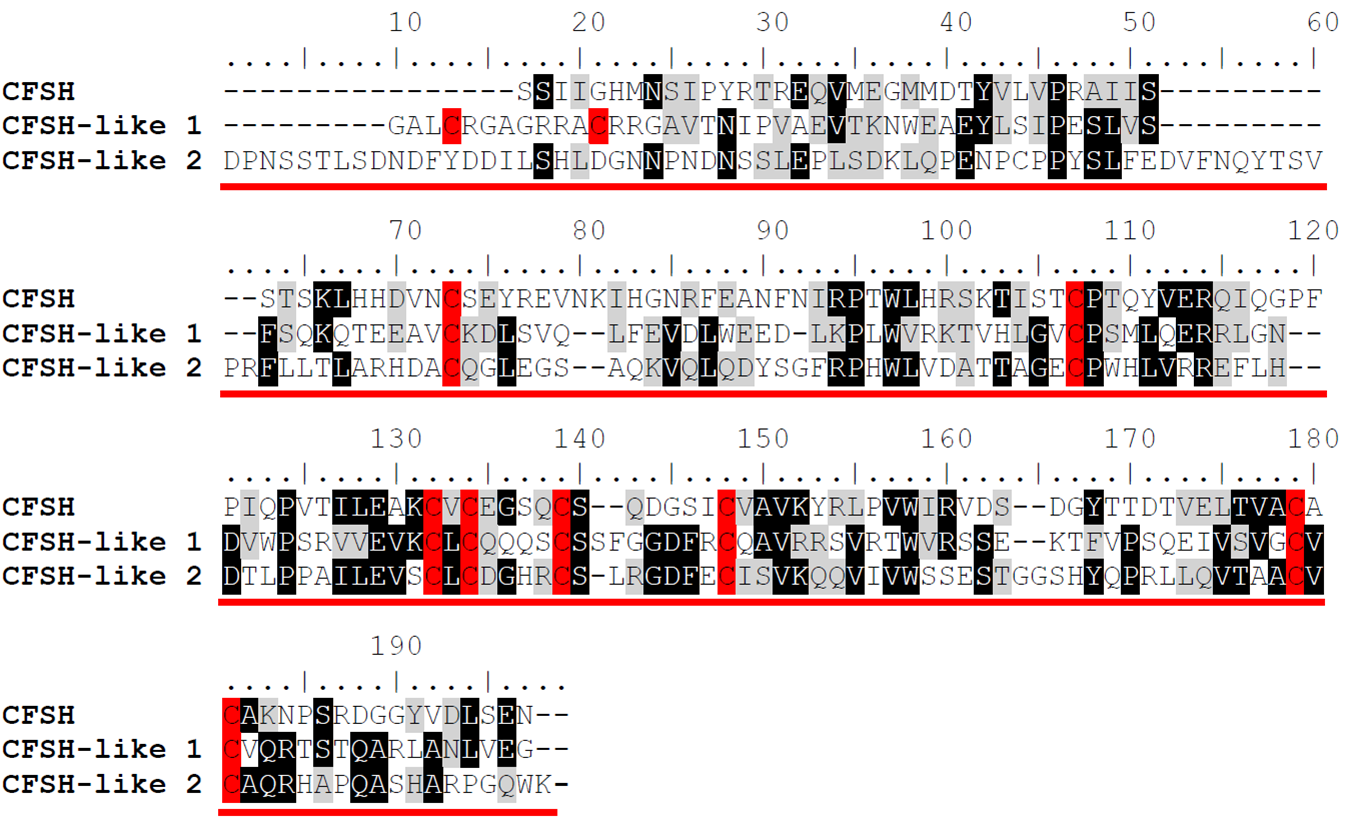


**Additional file 1: Figure S11**

Amino acid alignment of deduced peptide sequences of crustacean female sex hormone (CFSH) and CFSH-like neuropeptides from the *C. maenas* central nervous system, translated from *de novo* assembled nucleotide sequences. Cysteine residues are highlighted red, identical residues are highlighted black, conserved substitutions are highlighted grey, dibasic cleavage sites are highlighted yellow and amidation sites are highlighted green. The mature peptide is underlined red.

**Additional file 1: Table S8**

Differential expression statistics for GPCRs within *Carcinus maenas* CNS.

| **Annotation** | **Cluster** | **F** | ***P*** | **FDR** |
| --- | --- | --- | --- | --- |
| **Adhesion G-protein receptor-like** | Cluster-569.4176 | 9.47 | 0.0001 | 0.0071 |
| **Probable G-protein receptor Mth-like** | Cluster-119148.0 | 8.74 | 0.0002 | 0.0098 |
| **Xenotropic & polytropic retrovirus receptor 1-like** | Cluster-569.45616 | 8.51 | 0.0002 | 0.0109 |
| **Frizzled-2-like** | Cluster-569.40579 | 8.24 | 0.0003 | 0.0126 |
| **FMRFamide receptor-like** | Cluster-569.17428 | 7.37 | 0.0017 | 0.0414 |
| **G-protein coupled receptor 161-like** | Cluster-173119.0 | 5.80 | 0.0021 | 0.0481 |
| **G-protein coupled receptor Mth2-like** | Cluster-141714.0 | 5.80 | 0.0021 | 0.0481 |
| **G-protein coupled receptor MOODY-like** | Cluster-169726.5 | 5.76 | 0.0022 | 0.0495 |

Differential expression analysis statistics for clusters annotated ‘GO:0004930 G-protein coupled receptor activity’ and differentially expressed across the moult cycle in the CNS of *C. maenas.* The *P*-value should be read in conjunction with the false discovery rate (FDR).

**Additional file 1: Figure S12**

Differentially expressed genes annotated ‘GO:0004930 G-protein coupled receptor activity’ within *Carma*_CNS-transcriptome.

**Additional file 1: Table S9**

Differential expression statistics for GPCRs within *Carcinus maenas* YO.

| **Annotation** | **Cluster** | **F** | ***P*** | **FDR** |
| --- | --- | --- | --- | --- |
| **Probable G-protein coupled receptor B0563.6** | Cluster-90511.0 | 19.17 | 0.0000 | 0.0001 |
| **Probable G-protein coupled receptor Mth-like 3** | Cluster-144315.1 | 17.97 | 0.0000 | 0.0002 |
| **Probable G-protein coupled receptor 158** | Cluster-89342.3 | 10.15 | 0.0001 | 0.0017 |
| **Probable G-protein coupled receptor 158** | Cluster-89342.2 | 9.98 | 0.0001 | 0.0018 |
| **Frizzled-7-B** | Cluster-136339.9108 | 8.92 | 0.0001 | 0.0029 |
| **Rhodopsin, G0-coupled** | Cluster-62481.4 | 8.31 | 0.0002 | 0.0040 |
| **G-protein coupled receptor Mth2** | Cluster-82781.0 | 7.51 | 0.0005 | 0.0060 |
| **Metabotropic glutamate receptor 4** | Cluster-26393.0 | 6.82 | 0.0008 | 0.0087 |
| **Frizzled-1** | Cluster-112087.0 | 6.76 | 0.0009 | 0.0090 |
| **G-protein coupled receptor Mth2** | Cluster-136339.34388 | 6.20 | 0.0014 | 0.0125 |
| **Probable G-protein coupled receptor Mth-like 4** | Cluster-136339.38316 | 5.94 | 0.0018 | 0.0146 |
| **G-protein coupled receptor Mth2** | Cluster-136339.22266 | 5.59 | 0.0025 | 0.0183 |
| **G-protein coupled receptor Mth2** | Cluster-51079.0 | 5.14 | 0.0039 | 0.0242 |
| **Probable G-protein coupled receptor B0563.6** | Cluster-100561.0 | 5.06 | 0.0042 | 0.0256 |
| **Metabotropic glutamate receptor 2** | Cluster-141047.2 | 5.00 | 0.0045 | 0.0266 |
| **G-protein coupled receptor Mth2** | Cluster-136339.35182 | 4.76 | 0.0057 | 0.0314 |

Differential gene expression analysis statistics for clusters annotated with ‘GO:0004930 G-protein coupled receptor activity’ and differentially expressed across the moult cycle in the YO of *C. maenas.* The *P*-value should be read in conjunction with the false discovery rate (FDR).

**Additional file 1: Figure S13**

Differentially expressed clusters annotated ‘GO:0004930 G-protein coupled receptor activity’ within *Carma*_YO-transcriptome.

**References**

1. Smith-Unna R, Boursnell C, Patro R, Hibberd JM, Kelly S. TransRate: reference-free quality assessment of de novo transcriptome assemblies. Genome Res. 2016;26:1134–44.

2. Grabherr MG, Haas BJ, Yassour M, Levin JZ, Thompson DA, Amit I, et al. Trinity: reconstructing a full-length transcriptome without a genome from RNA-Seq data. Nat Biotechnol. 2011;29:644–52.

3. Liu J, Li G, Chang Z, Yu T, Liu B, McMullen R, et al. BinPacker: packing-based *de* *novo* transcriptome assembly from RNA-seq data. PLoS Comput Biol. 2016;12:e1004772.

4. Peng Y, Leung HCM, Yiu S-M, Lv M-J, Zhu X-G, Chin FYL. IDBA-tran: a more robust *de* *novo* de Bruijn graph assembler for transcriptomes with uneven expression levels. Bioinformatics. 2013;29:i326-34.

5. Schulz MH, Zerbino DR, Vingron M, Birney E. Oases: robust *de* *novo* RNA-seq assembly across the dynamic range of expression levels. Bioinformatics. 2012;28:1086–92.

6. Zerbino DR, Birney E. Velvet: algorithms for *de* *novo* short read assembly using de Bruijn graphs. Genome Res. 2008;18:821–9.

7. Veenstra JA. Similarities between decapod and insect neuropeptidomes. PeerJ. 2016;4:e2043.

8. Oliveros JC. VENNY. An interactive tool for comparing lists with Venn’s diagram. 2007;http://bioinfogp.cnb.csic.es/tools/venny/index.html.

9. . Hall TA. BioEdit: a user-friendly biologicl sequence alignment editor and analysis program for Windows 95/98/NT. Nucleic Acid Symp Ser. 1999;41:95–8.

10. O’Grady JF, Hoelters LS, Swain MT, Wilcockson DC. Identification and temporal expression of putative circadian clock transcripts in the amphipod crustacean *Talitrus saltator*. PeerJ. 2016;4:e2555.

11. Dircksen H, Böcking D, Heyn U, Mandel C, Chung JS, Baggerman G, et al. Crustacean hyperglycaemic hormone (CHH)-like peptides and CHH-precursor-related peptides from pericardial organ neurosecretory cells in the shore crab, Carcinus maenas, are putatively spliced and modified products of multiple genes. Biochem. J. 2001;356:159–70.

12. Klein JM, Mangerich S, De Kleijn DPV, Keller R, Weidemann W. Molecular cloning of crustacean putative molt-inhibiting hormone (MIH) precursor. FEBS J. 1993;334:139–42.
