# Supplementary material for: Transcriptomic analysis of crustacean neuropeptide signaling during the moult cycle in the green shore crab, Carcinus maenas
Source: BMC Genomics. 2018 Sep 26;19:711. doi: 10.1186/s12864-018-5057-3 (PMC6158917; doi:10.1186/s12864-018-5057-3)
Supplement: Supplementary file 3 — Neuropeptide prepro-hormone processing and deduced mature neuropeptide and precursor-related peptides. (DOCX 88 kb) [file 12864_2018_5057_MOESM3_ESM.docx]

**Neuropeptide prepro-hormone processing**

Predicted signal peptides in grey italics, predicted mature peptides are in bold, putative convertase cleavage sites and basic amino acid residues predicted to be removed by carboxypeptidase are highlighted yellow, Gly (G) amidation sites are highlighted green, and cysteine residues are highlighted red. N-terminal extensions upstream of the predicted signal peptide in red text.

**Adipokinetic hormone-Corazonin-like peptide (ACP)**

*MASWLAVALVVSCVLMDSVTP***QITFSRSWVPQ**GKRSSPPPDIPEALDPCKDARVATITSLAGHLLDMMNEAAPADHRPLSDDTTPVLRLRNALMDRRRRMA-

*+VVSCVLMDSVTP***QITFSRSWVPQ**GKRSSLPADIPEALDPCKDARVATITSLAGHLLDMMNEAAPADHRPLSEDTTPALRLRNAMMDRRRRMA-

**Agatoxin-like peptide (ALP)**

*MGSKTAVMVLALSLLVSVVLA*QPLLEEGREPDGMQQAEVDYTADLLDHLLGRAQKRSPDIYLFRRSCIRR**GGACDHRPNDCCYNSSCRCNLWGTNCRCQRMGLFQQW**GK-

*+HNASPSPLSLALTLTLPVGR*EPDGMQQAEVDYTADLLDHLLGRAQKRSCIRR**GGACDHRPNDCCYNSSCRCNLWGTNCRCQRMGLFQQW**GK-

**Allatostatin-A**

*+VVVVVVVVAGLPHPAKA*QLDEYDLQYDPNTLLQILQQYEAVAAARADEEQQDEDDEFGFGKR**HTPPRHSYAFGL**GKR**APQPYAFGL**GKR**GGPYSYGL**GKK**SDMYSFGL**GKK**SGSYNFGL**GKRSVRDVASEEEEEVKEEEEEEERQQELRKEEEKEAGGKRSKRDVSEEEEEENNMSTTSQYDTYNKR**PRAYSFGL**GKRKQDDVGFSKR**PRGYAFGL**GKREYDDMYTEKR**PKVYAFGL**GKRGDDDADMEKR**QGAYSFGL**GKRGDDAEMEKR**PKVYSFGL**GKRGDDDVDLEKR**LKAYSFGL**GKRADDSDFDKR**QGPYAFGL**GKR**GYEDEDEDRPFYALGL**GKR**PRTYSFGL**GKRTSDDDTEEEEEEEEEDEDEEEEEATNYKGKR-

+GLGKR**EPYEFGL**GKR**EPYAFGL**GKR**EPYAFGL**GKR**ASPYAFGL**GKR**GGPYAFGL**GKR**APTDMYSFGL**GKR**DPYAFGL**GKR**EAYAFGL**GKR**PADLYEFGL**GKR**AGPYAFGL**GKR**AAPYAFGL**GKR**PDMYAFGL**GKR**ASPYAFGL**GKR**ATGQYAFGL**GKK**SGQYSFGL**GKRAQGDAGDSYTLGRR**SGSYSFGL**GKR**AGPYSFGL**GKREAMDEEDAHTKMTTGNKASIDQSSSSSSSSSSSHSS-

+GLGKR**EPYEFGL**GKR**EPYAFGL**GKR**ASPYAFGL**GKR**GGPYAFGL**GKR**APTDMYSFGL**GKR**DPYAFGL**GKR**EAYAFGL**GKR**PADLYEFGL**GKR**AGPYAFGL**GKR**AAPYAFGL**GKR**PDMYAFGL**GKR**ASPYAFGL**GKR**ATGQYAFGL**GKK**SGQYSFGL**GKRAQGDAGDSYTLGRR**SGSYSFGL**GKR**AGPYSFGL**GKREAMDEEDAHTKMTTGNKASIDQSSSSSSSSSSSHSS-

**Allatostatin-B/Myoinhibitory peptide (MIP)**

*MQLATLTATLLTLMAAAAA*QDEGSGLAVAQAKR**AGWSSMRGAW**GKRDDSSDHGLQISEDKR**NNNWSKFQGSW**GKRGEEIQDAEEKR**GNWNKFQGSW**GKRANDMTEEAALQAAEDKR**AGWNKFQGSW**GKRGDEITSEDDLQDAEDKR**TSWGKFQGSW**GKRQDDLIQPQDLEDKR**NNWSKFQGSW**GKR**AGWSSLQGAW**GKR**AWSNLQGAW**GKRSPNDSEDIEDEALEEEELQVSPEVLARMVAAAPVKRGWA+

*MQLATLTATLLTLMAAAAA*QDEGSGLAVAQAKR**AGWSSMRGAW**GKRDDSSDHGLQISEDKR**NNNWSKFQGSW**GKRGEEIQDAEEKR**GNWNKFQGSW**GKRANDMTEEAALQVSRGIGGAGGRLMKV-

+SEDIEDEALEEEELQVSPEVLARMVAAAPVKRGWALW*G*KR**PDYPAVSPRSTNWSSLRGTW**GKR**SGNWSNLRGAW**GKR**VPNDWAHFRGSW**GKRNPDTLISA-

**Allatostatin-C**

*MMPCPGHLIVALTLVLALTHA*LPAKEGPEAQKEVSSGHDGGRLTKRAAPASDTSQEELAALKDLILSRLAAELEDSYQDLPSFKRDLLKAGIDMQEDDEGDDEGRREVKKKRMFAPLSGLPGNLRTIKR**QIRYHQCYFNPISCF**RRK-

+DLKAEGLSTLTTTTTTTAILPFSSIIVNVILLMTLLAPHVSPAHLLMSPRPHYIEEIRPVLPTNIPLMIRQQQQQQQPILAAPLQVQQEPRD+

+IEEIRPVLPTNIPLMIRQQQQQQQPILAAPLQVQQEPRKRAAIVLDKLMFALQKALDEPSSPPHPQAAPLPPRTPYTGPVDLQRR**GNGDGRLYWRCYFNAVSCF**-

*MVFPRHCAVLTLAMVVLAASGTTSA*KPLGDLDPSAGGPPPFSARQAQVYEPYGNNVEEDGSLDAALINYLFAKQLVQRLRNPSEVSRENQRKR**SYWKQCAFNAVSCF**GKRK-

**Bursicon-α**

*MMSSLPWTVVGAAVTVLVVIGVGVAQA***DECSLRPVIHILSYPGCTSKPIPSFACQGRCTSYVQVSGSKLWQTERSCMCCQESGEREAAITLNCPKPRPGEPKEKKVLTRAPIDCMCRPCTDVEEGTVLAQEIANFIQDSPMDSVPFL**K-

**Bursicon-β**

*MWWCGVLMVVTAVVVVVPDATQA***RSYGVECETLPSTIHISKEEYDDTGRLVRVCEEDVAVNKCEGACVSKVQPSVNTPSGFLKDCRCCREVHLRARDITLTHCYDGDGARLSGAKATQHVKLREPADCQCFKCGDST**R-

**Calcitonin**

*MRLVVIVLCLMLLWCVGVGA*QPTHHESQEAYLSEKMREYLLLRRLLISVLSERQPPPMEAPRKR**TCYLNGGLSHGCDYQDLVSSTVEKNYWDSVNSP**GKKRKRRRRRRMAEEEEEI-

*MRLVVIVLCLMLLWCVGVGA*QPTHHESQEAYLSEKMREYLLLRRLLISVLSERQPPPMEAPRKR**LCLNLGDPSCYEGNMAANGDDNNYLIGQNNP**GK-

**CCHamide**

*MQTCLRISTLLLLLLPCLLLLTPAVEA***HGSVKLGCLNYGHSCLGAH**GKRGSWSPSVPRPPAAALLAPFLQSLAAPRSPASSWQDKIMAQHRQHKNEQQQQQQQKQQQQQQQQQQQQ+

**+CFGAH**GKRAGDQYGNLDPADLYPPTNQLAEVDEARYPLDEAPVTNPRTLAKVRDLMDFMGHLLRQRAAPLQQQQQQQRAPLAAQLGPNDAYLH-

**CCRFamide**

*MAWCVGWLLVGVWAWSVGG***APRPSLPRPGCDVEALRCDVICSVPDLGFHCTRCARRPMRF**GKRGGHVDPPLPSISSSSSVMPGGGG+

**CNMamide**

*MVSRGQQSSVSLTWLLVGAVSLVLVARVQP*YPGYVDRLPSLRRYPQTQQQQQALGEGEPFAGANDDYVEDDDDLGYSKKYAYNTLMAGREPDIFPYDQAEASNKNLQQILRSFRPVVGGTYPSDRVLPWDDLHELGLKDGRSAEEQLSAPDTSLTQLSFAKHRQKR**VMCHFKICNM**GRRRRARHSNPLQGWLS-

**Corazonin**

*MVRRVTVLVLVASLALAAC***QTFQYSRGWTN**GRKRSNDLGTVVGLGSGRRTGVNLLPAEARRSLQQQTTPHRSLPRDVEERLRAVEAGVSALLHAAQQNPEAPPATPGEQDYYAQN-

**CRF-like diuretic hormone (DH44)**

+NNNNNNNSLTPSTTTTTTNSGTSTTANNPRAIPHSAPMFKRAWQHSFSRRR**NTGLSLSIDASMKVLREALYLEIARKKQRQQLQRAQHNKALLNTI**GKRDVASQLVGVDQSVSLDNDRN-

**Crustacean cardioactive peptide (CCAP)**

*MKMSSTSWLGRTWLVTAGSLLLLVFLVTNAQA*GPVAKRDIDSLLDGKIKR**PFCNAFTGC**GKKRSDPELEGLASGSELNDITKHVLAEARLWEQLQSKMEAMRMLASRMDSRPVFRRKRSLIHPQHDRVHSVTTLNHKGDAEKQ-

*MKMSSTSWLGRTWLVTAGSLLLLVFLVTNAQA*GPVAKRDIDSLLDGKIKR**PFCNAFTGC**GKKRSDPELEGLASGSELNDITKHVLAEARLWEQLQSKMEAMRMLASRMDSRPVFRRKRSLIHPQHDRVHSVTTLNHKNSGATMLKHIYNRGTVCTIDYNHKGSETLRNSEDQKTQPTAFINLIK-

*MKMSSTSWLGRTWLVTAGSLLLLVFLVTNAQA*GPVAKRDIDSLLDGKIKR**PFCNAFTGC**GKKRSDPELEGLASGSELNDITKHVLAEARLWEQLQSKMEAMRMLASRMDSRPVFRRKRSLIHPQHDRVHSVTTLNHKASLMQFYFGNSLSHLMDGIGSTNHVVHLPKPRAFCDPVILLPSLSLSLSLSLSLCNFCAPYFFSSWASN-

**Crustacean female sex hormone (CFSH)**

*MKQNGTSLRLTSFILFLFLECSVA*YTPVYPSRVQHFLQYLQQERQEDGLPLTTQSPKR**SSIIGHMNSIPYRTREQVMEGMMDTYVLVPRAIISSTSKLHHDVNCSEYREVNKIHGNRFEANFNIRPTWLHRSKTISTCPTQYVERQIQGPFPIQPVTILEAKCVCEGSQCSQDGSICVAVKYRLPVWIRVDSDGYTTDTVELTVACACAKNPSRDGGYVDLSEN**-

**CFSH-like**

MDYTIAAATKIT*MWRGLGLHLCLGLVLTTYPIFG*TAVSNALGSREGAVQQEQQEQQVAPFVVPQWWWLSSLLSLPHGHHGYTTPADAAEAAAAARKVAGDTEESPVSEDEASTLSLMLPLEQLTDGKESVSSPSVAKR**GALCRGAGRRACRRGAVTNIPVAEVTKNWEAEYLSIPESLVSFSQKQTEEAVCKDLSVQLFEVDLWEEDLKPLWVRKTVHLGVCPSMLQERRLGNDVWPSRVVEVKCLCQQQSCSSFGGDFRCQAVRRSVRTWVRSSEKTFVPSQEIVSVGCVCVQRTSTQARLANLVEG**-

*MKISPSAELRSTIALVLLLAAMSTWS***DPNSSTLSDNDFYDDILSHLDGNNPNDNSSLEPLSDKLQPENPCPPYSLFEDVFNQYTSVPRFLLTLARHDACQGLEGSAQKVQLQDYSGFRPHWLVDATTAGECPWHLVRREFLHDTLPPAILEVSCLCDGHRCSLRGDFECISVKQQVIVWSSESTGGSHYQPRLLQVTAACVCAQRHAPQASHARPGQW**K-

**Crustacean hyperglycemic hormone (CHH)**

*MYSKTIPAMLAIITVAYLCPLPHAHA*RSTPGYGRMDRILAALKTSPMEPSAALAVEHGTTHPLEKK**QIYDTSCKGVYDRALFNDLEHVCDDCYNLYRTSYVASACRNNCFENEVFDVCVYQLYFPNHEEYLRSRDGLKG**-

*MYSKTIPAMLAIITVAYLCPLPHAHA*RSTPGYGRMDRILAALKTSPMEPSAALAVEHGTTHPLEKK**QIYDTSCKGVYDRALFNDLEHVCDDCYNLYRTSYVASACRSNCYSNLVFRQCMDDLLMMDEFDQYARKVQMV**GRKK-

*MFQRIITVAAVMVVLIVITLNIDGSEA*GPVKSLHTSSLAQSPALPREGR**QQHRRSRRALIGVHTRCKGKYSREIWNELYYVCEDCDNLYRKNGFQAECRRNCFDTDIFATCLSVLGKDEKMYQAMATALRGS**-

*MFQRIITVAAVMVVLIVITLNIDGSEA*GPVKSLHTSSLAQSPALPREGR**QQHRRSRRALIGVHTRCKGKYSREIWNELYYVCEDCDNLYRKNGFQAECRLGSSDLIFDDDGRVSWSGVLNEP**-

**CHH/MIH-like**

*MVGKSPSLSVVACWVILLAGSLLMQA***SPTTSSSSFKDKSYQFRLWPGTQREFEFYHCGGEFDAASRKLYSHLSAVCEDCKNYYRSEPLLRQRCMSKCFDNEIFTYCAETLYSIDKFKDYEEMKVQIKA**-

**Diuretic Hormone 31 (DH31)**

*MNNLGVVFVTLVATVVLLSSVNA*SPLNREPSRAVVEIDDPDYVLELLTRFSNSIIRAKELEKFVRSSSGTKR**GLDMGLGRGFSGSQAAKHLMGLAAANYAGGP**GRRKRDSQATTLDLHHDDQHLAQHEHAAAAVAAAGLPQHSSR-

**Eclosion hormone**

**+LAAVTDAHADEMYGDYFHGQACAESCIRTQGMTIPDCNNPATFNRFL**KRFI-

*MVGSRKVGVSALLVLSVALVLSVVLLPPSASA***AVAANRKVSICIKNCGQCKKMYTDYFNGGLCGDFCLRTKGRFIPDCNRPDILIPFFLQRLE**-

**Carcikinin/Ecdysis Triggering Hormone (ETH)**

*MAWLVAATVLAAVVSVARA***DAGHFFAETPKHLPRI**GRRGDLPPLTTLLSEEDARSSSAGTQSMTEALAGLDSDGDGCIGIGELLRIPAVRVALLLQNPALLTPANPATPEHDGHATEDTFTSDRRPEPRLLRYLQK-

*MAWLVAATVLAAVVSVARA***DAGHFFAETPKHLPRI**GRRGDLPPLVSERVDVLRSDVLGIVW-

MFSVLDSRAG*MAWLVAATVLAAVVSVARA***DAGHFFAETPKHLPRI**GRRGDLPPL+

**ELFamide**

*MVQGTPCLTKCIVVLSCVSCVFT*ALQTTHPDESVSDVSKR**AGRDPMLRYLLVAMAQPGPRYAAPQILSRGVRRMGSEFL**GKRSVAVSPESRKMCESGSCVTKEDQHHTDDLRKEQMSFTGQYNEQNLDDDHEDGMDKR**AIGSLPRSHLARYFSLLMNRKMGSEFL**GKR**AMGSEFL**GKR**AMGSEFL**GKR**AMGSEFL**GKR**AMGSEFL**GKR**AMGSEFL**GKRAMGSEFL+

**Elevenin-like peptide**

MKAADPAHRRPPRLLLVSYGRTEDQTTHTPPEPSPRTTVVLHICGSSLCLLACTPGQPPKSPLPTFVLLTT*MTSTAPRISLVLHTLLLLTALASFASCGHA***IDCRRFVFAPQCRGIIA**KRTVSDAAIGLPHVLQEQRQWPESIPVDYVVPEYSPVYQRVRSTYTRPNAASRDVSVGAQFPSPNAAMTEMDVRESGDMELLSPYVKVIHRSENLPYQRK-

**FMRFamide**

*MIAVAWVLLSGVAWCLA*SPLNTVPGAIEASPSTHNDPGSDVPEIPQEKRLLKYFLPSGSSWMSTQQEGSKR**GISRNYLRF**GRGEEDKR**GGRNFLRF**GRGDSSSIEDVEVLPETEDSIEKR**NRNFLRF**GR**DRNFLRF**GRSDAEEFSLPGGPLAFTNVHEQDLEEYPTEEEKR**AAHKNYLRF**GR**GNRNFLRF**GR**DGNRNFLRF**GRSVDRQLNEQKARDAPLAPTTVPHSSAKTQESHRSKRSATPYNYVVLPSHGPAAWSQDFQPDQDEDLEEAIEGPESAMTKR**GYNRSFLRF**GR**DRNFLRF**GKRNDESDPQMVMMEPASYPRYVR**APQGNFLRF**G-

**Glycoprotein-A2 (GPA2)**

*MVRAVLLLVACLLASSSS***HQHTWQTPGCHKVGHTRRISIPECVEFDITTNACRGYCESWSVPSAWQTLVYNPHQVVTSIGQCCNIMETEDVKVKVMCVEGPRELIFKSASTCDCFHCKKY**-

**Glycoprotein-B5 (GPB5)**

*MAGRAGDMGGVSGRRGGGGVCGRLMAVLAAALALLLFLLPPSTA***IDPQSTLECHRRQYSYKVHKTDDDGRMCWDVVNVMSCWGRCDSNEIADWKFPYKRSHHPVCIHDETQITEVTLRNCDEDVAPGTELYSYHEATRCACSVCKSSQASCEGLRYRGARRAPRAQVPR**G-

**HIGSLYamide**

+MFYRQDKRHYGSLLRNGPLPFMQEDKRHFGSLLKSPRYRAISIPGKRDSPAVDEQQEDTVRQLEEARELSKQQFSSLLRNQFDDDLELQQRLLAAGLISPRDTQELAALHSTWNPATSPGQDKR**HIGSLYR**GKKDDATLYDLSEEKR**HIGSLYR**GKKDDDSLYDLSEDKR**HIGSLYR**GKKDEDSLYDLSEDKR**HIGSLYR**GKKDEDSLYHELSEDKR**HIGSLYR**GKKDEDNDLGLYEDKR**HIGSLYR**GKKRRR-

**+LYR**GKK^DDDDAQALSEDKR^**HIGSLYR**GKK^DENNLNQDLSEDKR^**HVGSLYR**GKK^DEDNSQGLFEDKR^**HVGSLYR**GKK^DEDSTQNLAEDKR^**HIGSLYR**GKK^DEDNSQTLAEDKR^**HVGSLYR**GKK^DEDNSQNLSEDKR^**HIGSLYR**GK+

**+SLYR**GKKDEDNSQNLSEDKR**HIGSLYR**GKKDEDNTNNLSEDKR**HIGSLYR**GKKDEDNSPNLSEDKR**HIGSLYR**GKKDEDNSQNLSEDKR**HIGSLYR**GKK+

**+YR**GKKDEDNSPNLSEDKR**HIGSLYR**GKKDGGNTSTFSEDKR**HIGSLYR**GKKDDDTNSLSEDKR**HIGSLYR**GKKDEDNANDLSEDKR**HIGSLYR**GKKD+

+NSISEDKR**HIGSLYR**GKKDEDNAHDLSEDKR**HIGSLYR**GKKHQQDFPDLIQDEEAIERRLHELPDKRYFASLLRNRPSAFGPWNPPDKRHIGSFFQNRQVPFGMQDSTEKRHIGSFLQHRPSAFVPDKRHIGSFLQNRQLPFASHDQSAEKRHIGSFFQNRQSGFGTQDLSADKRHVGSFFQNRQSPLGLQDSAEKRHISSFMQDQGPPFAPDQGSGLRRRKRYIGALARTNNMPSPYSRARQNRRDAPDPSSLRAHLLQRLLNMERQRDFLRQRQQQALITRTQNAAMMLKYLEHAGLDGEDVALDLQPEQDLGVDLALDEHREPGGDLDHLQYTMGGATPLEDNALTYHSANKRFLGSLARSGWFPRRYSGAYSFDLQKRDLASQMTLDEDDEDEEEYEEENKPFFTFSY-

+NSISEDKR**HIGSLYR**GKKDEDNAHDLSEDKR**HIGSLYR**GKKHQQDFPDLIQDEEAIERRLHELPDKRYFASLLRNRPSAFGPWNPPDKRHIGSFFQNRQVPFGMQDSTEKRHIGSFLQHRPSAFVPDKRHIGSFLQNRQLPFASHDQSAEKRHIGSFFQNRQSGFGTQDLSADKRHVGSFFQNRQSPLGLQDSAEKRHISSFMQDQGPPFAPDQGSGLRRRKRYIGALARTNNMPSPYSRARQNRRDAPDPSSLRAHLLQRLLNMERQRDFLRQRQQQALITRTQNAAMMLKYLEHAGLDGEDVALDLQPEQDLGVDLALDEHREPGGDLDHLQYTMGGATPLEDNALTYHSANKRFLGE-

**Hyrg**

*MNIFNILLLVIAAVVSVMA***QQKPRILLEDPTNLQNQPWVQPPRFHYRGFNRPQVGAGWP**-

**Insulin-like**

MTLCSLRHRCKLVPQSGTLHRSG*MKVQRQVLLLVMVTAMHAGITRS***SPRTLPGQGLVTDGERRLCGWRLANELNRVCKGVYNKPTVSNNALFYLRGRGV**KRVDLMPLGMELEFSPWTQASADDLRSDQISESHTSHIPKQLPFLAEAEASRVVGGLPRVKR**GLSAECCRKACSVSELAGYCY**-

**Inotocin/Vasopressin-like**

*MQSGVAVTVVVTLLVGSAA***SCFITNCPPG**GKRSGGLMSTLGRARTCASCGPGLLGRCLGPDICCGARIGCFLGSRETRMCRTENMVPITCYNSDLKPCGRMKEGLCAAPGLCCTENKCEMNDDCVVEDMRGEEVAASQRGARSRLDLLTAARDRWEEQ-

**Leucokinin**

*MGTRCWGAEVWLVVGVAVIIASSPSALA*QEIPSDQDSSLLPCSLASPDESLRCEVKRGQRFTWIGHPPSPSSLRDVYMRLVRHGRLPQNAILALDLKR**APFHAWA**GKRSSFTLPSPSVLHIGEEVPFSTLEEGDEEEEEQEEWTTLLDPTAKLLSLEGDEMASTLVPLLPPAMSHVRVRRSTEKQPNTLTNSYIEGSRKPR**FRSWG**GKRSGQERGKLEDVLRAWNTQGDGMAWSANMKVNADTKRK**AFSAWA**GKRSYDIKR**GGGFSAWA**GKREAFDSWNGKR**SEEDERKQAFSAWA**GKRSDDEKRQAFNAWAAKR**QGFSAWP**GKRSDNE+

+**GFSAWA**GKRSDNENDNDKR**QGFSAWA**GKR**KFNAWA**GKRSYDDAFQEQKEEMEKSKGQLSSLVPQHQQQEHQQVSELLHHTPDSLTHWDANWDR-

**Moult-inhibiting hormone (MIH)**

*MMSRANSRFSCQRTWLLSVVVLAALWSFGVHRAAA***RVINDECPNLIGNRDLYKIVDWICEDCANIFRKNGMANLCRRNCFYNNDFLWCIIATENTRHEKKLKEWATILRAGARL**K-

*MSRANSRFSCQKTWLLAVMVLAALWSLCIQGAAA***RSNNDDCPNVIGNRDLYKIVDWICEDCANIFRKNGMANLCRRNCFYNNDFLWCIIATENTRHEKKLKEWATILRAGARL**K-

+**GNRDLYKKVEWICEDCSNIFRKTGMASLCRRNCFFNEDFVWCVHATERSEELRDLEEWVGILGAGRD**-

**Myosuppressin**

*MVFRLQPWCSLLLVGVVVVLGVCAGVGEA*IPPPICFNQKLVLTPYARRLCAALNDISKFSRAMEDYLDAQAIKNSMGVNEPEVKR**QDLDHVFLRF**GRAQQ-

**Natalisin**

*MAGLSGLVVSALAVTLAAT*AQEFSPGDGGSSELYRHSRSVEVGGDASWLPSLQDDQLTQVESRQDAGQGGTTFWVARGKKDADGSTSYYWGPNQGLWGEAFQRGAPAMSSSSRVPLLIRSGWESNPSLWGKR**DGRGPFWAAR**GKR**TNPFWISR**GRRDSSEASPSLLQQWMAEEPTMQNQKEERVWSGESKREEGGPFW+

+EASPSLLQQWMAEEPTMQNQKEERVWSGESKR**EEGGPFWISR**GKR**PQPGSAESQLASLWAIR**GRK**SGADNTFWVAR**GKK**ETNAQGPFWAAR**GKK**SGSDSETGPYWIAR**GKK**QDGTSSSNGPYWIAR**GKK**EDDGAVFWAAR**GKK**DSQTNGRSEDAAHSFWVAR**GKKNSHATSHSTEKDNDDDDDDDDDEDEHHDEATQKATDHHY+

**Neuroparsin 1**

*MASSCCRTATLILVCSCLLLLLQEASG***APRCDRHDEEAPKNCKYGTTQDWCKNGVCAKGPGETCGGYRWSEGKCGEGTFCSCGICGGCSPFDGKCGPTSIC**-

**Neuroparsin 2**

MIYVLNNVSLCLVFFFSFQTRQK*MEMSTRSCILFFIVSSTTLLFLLPGRCEG***GPICASLNEVLPEMLQAPCKHGVVMDWCGNARCAKGPGQTCGGRWNEHGTCGKGMYCVCGYCAGCSTDLQCALGRFC**-

MIYVLNNVSLCLVFFFSFQTRQK*MEMSTRSCILFFIVSSTTLLFLLPGRCEG***GPICASLNEVLPEMLQAPCKHGVVMDWCGNARCAKVRRFID**-

**Neuroparsin 3**

*MTHTTRPATLILASCLFLLLLLLQRGSA***APQCSSYDQEPPKNCKYGTALDWCRNGVCAKGPGESCGGYRREQGICGEGTYCECGRCRGCSPFDTSCHDAVLC**-

**Neuroparsin 4**

*MKNLMKAAELLIIVYLLFLSSNVSS***VPLCGNISDAELSQCKHGTVRNRCGKTVCAKVSNPDSLSSYTSLESVTRQKIIHQDF**-

*MKNLMKAAELLIIVYLLFLSSNVSS***VPLCGNISDAELSQCKHGTVRNRCGKTVCAKGLGEACMVYRWERDLCGIGTFCGCGFCQGCNTSLQCWEC**-

**Neuropeptide F**

*MCRQLLTALVVGVVLVGALEMGVAEA***KPDPTQLAAMADALKYLQELDKYYSQVSRPRF**GKRSEMVLPPGDALMEASERLLETLAKRR-

*MCRQLLTALVVGVVLVGALEMGVAEA***KPDPTQLAAMADALKYLQELDKYYSQVSRPRF**GKRSEMVLPPGDALVSVRG-

*MCRQLLTALVVGVVLVGALEMGVAEA***KPDPTQLAAMADALKYLQELDKYYSQVSRPSPRSAPGPASQIQALEKTLKFLQLQELGKMYSHMARPRF**GKRSEMVLPPGDALMEASERLLETLAKRR-

*MCRQLLTALVVGVVLVGALEMGVAEA***KPDPTQLAAMADALKYLQELDKYYSQVSRPSPRSAPGPASQIQALEKTLKFLQLQELGKMYSHMARPRF**GKRSEMVLPPGDALVSVRG-

*MRGSLMVVAVVATVAVGWA***AQLPSRQDGSALEALQALHEAALAGTLSSAEVQYPSRPNVFKSPGELRRYLDALNAYFAIAGRPRF**GKRGKQVRQPEELYDY-

**Orcokinin-A**

*MTRDVFCTALLLALSVMASEA*AIKDAPAHPSNHPDTGYASDGSSAKRFDAFTTGFGHSKR**NFDEIDRSSFGFN**KR**NFDEIDRSGFGFA**KR**NFDEIDRSGFGFA**KR**NFDEIDRSSFGFN**KR**NFDEIDRSSFGFV**KRMLTPRDLANLYKR**NFDEIDRSGFGFV**RRNAE-

**Orcokinin-like**

+EGSDFGLRKR**ALDSIDRSGFGL**KR**ALDSLEGDGFGM**KR**ALDSFDGNGFGM**KR**ALDSLDGVGFGM**KKR**ALDSIEGNGFGFD**KR**ALDSLEGSDFGL**KKRSLNSMDGIGIDVGKNSFLITDPVASDHKTKKFACCTSTLCRLPAVSSSPSAA+

**Periviscerokinin**

*MCNTASRTGLLLVLIIVGCEC***VRQKRQDLIPSPRV**GKRVVLRGDGRGDVDILLKGLTDTGDTRPELLREEEDWTSFLTGQAGREPRQNTGNEWRHQTIAARRSHSSRSEQTPLTPAHLQALISTLYNCLQRMEALISTSEESDSRSKLAL-

**Pigment dispersing hormone (PDH)**

*MRSAVIVTMLVVVALAALLTQG*QDLKYQEREMVAELAQQIYRVAQAPWAGAVGPHKR**NSELINSILGLPKVMNDA**GRR-

*+SCVFVAVLVLVILAALVIEG*HELNVPEREAVATLAAHILKVVHAPLEAAAGLAHKR**NSELINSLLGLSRMMTQA**GRR-

*MRSCVFVAVLVLVVLADHVTQG*QELNVPQREAVATLAAHILKVIHAPQEAAAGLPHKR**NSELINS+**

*MRSCVFVAVLVLVVLADHVTQG*QELNVPQREAVATLAAHILKVVHAPQEPAEGFPHKR**NSELINSLLGLSRLTQA**GRR-

*MRYSVVVAVLLVVVFSVLFTQG*QELNLPEREALANLAAHILKLVRSSPEVGAGLPHKR**NSEIINSLLGLNRLINDA**GRR-

*CVFVAVLVLVILAALVIEG*HELNVPEREAVATLAAHILKVVHAPQEAAAGLPHKR**NSELINSLLGLSWVMNEA**GRR-

*SCVFVAVLVLVILAALVIEG*HELNVPEREAVATMAAHILKVVHAPQEAAAGLPHKR**NSELMNSLLGLSWVMNEA**GRR-

**elongated PDH**

*MTSVLRVMVVVMVVAILTTTVIS*HPTWIGDIANKEEDGEVRLMPVMMVAVPHLRSAHHTQPRPDQAHKR**NSELLNTLLGSQDLGNMRNA**GRR-

**Proctolin**

*MARSGLVLVVVALVVMAAAVCQA***RYLPT**RGDDSRLDEIRELLRELLERTAEGGVGGSSTNTGRMAYDKRFLFKRAAPVVAAGEVVEPLLNLPQ-

MAAAVCQA**RYLPT**RGDDSRLDEIRELLRELLERTAEGGVGGSSTNTGRMAYDKRFLFKRAAPVVAAGEVVEPLLNLPQ-

**Pyrokinin**

*MVFKSTRFFPVASVACLVLTSCVSLVAG*IGEGVSSSEWSAAPLSLRPSSPLGHGVSDAVTSFLPGSINTQNVMAYHTIPVRSSRPLRIQSPGVPKR**LYFAPRL**GKRSPSMAEVLDERGRRDAASTPEDLEDAPASPYSWWWPLVSVRRSSFSPRPGKRGEDEAEMDLPFEYYDEDEEETEDEDEDEDEGISLNRR**DTGFAFSPRL**GKR**DYKTTFAFGPRP**GKR**SNFAFAPRP**GKK**AETNFAFAPRP**GKR**TKFAFAPRP**GKR**TNFAFAPRP**GKK**NTFAFAPRP**GKK**TSFAFSPRL**GKK**ADFAFAPRP**GKRDSAHQTERQSGETWWLGESLGEEDTTTATTQAPFLPPRLE-

**Red pigment concentrating hormone (RPCH)**

MHVLFLFQSVVIVMFGGEEGHHYQLHLQVLNCLHPAPLLTTLNTEG*MVRRTGVTLLVVALVVVALVSSVSA***QLNFSPGW**GKRAAAGSGSSGGVGEAVSALHHSVGGAPGGVVPPGSSSSSGDSCGPIPVSAVMHIYRLIRNEAVRLVQCQDEEYLG-

**Relaxin-like**

*MCLLMVVVVVVVVDLSTC***IDPDLLSEIRNRNARDWQALWSEERLALCRLRLRHNLDAICGKDVY**RRSAPPAPPLQDEDEERKIQEEEEEDEEEEEGGVGEVGEKRGL+

+NREQVRQRDWKGWQEEEEGGVGEVGEKRGLGGGVSLHLPSTRIEINPPPPTPHYKDQGGDSRSPLLSVQQANLFVTTWIRDTPPPPKDNFGKPQIDPLRKGGGGSRWHRHPRR**SLYYPPHPRHARHAPSITSECCTEVGCTWEEYAEYCPSSSRLRPGVTLI**-

**RYamide**

*MKMPRALSPALVVLATLIAVAAS***QGFYSQRY**GKRGSDTRQVSER**SGFYANRY**GRSQGIPEIKVRSSR**FVGGSRY**GKRSGLPALAEVPLPVVAPESEEGEVGASLLLGDSVVCLLVDVPDIYRCLKKSASEESTN-

**short Neuropeptide F**

*MGMNSVKCWVALVCCCLLLCQLTTA*APADYDTMNEVYDWLAEHGVERR**APPSMRLRF**GKR**DMGWQVAQRSMPSLRLRF**GKRNVDQADPILDHHDLIRK**DARTPALRLRF**GKRGAPFGEEDVASQEQ-

**SIFamide**

*MSVQMRVVVAVAVVVVVLALLSSPVSA***GYRKPPFNGSIF**GKRSGGDAVYEPGKSQALASVCQVALEACTLWFPGAEKK-

**Sulfakinin**

*MWWAVTAAVVVVVAA*TGEVVVGGGGSLAARPLPPRPAVALARVLAPVVRHRLEEEEEGLLEELLEEEGGVAAGGPIEVLDAAGKR**EFDDYGHMRF**GKR**GSGGDDYQDDYGHLRF**GRSYHKANYHPHNNINNNDNNNFYHHRRFNS+

*MWWAVTAAVVVVVAA*TGEVVVGGGGSLAARPLPPRPAVALARVLAPVVRHRLEEEEEGLLEELLEEEGGVAAGGPIEVLDAAGKR**EFDDYGHMRF**GKRGSGGDDYQDDRSEER+

+VCSSDLGGPIEVLDAAGKR**EFDDYGHMRF**GKR**GGDDYQDDYGHLRF**GRSYHKANYHPHNNINNNDNNNFYHHRRFNS+

**+YGHLRF**GRSYHKANYHPHNNINNNDNNNFYHHRRFNSVRGGSGKV-

**Tachykinin-related peptide (TRP)**

*MARVWAWAVVVMVGVVAVAA*AGGTGEGAEEGSDAPRQRR**APSGFLGMR**GKKEAPSPPLQDTPTHPEDLMPAFYQLDMPLRGKK**APSGFLGMR**GKKNDEEEEEEEEEEREAIYGRPAFDND+

+**APSGFLGMR**GKKNDEEEEEEEEEEREAIYGRPAFDNDFETLLKR**APSGFLGMR**GKK**APSGFLGMR**GKKSMEEYLPSPSVSRQALLSLLQGQGAGDAAPNDDYYYYYYNPEAWRAGTHKR**APSGFLGMR**GKKDAYPAPAQDKR**TPSGFLGMR**G-

+MEVKIEEEKEEEEEREAIYGRPAFDNDFETLLKR**APSGFLGMR**GKK**APSGFLGMR**GKKSMEEYLPSPSVSRQALLSLLQGQGAGDAAPNDDYYYYYYNPEAWRAGTHKR**APSGFLGMR**GKKDAYPAPAQDKR**TPSGFLGMR**G-

+**APSGFLGMR**GKKNDEEEEEEEEEEREAIYGRPAFDNDFETLLKR**APSGFLGMR**GKK**APSGFLGMR**GKKSMEEYLPSPSVSRQALLSLLQGQGAGDAAPNDDYYYYYYNPEAWRAGTHKR**APSGFLGMR**GKKDAYPAPAQDKKDAYPA+

**Trissin**

*MQFSLALILAWAVVGGTWA***WSSSEVSCTSCGSECKSACGTRNFRACCFNFQ**RRRRSNVPLLSNQRDGVAAEWRALRGALMSPGGVPRSIPLTQFMEEAETDLVPRPPKHYRQPITLASVLTSLLQQSSEDEGDEDEMLELEGEGGGEGIPEGGTDDAALSRLVALALHQPPALPPRSSLRQHYPAHSPPPPPPAADVGK-

MSPGGVPRSIPLTQFMEEAETDLVPRPPKHYRQPITLASVLTSLLQQSSEDEGDEDEMLELEGEGGGEGIPEGGTDDAALSRLVALALHQPPALPPRSSLRQHYPAHSPPPPPPAADVGK-

**Putative mature peptides (bold) and precursor-related peptides of *Carcinus maenas***

| **Family** | **Peptide** |
| --- | --- |
| **ACP** | **pQITFSRSWVPQa** |
| ACP-PRP | SSPPPDIPEALDPCKDARVATITSLAGHLLDMMNEAAPADHRPLSDDTTPVLRLRNALMD |
|  | MA |
| **ALP** | **GGACDHRPNDCCYNSSCRCNLWGTNCRCQRMGLFQQWa** |
| ALP-PRP | QPLLEEGREPDGMQQAEVDYTADLLDHLLGRAQ |
|  | SPDIYLFRRSCI |
|  | EPDGMQQAEVDYTADLLDHLLGRAQ |
|  | SCI |
| **AST-A** | **HTPPRHSYAFGLa** |
|  | **APQPYAFGLa** |
|  | **GGPYSYGLa** |
|  | **SDMYSFGLa** |
|  | **SGSYNFGLa** |
|  | **PRAYSFGLa** |
|  | **PRGYAFGLa** |
|  | **PKVYAFGLa** |
|  | **QGAYSFGLa** |
|  | **PKVYSFGLa** |
|  | **LKAYSFGLa** |
|  | **QGPYAFGLa** |
|  | **GYEDEDEDRPFYALGLa** |
|  | **PRTYSFGLa** |
|  | **EPYEFGLa** |
|  | **EPYAFGLa** |
|  | **ASPYAFGLa** |
|  | **GGPYAFGLa** |
|  | **APTDMYSFGLa** |
|  | **DPYAFGLa** |
|  | **EAYAFGLa** |
|  | **PADLYEFGLa** |
|  | **AGPYAFGLa** |
|  | **AAPYAFGLa** |
|  | **PDMYAFGLa** |
|  | **ATGQYAFGLa** |
|  | **SGQYSFGLa** |
|  | **SGSYSFGLa** |
|  | **AGPYSFGLa** |
| AST-A-PRP | QLDEYDLQYDPNTLLQILQQYEAVAAARADEEQQDEDDEFGFa |
|  | SVRDVASEEEEEVKEEEEEEERQQELRKEEEKEAGa |
|  | S |
|  | DVSEEEEEENNMSTTSQYDTYN |
|  | KQDDVGFS |
|  | EYDDMYTE |
|  | GDDDADME |
|  | GDDAEME |
|  | GDDDVDLE |
|  | ADDSDFD |
|  | TSDDDTEEEEEEEEEDEDEEEEEATNYKa |
|  | AQGDAGDSYTLa |
|  | EAMDEEDAHTKMTTGNKASIDQSSSSSSSSSSSHSS |
| **AST-B/MIP** | **AGWSSMRGAWa** |
|  | **NNNWSKFQGSWa** |
|  | **GNWNKFQGSWa** |
|  | **AGWNKFQGSWa** |
|  | **TSWGKFQGSWa** |
|  | **NNWSKFQGSWa** |
|  | **AGWSSLQGAWa** |
|  | **AWSNLQGAWa** |
|  | **SGNWSNLRGAWa** |
|  | **PDYPAVSPRSTNWSSLRGTWa** |
|  | **VPNDWAHFRGSWa** |
| AST-B/MIP-PRP | QDEGSGLAVAQA |
|  | DDSSDHGLQISED |
|  | GEEIQDAEE |
|  | ANDMTEEAALQAAED |
|  | GDEITSEDDLQDAED |
|  | QDDLIQPQDLED |
|  | SPNDSEDIEDEALEEEELQVSPEVLARMVAAAPV |
|  | GWALWa |
|  | ANDMTEEAALQVSRGIGGAGGRLMKV |
|  | NPDTLISA |
| **AST-C** | **QIRYHQCYFNPISCF** |
|  | **GNGDGRLYWRCYFNAVSCF** |
|  | **SYWKQCAFNAVSCFa** |
| AST-C-PRP | LPAKEGPEAQKEVSSGHDGGRLT |
|  | AAPASDTSQEELAALKDLILSRLAAELEDSYQDLPSF |
|  | DLLKAGIDMQEDDEGDDEa |
|  | EV |
|  | MFAPLSGLPGNLRTI |
|  | +DLKAEGLSTLTTTTTTTAILPFSSIIVNVILLMTLLAPHVSPAHLLMSPRPHYIEEIRPVLPTNIPLMIRQQQQQQQPILAAPLQVQQEPRD+ |
|  | +IEEIRPVLPTNIPLMIRQQQQQQQPILAAPLQVQQEP |
|  | AAIVLDKLMFALQKALDEPSSPPHPQAAPLPPRTPYTGPVDLQ |
|  | KPLGDLDPSAGGPPPFSARQAQVYEPYGNNVEEDGSLDAALINYLFAKQLVQRLRNPSEVSRENQ |
| **Bursicon-α** | **DECSLRPVIHILSYPGCTSKPIPSFACQGRCTSYVQVSGSKLWQTERSCMCCQESGEREAAITLNCPKPRPGEPKEKKVLTRAPIDCMCRPCTDVEEGTVLAQEIANFIQDSPMDSVPFLK** |
| **Bursicon-β** | **RSYGVECETLPSTIHISKEEYDDTGRLVRVCEEDVAVNKCEGACVSKVQPSVNTPSGFLKDCRCCREVHLRARDITLTHCYDGDGARLSGAKATQHVKLREPADCQCFKCGDSTR** |
| **Calcitonin** | **TCYLNGGLSHGCDYQDLVSSTVEKNYWDSVNSPa** |
|  | **LCLNLGDPSCYEGNMAANGDDNNYLIGQNNPa** |
| Calcitonin-PRP | QPTHHESQEAYLSEKMREYLLLRRLLISVLSERQPPPMEAP |
|  | MAEEEEEI |
| **CCHamide** | **HGSVKLGCLNYGHSCLGAHa** |
| CCHamide-PRP | GSWSPSVPRPPAAALLAPFLQSLAAPRSPASSWQDKIMAQHRQHKNEQQQQQQQKQQQQQQQQQQQQ+ |
|  | AGDQYGNLDPADLYPPTNQLAEVDEARYPLDEAPVTNPRTLAKVRDLMDFMGHLLRQRAAPLQQQQQQQRAPLAAQLGPNDAYLH |
| **CCRFamide** | **APRPSLPRPGCDVEALRCDVICSVPDLGFHCTRCARRPMRFa** |
| CCRFamide-PRP | GGHVDPPLPSISSSSSVMPGGGG+ |
| **CFSH** | **SSIIGHMNSIPYRTREQVMEGMMDTYVLVPRAIISSTSKLHHDVNCSEYREVNKIHGNRFEANFNIRPTWLHRSKTISTCPTQYVERQIQGPFPIQPVTILEAKCVCEGSQCSQDGSICVAVKYRLPVWIRVDSDGYTTDTVELTVACACAKNPSRDGGYVDLSEN** |
|  | **GALCRGAGRRACRRGAVTNIPVAEVTKNWEAEYLSIPESLVSFSQKQTEEAVCKDLSVQLFEVDLWEEDLKPLWVRKTVHLGVCPSMLQERRLGNDVWPSRVVEVKCLCQQQSCSSFGGDFRCQAVRRSVRTWVRSSEKTFVPSQEIVSVGCVCVQRTSTQARLANLVEG** |
|  | **DPNSSTLSDNDFYDDILSHLDGNNPNDNSSLEPLSDKLQPENPCPPYSLFEDVFNQYTSVPRFLLTLARHDACQGLEGSAQKVQLQDYSGFRPHWLVDATTAGECPWHLVRREFLHDTLPPAILEVSCLCDGHRCSLRGDFECISVKQQVIVWSSESTGGSHYQPRLLQVTAACVCAQRHAPQASHARPGQWK** |
| CFSH-PRP | YTPVYPSRVQHFLQYLQQERQEDGLPLTTQSP |
|  | TAVSNALGSREGAVQQEQQEQQVAPFVVPQWWWLSSLLSLPHGHHGYTTPADAAEAAAAARKVAGDTEESPVSEDEASTLSLMLPLEQLTDGKESVSSPSVA |
| **CNMamide** | **VMCHFKICNMa** |
| CNMamide prp | YPGYVDRLPSL |
|  | YPQTQQQQQALGEGEPFAGANDDYVEDDDDLGYSKKYAYNTLMAGREPDIFPYDQAEASNKNLQQILRSFRPVVGGTYPSDRVLPWDDLHELGLKDGRSAEEQLSAPDTSLTQLSFAKHRQ |
|  | ARHSNPLQGWLS |
| **Corazonin** | **QTFQYSRGWTNa** |
| Corazonin-PRP | SNDLGTVVGLGSGRRTGVNLLPAEA |
|  | SLQQQTTPHRSLP |
|  | DVEERL |
|  | AVEAGVSALLHAAQQNPEAPPATPGEQDYYAQN |
| **DH44** | **NTGLSLSIDASMKVLREALYLEIARKKQRQQLQRAQHNKALLNTIa** |
| DH44-PRP | +NNNNNNNSLTPSTTTTTTNSGTSTTANNPRAIPHSAPMF |
|  | AWQHSFS |
|  | DVASQLVGVDQSVSLDNDRN |
| **CCAP** | **PFCNAFTGCa** |
| CCAP-PRP | GPVA |
|  | DIDSLLDGKI |
|  | SDPELEGLASGSELNDITKHVLAEARLWEQLQSKMEAMRMLASRMDSRPVF |
|  | SLIHPQHDRVHSVTTLNHKGDAEKQ |
|  | SLIHPQHDRVHSVTTLNHKNSGATMLKHIYNRGTVCTIDYNHKGSETLRNSEDQKTQPTAFINLIK |
|  | SLIHPQHDRVHSVTTLNHKASLMQFYFGNSLSHLMDGIGSTNHVVHLPKP |
|  | AFCDPVILLPSLSLSLSLSLSLCNFCAPYFFSSWASN |
| **CHH** | **QIYDTSCKGVYDRALFNDLEHVCDDCYNLYRTSYVASACRNNCFENEVFDVCVYQLYFPNHEEYLRSRDGLKG** |
|  | **QIYDTSCKGVYDRALFNDLEHVCDDCYNLYRTSYVASACRSNCYSNLVFRQCMDDLLMMDEFDQYARKVQMVa** |
|  | **QQHRRSRRALIGVHTRCKGKYSREIWNELYYVCEDCDNLYRKNGFQAECRRNCFDTDIFATCLSVLGKDEKMYQAMATALRGS** |
|  | **QQHRRSRRALIGVHTRCKGKYSREIWNELYYVCEDCDNLYRKNGFQAECRLGSSDLIFDDDGRVSWSGVLNEP** |
| CHH-PRP | RSTPGYGRMDRILAALKTSPMEPSAALAVEHGTTHPLE |
|  | GPVKSLHTSSLAQSPALPREa |
| **CHH/MIH-like** | **SPTTSSSSFKDKSYQFRLWPGTQREFEFYHCGGEFDAASRKLYSHLSAVCEDCKNYYRSEPLLRQRCMSKCFDNEIFTYCAETLYSIDKFKDYEEMKVQIKA** |
| **DH31** | **GLDMGLGRGFSGSQAAKHLMGLAAANYAGGPa** |
| DH31-PRP | SPLNREPSRAVVEIDDPDYVLELLTRFSNSII |
|  | AKELEKFVRSSSGT |
|  | DSQATTLDLHHDDQHLAQHEHAAAAVAAAGLPQHSS |
| **EH** | **+LAAVTDAHADEMYGDYFHGQACAESCIRTQGMTIPDCNNPATFNRFL** |
|  | **AVAANRKVSICIKNCGQCKKMYTDYFNGGLCGDFCLRTKGRFIPDCNRPDILIPFFLQRLE** |
| EH-PRP | FI |
| **ETH** | **DAGHFFAETPKHLPRIa** |
| ETH-PRP | GDLPPLTTLLSEEDARSSSAGTQSMTEALAGLDSDGDGCIGIGELLRIPAVRVALLLQNPALLTPANPATPEHDGHATEDTFTSDRRPEPRLLRYLQK |
|  | GDLPPLVSERVDVLRSDVLGIVW |
| **ELFamide** | **AGRDPMLRYLLVAMAQPGPRYAAPQILSRGVRRMGSEFLa** |
|  | **AIGSLPRSHLARYFSLLMNRKMGSEFLa** |
|  | **AMGSEFLa** |
| ELFamide-PRP | ALQTTHPDESVSDVS |
|  | SVAVSPESRKMCESGSCVTKEDQHHTDDLRKEQMSFTGQYNEQNLDDDHEDGM |
| **Elevenin-like peptide** | **IDCRRFVFAPQCRGIIA** |
| Elevenin-like peptide-PRP | TVSDAAIGLPHVLQEQRQWPESIPVDYVVPEYSPVYQRVRSTYTRPNAASRDVSVGAQFPSPNAAMTEMDVRESGDMELLSPYVKVIHRSENLPYQ |
| **FMRFamide** | **GISRNYLRFa** |
|  | **GGRNFLRFa** |
|  | **NRNFLRFa** |
|  | **DRNFLRFa** |
|  | **AAHKNYLRFa** |
|  | **GNRNFLRFa** |
|  | **DGNRNFLRFa** |
|  | **GYNRSFLRFa** |
|  | **APQGNFLRFa** |
| FMRFamide-PRP | SPLNTVPGAIEASPSTHNDPGSDVPEIPQE |
|  | LLKYFLPSGSSWMSTQQEGS |
|  | GEED |
|  | GDSSSIEDVEVLPETEDSIE |
|  | SDAEEFSLPGGPLAFTNVHEQDLEEYPTEEE |
|  | SVDRQLNEQKARDAPLAPTTVPHSSAKTQESHRS |
|  | SATPYNYVVLPSHGPAAWSQDFQPDQDEDLEEAIEGPESAMT |
|  | NDESDPQMVMMEPASYPRYV |
| **GPA2** | **HQHTWQTPGCHKVGHTRRISIPECVEFDITTNACRGYCESWSVPSAWQTLVYNPHQVVTSIGQCCNIMETEDVKVKVMCVEGPRELIFKSASTCDCFHCKKY** |
| **GPB5** | **IDPQSTLECHRRQYSYKVHKTDDDGRMCWDVVNVMSCWGRCDSNEIADWKFPYKRSHHPVCIHDETQITEVTLRNCDEDVAPGTELYSYHEATRCACSVCKSSQASCEGLRYRGARRAPRAQVPRa** |
| **HIGSLYamide** | **HIGSLYRa** |
|  | **HVGSLYRa** |
| HIGSLYamide-PRP | +MFYRQD |
|  | HYGSLLRNGPLPFMQED |
|  | HFGSLLKSPRYRAISIPa |
|  | DSPAVDEQQEDTVRQLEEARELSKQQFSSLLRNQFDDDLELQQRLLAAGLISPRDTQELAALHSTWNPATSPGQD |
|  | DDATLYDLSEE |
|  | DDDSLYDLSED |
|  | DEDSLYDLSED |
|  | DEDSLYHELSED |
|  | DEDNDLGLYED |
|  | DDDDAQALSED |
|  | DENNLNQDLSED |
|  | DEDNSQGLFED |
|  | DEDSTQNLAED |
|  | DEDNSQTLAED |
|  | DEDNSQNLSED |
|  | DEDNTNNLSED |
|  | DEDNSPNLSED |
|  | DGGNTSTFSED |
|  | DDDTNSLSED |
|  | DEDNANDLSED |
|  | +NSISED |
|  | DEDNAHDLSED |
|  | HQQDFPDLIQDEEAIERRLHELPD |
|  | YFASLLRNRPSAFGPWNPPD |
|  | HIGSFFQNRQVPFGMQDSTE |
|  | HIGSFLQHRPSAFVPD |
|  | HIGSFLQNRQLPFASHDQSAE |
|  | HIGSFFQNRQSGFGTQDLSAD |
|  | HVGSFFQNRQSPLGLQDSAE |
|  | HISSFMQDQGPPFAPDQGSGL |
|  | YIGALARTNNMPSPYSRARQNRRDAPDPSSLRAHLLQRLLNMERQRDFLRQRQQQALITRTQNAAMMLKYLEHAGLDGEDVALDLQPEQDLGVDLALDEHREPGGDLDHLQYTMGGATPLEDNALTYHSAN |
|  | FLGSLARSGWFPRRYSGAYSFDLQ |
|  | DLASQMTLDEDDEDEEEYEEENKPFFTFSY |
|  | FLGE |
| **Hyrg** | **QQKPRILLEDPTNLQNQPWVQPPRFHYRGFNRPQVGAGWP** |
| **Insulin-like** | **SPRTLPGQGLVTDGERRLCGWRLANELNRVCKGVYNKPTVSNNALFYLRGRGV** |
|  | **GLSAECCRKACSVSELAGYCY** |
| Insulin-like-PRP | VDLMPLGMELEFSPWTQASADDLRSDQISESHTSHIPKQLPFLAEAEASRVVGGLPRV |
| **Intocin** | **SCFITNCPPGa** |
| Intocin-PRP | SGGLMSTLGRARTCASCGPGLLGRCLGPDICCGARIGCFLGSRET |
|  | MCRTENMVPITCYNSDLKPCGRMKEGLCAAPGLCCTENKCEMNDDCVVEDMRGEEVAASQRGA |
|  | SRLDLLTAARDRWEEQ |
| **Leucokinin** | **APFHAWAa** |
|  | **FRSWGa** |
|  | **AFSAWAa** |
|  | **GGGFSAWAa** |
|  | **SEEDERKQAFSAWAa** |
|  | **QGFSAWPa** |
|  | **KFNAWAa** |
| Leucokinin-PRP | QEIPSDQDSSLLPCSLASPDESLRCEV |
|  | GQRFTWIGHPPSPSSLRDVYMRLVRHGRLPQNAILALDL |
|  | SSFTLPSPSVLHIGEEVPFSTLEEGDEEEEEQEEWTTLLDPTAKLLSLEGDEMASTLVPLLPPAMSHVRV |
|  | STEKQPNTLTNSYIEGSRKP |
|  | SGQERGKLEDVLRAWNTQGDGMAWSANMKVNADT |
|  | SYDI |
|  | EAFDSWNa |
|  | SDDE |
|  | QAFNAWAA |
|  | SDNENDND |
|  | SYDDAFQEQKEEMEKSKGQLSSLVPQHQQQEHQQVSELLHHTPDSLTHWDANWD |
| **MIH** | **RVINDECPNLIGNRDLYKIVDWICEDCANIFRKNGMANLCRRNCFYNNDFLWCIIATENTRHEKKLKEWATILRAGARL** |
|  | **RSNNDDCPNVIGNRDLYKIVDWICEDCANIFRKNGMANLCRRNCFYNNDFLWCIIATENTRHEKKLKEWATILRAGARL** |
|  | **+GNRDLYKKVEWICEDCSNIFRKTGMASLCRRNCFFNEDFVWCVHATERSEELRDLEEWVGILGAGRD** |
| **Myosuppressin** | **QDLDHVFLRFa** |
| Myosuppressin-PRP | IPPPICFNQKLVLTPYARRLCAALNDISKFSRAMEDYLDAQAIKNSMGVNEPEV |
|  | AQQ |
| **Natalisin** | **DGRGPFWAARa** |
|  | **TNPFWISRa** |
|  | **EEGGPFWISRa** |
|  | **PQPGSAESQLASLWAIRa** |
|  | **SGADNTFWVARa** |
|  | **ETNAQGPFWAARa** |
|  | **SGSDSETGPYWIARa** |
|  | **QDGTSSSNGPYWIARa** |
|  | **EDDGAVFWAARa** |
|  | **DSQTNGRSEDAAHSFWVARa** |
| Natalisin-PRP | AQEFSPGDGGSSELYRHS |
|  | SVEVGGDASWLPSLQDDQLTQVESRQDAGQGGTTFWVARa |
|  | DADGSTSYYWGPNQGLWGEAFQRGAPAMSSSSRVPLLIRSGWESNPSLWa |
|  | DSSEASPSLLQQWMAEEPTMQNQKEERVWSGES |
|  | NSHATSHSTEKDNDDDDDDDDDEDEHHDEATQKATDHHY+ |
| **Neuroparsin** | **APRCDRHDEEAPKNCKYGTTQDWCKNGVCAKGPGETCGGYRWSEGKCGEGTFCSCGICGGCSPFDGKCGPTSIC** |
|  | **GPICASLNEVLPEMLQAPCKHGVVMDWCGNARCAKGPGQTCGGRWNEHGTCGKGMYCVCGYCAGCSTDLQCALGRFC** |
|  | **GPICASLNEVLPEMLQAPCKHGVVMDWCGNARCAKVRRFID** |
|  | **APQCSSYDQEPPKNCKYGTALDWCRNGVCAKGPGESCGGYRREQGICGEGTYCECGRCRGCSPFDTSCHDAVLC** |
|  | **VPLCGNISDAELSQCKHGTVRNRCGKTVCAKVSNPDSLSSYTSLESVTRQKIIHQDF** |
|  | **VPLCGNISDAELSQCKHGTVRNRCGKTVCAKGLGEACMVYRWERDLCGIGTFCGCGFCQGCNTSLQCWEC** |
| **NF** | **KPDPTQLAAMADALKYLQELDKYYSQVSRPRFa** |
|  | **KPDPTQLAAMADALKYLQELDKYYSQVSRPSPRSAPGPASQIQALEKTLKFLQLQELGKMYSHMARPRFa** |
|  | **AQLPSRQDGSALEALQALHEAALAGTLSSAEVQYPSRPNVFKSPGELRRYLDALNAYFAIAGRPRFa** |
| NF-PRP | SEMVLPPGDALMEASERLLETLA |
|  | SEMVLPPGDALVSVRa |
|  | GKQVRQPEELYDY |
| **Orcokinin-A** | **NFDEIDRSSFGFN** |
|  | **NFDEIDRSGFGFA** |
|  | **NFDEIDRSGFGFA** |
|  | **NFDEIDRSSFGFN** |
|  | **NFDEIDRSSFGFV** |
|  | **NFDEIDRSGFGFV** |
| Orcokinin-A-PRP | AIKDAPAHPSNHPDTGYASDGSSA |
|  | FDAFTTGFGHS |
|  | MLTPRDLANLY |
|  | NAE |
| **Orcokinin-like** | **ALDSIDRSGFGL** |
|  | **ALDSLEGDGFGM** |
|  | **ALDSFDGNGFGM** |
|  | **ALDSLDGVGFGM** |
|  | **ALDSIEGNGFGFD** |
|  | **ALDSLEGSDFGL** |
| Orcokinin-like-PRP | +EGSDFGL |
|  | SLNSMDGIGIDVGKNSFLITDPVASDHKTKKFACCTSTLCRLPAVSSSPSAA+ |
| **Periviscerokinin** | **VRQKRQDLIPSPRVa** |
| Periviscerokinin-PRP | VVLRGDGRGDVDILLKGLTDTGDTRPELLREEEDWTSFLTGQAGREP |
|  | QNTGNEWRHQTIAARRSHSSRSEQTPLTPAHLQALISTLYNCLQRMEALISTSEESDSRSKLAL |
| **PDH** | **NSELINSILGLPKVMNDAa** |
|  | **NSELINSLLGLSRMMTQAa** |
|  | **NSELINSLLGLSRLTQAa** |
|  | **NSEIINSLLGLNRLINDAa** |
|  | **NSELINSLLGLSWVMNEAa** |
|  | **NSELMNSLLGLSWVMNEAa** |
| PDH-PRP | QDLKYQEREMVAELAQQIYRVAQAPWAGAVGPH |
|  | HELNVPEREAVATLAAHILKVVHAPLEAAAGLAH |
|  | QELNVPQREAVATLAAHILKVIHAPQEAAAGLPH |
|  | QELNVPQREAVATLAAHILKVVHAPQEPAEGFPH |
|  | QELNLPEREALANLAAHILKLVRSSPEVGAGLPH |
|  | HELNVPEREAVATLAAHILKVVHAPQEAAAGLPH |
|  | HELNVPEREAVATMAAHILKVVHAPQEAAAGLPH |
| **ePDH** | **NSELLNTLLGSQDLGNMRNAa** |
| ePDH-PRP | HPTWIGDIANKEEDGEVRLMPVMMVAVPHLRSAHHTQPRPDQAH |
| **Proctolin** | **RYLPT** |
| Proctolin-PRP | GDDSRLDEIRELLRELLERTAEGGVGGSSTNTGRMAYD |
|  | FLF |
|  | AAPVVAAGEVVEPLLNLPQ |
| **Pyrokinin** | **LYFAPRLa** |
|  | **DTGFAFSPRLa** |
|  | **DYKTTFAFGPRPa** |
|  | **SNFAFAPRPa** |
|  | **SNFAFAPRPa** |
|  | **TKFAFAPRPa** |
|  | **TNFAFAPRPa** |
|  | **NTFAFAPRPa** |
|  | **TSFAFSPRLa** |
|  | **ADFAFAPRPa** |
| Pyrokinin-PRP | IGEGVSSSEWSAAPLSLRPSSPLGHGVSDAVTSFLPGSINTQNVMAYHTIPVRSSRPL |
|  | IQSPGVP |
|  | SPSMAEVLDERa |
|  | DAASTPEDLEDAPASPYSWWWPLVSVRRSSFSPRPa |
|  | GEDEAEMDLPFEYYDEDEEETEDEDEDEDEGISLN |
|  | DSAHQTERQSGETWWLGESLGEEDTTTATTQAPFLPPRLE |
| **RPCH** | **QLNFSPGWa** |
| RPCH-PRP | AAAGSGSSGGVGEAVSALHHSVGGAPGGVVPPGSSSSSGDSCGPIPVSAVMHIYRLI |
|  | NEAVRLVQCQDEEYLa |
| **Relaxin-like** | **IDPDLLSEIRNRNARDWQALWSEERLALCRLRLRHNLDAICGKDVY** |
|  | **SLYYPPHPRHARHAPSITSECCTEVGCTWEEYAEYCPSSSRLRPGVTLI** |
| Relaxin-like-PRP | SAPPAPPLQDEDEERKIQEEEEEDEEEEEGGVGEVGE |
|  | +NREQVRQRDWKGWQEEEEGGVGEVGE |
|  | GLGGGVSLHLPSTRIEINPPPPTPHYKDQGGDSRSPLLSVQQANLFVTTWIRDTPPPPKDNFGKPQIDPLRKGGGGSRWHRHP |
| **RYamide** | **QGFYSQRYa** |
|  | **SGFYANRYa** |
|  | **FVGGSRYa** |
| Ryamide-PRP | GSDTRQVSE |
|  | SQGIPEIKVRSS |
|  | SGLPALAEVPLPVVAPESEEGEVGASLLLGDSVVCLLVDVPDIYRCLKKSASEESTN |
| **sNF** | **APPSMRLRFa** |
|  | **DMGWQVAQRSMPSLRLRFa** |
|  | **DARTPALRLRFa** |
| sNF-PRP | APADYDTMNEVYDWLAEHGVE |
|  | NVDQADPILDHHDLI |
|  | GAPFGEEDVASQEQ |
| **SIFamide** | **GYRKPPFNGSIFa** |
| SIFamide-PRP | SGGDAVYEPGKSQALASVCQVALEACTLWFPGAE |
| **Sulfakinin** | **EFDDYGHMRFa** |
|  | **GSGGDDYQDDYGHLRFa** |
| Sulfakinin-PRP | TGEVVVGGGGSLAARPLPPRPAVALARVLAPVVRHRLEEEEEGLLEELLEEEGGVAAGGPIEVLDAAG |
|  | GSGGDDYQDDRSEER+ |
|  | SYHKANYHPHNNINNNDNNNFYHHRRFNSVRGGSGKV |
| **TRP** | **APSGFLGMRa** |
|  | **TPSGFLGMRa** |
| TRP-PRP | AGGTGEGAEEGSDAPRQ |
|  | EAPSPPLQDTPTHPEDLMPAFYQLDMPLRa |
|  | NDEEEEEEEEEEREAIYGRPAFDNDFETLL |
|  | SMEEYLPSPSVSRQALLSLLQGQGAGDAAPNDDYYYYYYNPEAWRAGTH |
|  | DAYPAPAQD |
|  | +MEVKIEEEKEEEEEREAIYGRPAFDNDFETLL |
| **Trissin** | **WSSSEVSCTSCGSECKSACGTRNFRACCFNFQ** |
| Trissin-PRP | SNVPLLSNQRDGVAAEWRALRGALMSPGGVPRSIPLTQFMEEAETDLVPRPPKHYRQPITLASVLTSLLQQSSEDEGDEDEMLELEGEGGGEGIPEGGTDDAALSRLVALALHQPPALPPRSSLRQHYPAHSPPPPPPAADVGK |
